# Supplementary material for: Delphi consensus study to develop guidelines for the management of adults with borderline personality disorder in the emergency department: a protocol
Source: BMJ Open. 2023 Nov 9;13(11):e075119. doi: 10.1136/bmjopen-2023-075119 (PMC10649594; doi:10.1136/bmjopen-2023-075119)
Supplement: Supplementary data [file bmjopen-2023-075119supp001.pdf]

BMJ Publishing Group Limited (BMJ) disclaims all liability and responsibility arising from any reliance placed on this supplemental material which has been supplied by the author(s)

Supplementary Materials

Protocol for a Delphi consensus study to develop guidelines for the management of adults with borderline personality disorder in the emergency department

APPENDIX A

Risk of bias mitigation

PL and VH will be participants in the Delphi process given their recognized expertise. This means there is a potential source of bias due to their involvement in developing GPM[1 2] and GPM applications to ED settings.[3] This will be mitigated by ensuring they have no access to the data or involvement in the data analysis. The chair of the consensus meeting will have no affiliation with a treatment approach.

Prosser A, *et al. BMJ Open* 2023; 13:e075119. doi: 10.1136/bmjopen-2023-075119

APPENDIX B

Protocol for systematic reviews

1. Review questions

We will conduct ten systematic reviews to search the literature for articles on the following key questions (KQs) related to the key areas of care of adults with borderline personality disorder (BPD) in the emergency department (ED):

1. What psychosocial and pharmacological interventions in the ED are associated with benefit while minimising harm for adults with BPD, with considerations regarding potential comorbidities?
    a. What interventions in the ED are effective?
    b. What interventions in the ED are harmful?
2. What are the effective approaches to assess and manage the risk of suicide and self-harm of adults with BPD, with considerations regarding potential comorbidities?
3. What should be included as part of the emergency evaluation of adults to screen for previously undiagnosed BPD?
4. What is the role of hospitalisation in managing adults with BPD?
    a. What are the indications for hospitalisation?
    b. What is the effectiveness of hospitalisation?
    c. What are the harms of hospitalisation?
5. What psychosocial aftercare services (including partial hospitalisation) can help adults with BPD who present to the ED but are not hospitalised?
6. What interventions in the ED for agitation and aggression are associated with benefit while minimising harm for adults with BPD?
    a. What interventions for agitation and aggression are effective?
    b. What interventions for agitation and aggression are harmful?
7. What are effective approaches to involving family members and support persons in the care of adults with BPD in the ED?
8. What are effective approaches to involving outpatient providers in the care of adults with BPD in the ED?
9. What are effective approaches to providing optimal medical care for adults with BPD presenting in a mental health crisis to the ED ?
10. What are the most effective environmental elements (physical layout and interior design) for adults with BPD in the ED?

2. Searches

The following databases will be searched from inception: MEDLINE, MEDLINE In-Process, Embase, Global Health, and PsychInfo with no language restrictions. The results of the individual databases will be transferred into a reference manager program and duplicates will be eliminated. To search for additional reports, we will search the reference lists of the included studies. A PRISMA flow chart will be included in the final publication.

3. Search strategy

KQ1

1. ((borderline$ or cluster$) adj3 personalit$).mp
2. (borderline$ or BPD).mp
3. ((difficult$ or challeng$) adj3 patient$).mp
4. (recurrent$ or repeat$) adj3 (suicid$ or self-harm$ or self-injur$ or self-cut$ or mutilat$).mp
5. or/1-4
6. (emergency adj3 (department$ or room$ or treatment$ or medicine$ or health service$)).mp
7. 5 and 6

KQ2

1. ((borderline$ or cluster$) adj3 personalit$).mp
2. (borderline$ or BPD).mp
3. ((difficult$ or challeng$) adj3 patient$).mp
4. (recurrent$ or repeat$) adj3 (suicid$ or self-harm$ or self-injur$ or self-cut$ or mutilat$).mp
5. or/1-4
6. ((suicid$ or self harm$ or self injur$ or self cut$ or mutilat$ or NSSI or DSH) and (risk$ adj5 (assess$ or evaluat$))).mp
7. 5 and 6

KQ3

1. ((borderline$ or cluster$) adj3 personalit$).mp
2. (borderline$ or BPD).mp
3. ((difficult$ or challeng$) adj3 patient$).mp
4. (recurrent$ or repeat$) adj3 (suicid$ or self-harm$ or self-injur$ or self-cut$ or mutilat$).mp
5. or/1-4
6. (assessment$ or screening$ or diagnostic$).mp
7. 5 and 6

KQ4

1. ((borderline$ or cluster$) adj3 personalit$).mp
2. (borderline$ or BPD).mp
3. ((difficult$ or challeng$) adj3 patient$).mp
4. (recurrent$ or repeat$) adj3 (suicid$ or self-harm$ or self-injur$ or self-cut$ or mutilat$).mp
5. or/1-4
6. (hospitalization$ or hospitalisation$ or admit$ or admission$).mp
7. (((single$ or double$ or trebl$ or tripl$) adj5 (blind$ or mask$)) or trial$ or (clinic$ adj2 trial$) or open-label$ or openlabel$ or (pre$ adj5 post$) or (pretest adj5 posttest) or cohort or (program$ adj5 evaluate$) or ((assurance$ or improv$ or indicator$ or measure$ or metric$) adj2 quality)).mp

8. 5 and 6 and 7

KQ5
1. ((borderline$ or cluster$) adj3 personalit$).mp
2. (borderline$ or BPD).mp
3. ((difficult$ or challeng$) adj3 patient$).mp
4. (recurrent$ or repeat$) adj3 (suicid$ or self-harm$ or self-injur$ or self-cut$ or mutilat$).mp
5. or/1-4
6. (emergency adj3 (department$ or room$ or treatment$ or medicine$ or health service$)).mp
7. (aftercare$ or (discharge adj5 (plan$ or service$ or manag$ or treatment$ or therap$))).mp
8. 5 and 6 and 7

KQ6
1. ((borderline$ or cluster$) adj3 personalit$).mp
2. (borderline$ or BPD).mp
3. ((difficult$ or challeng$) adj3 patient$).mp
4. (recurrent$ or repeat$) adj3 (suicid$ or self-harm$ or self-injur$ or self-cut$ or mutilat$).mp
5. or/1-4
6. (agitat$ or aggress$ or violen$ or anxiet$ or anxious$).mp
7. (((single$ or double$ or trebl$ or tripl$) adj5 (blind$ or mask$)) or trial$ or (clinic$ adj2 trial$) or open-label$ or openlabel$ or (pre$ adj5 post$) or (pretest adj5 posttest) or cohort or (program$ adj5 evaluate$) or ((assurance$ or improv$ or indicator$ or measure$ or metric$) adj2 quality)).mp
8. (emergency adj3 (department$ or room$ or treatment$ or medicine$ or health service$)).mp
9. 5 and 6 and 9 and 8

KQ7
1. ((borderline$ or cluster$) adj3 personalit$).mp
2. (borderline$ or BPD).mp
3. ((difficult$ or challeng$) adj3 patient$).mp
4. (recurrent$ or repeat$) adj3 (suicid$ or self-harm$ or self-injur$ or self-cut$ or mutilat$).mp
5. or/1-4
6. (emergency adj3 (department$ or room$ or treatment$ or medicine$ or health service$)).mp
7. (famil$ or partner$ or caregiver$ or parent$ or guardian$ or children$ or support$).mp
8. 5 and 6 and 7

KQ8
1. ((borderline$ or cluster$) adj3 personalit$).mp
2. (borderline$ or BPD).mp

Prosser A, *et al. BMJ Open* 2023; 13:e075119. doi: 10.1136/bmjopen-2023-075119

3. ((difficult$ or challeng$) adj3 patient$).mp
4. (recurrent$ or repeat$) adj3 (suicid$ or self-harm$ or self-injur$ or self-cut$ or mutilat$).mp
5. or/1-4
6. (emergency adj3 (department$ or room$ or treatment$ or medicine$ or health service$)).mp
7. ((outpatient$ or ambulator$) adj5 (provider$ or team$ or caregiver$ or treater$ or physician$ or psychiatrist$ or psychologist$)).mp
8. 5 and 6 and 7

KQ9
1. ((borderline$ or cluster$) adj3 personalit$).mp
2. (borderline$ or BPD).mp
3. ((difficult$ or challeng$) adj3 patient$).mp
4. (recurrent$ or repeat$) adj3 (suicid$ or self-harm$ or self-injur$ or self-cut$ or mutilat$).mp
5. or/1-4
6. (emergency adj3 (department$ or room$ or treatment$ or medicine$ or health service$)).mp
7. ((medical$ or physical health$) adj3 (care$ or treatment$ or management$)).mp
8. 5 and 6 and 7

KQ10
1. ((borderline$ or cluster$) adj3 personalit$).mp
2. (borderline$ or BPD).mp
3. ((difficult$ or challeng$) adj3 patient$).mp
4. (recurrent$ or repeat$) adj3 (suicid$ or self-harm$ or self-injur$ or self-cut$ or mutilat$).mp
5. or/1-4
6. (emergency adj3 (department$ or room$ or treatment$ or medicine$ or health service$)).mp
7. (environment$ or architecture$ or design$ or layout$ or decor or ergonomic$).mp
8. 5 and 6 and 7

4. Condition or domain being studied

We will study the key areas of emergency care of adults with BPD defined by our KQs.

5. Article inclusion/exclusion criteria

Inclusion: the report is (1) a primary study (clinical trial, cohort study, cross-sectional study, pre/post evaluation, program evaluation, or quality assurance), guideline, standards, consensus statement, decision-making tool, assessment/diagnostic/screening tool, or expert opinion, (2) includes a sample of at least >75% adults with BPD in an emergency setting (if a primary study) or is about adults with BPD in emergency settings (if a guideline, standard, consensus statement, decision-making tool, assessment/diagnostic/screening tool, or expert opinion), and is about

either (i) the overall care of adults with BPD in the ED using psychosocial and/or pharmacological interventions (KQ1), (ii) suicide/self-harm risk assessment and/or management of adults with BPD (KQ2), (iii) diagnostic screening for BPD, (iv) hospitalisation of adults with BPD (KQ4), (v) psychosocial aftercare services (including partial hospitalisation) of adults with BPD who present to the ED but are not hospitalised (KQ5), (vi) the pharmacological and/or non-pharmacological management of agitation/aggression in adults with BPD (KQ6), (vii) approaches to involving family members/support persons in the care of adults with BPD in the ED (KQ7), (viii) approaches to involving outpatient providers in the care of adults with BPD in the ED (KQ8), (ix) general medical care of adults with BPD presenting to the ED in a mental health crisis (KQ9), and (x) environmental elements (physical layout and interior design) of the ED in relation to adults with BPD presenting to the ED in a mental health crisis (KQ10).
Exclusion: none.

6. Main outcome(s)

The primary outcome for the primary studies is efficacy which will be categorized as follows. These outcomes will be summarized narratively along with an effect size if it can be calculated from the report. If an effect size cannot be calculated from the report, the outcome will be narratively summarized only.

1. *Suicidality outcomes*
    - i. Self-harm
    - ii. Suicidal ideation
    - iii. Suicide attempt
    - iv. Death by suicide
2. *BPD-related outcomes*
    - i. Emotion dysregulation
    - ii. Depression
    - iii. Anxiety
    - iv. Anger/irritability
    - v. Aggression
    - vi. Paranoia
    - vii. Dissociation
    - viii. Interpersonal dysfunction
    - ix. Self dysfunction (e.g., identity disturbance)
3. *Global outcomes*
    - i. Quality of life
    - ii. Global psychosocial functioning
    - iii. Global symptom severity
4. *Service utilization outcomes*
    - i. Patient satisfaction
    - ii. Hospitalisation
    - iii. ED visits
    - iv. Adherence with medications
    - v. Adherence with appointments.
    - vi. Time to all-cause treatment discontinuation

We will exclude measures when they cannot be categorized. Note, suicidal ideation, planning, and behaviour (including self-harm) will be always coded under behavioural dyscontrol, even if in the report it may have been coded as a tolerability outcome. The primary outcome for non-primary studies (i.e., guideline, standards, consensus statement, decision-making tool, assessment/diagnostic/screening tool, or expert opinions) will be a narrative summary of the opinion on a given KQ.

Measures of effect: See Additional outcomes section below.

7. Additional outcome(s)

The secondary outcome for the primary studies is tolerability which will be categorized as follows. These outcomes will be summarized narratively along with an effect size if it can be calculated from the report. If an effect size cannot be calculated from the report, the outcome will be narratively summarized only. There will be no secondary outcomes for non-primary studies.

- Any adverse event
- Serious adverse event
- Death (non-suicide)
- Nausea
- Constipation
- Diarrhea
- Dry mouth
- Headache
- Dizziness
- Sedation
- Sleep disturbance
- Restlessness/agitation
- Menstrual changes
- Increased appetite
- Weight gain
- Decreased appetite
- Weight loss
- Extrapyramidal symptoms
- Akathisia
- Tardive dyskinesia
- Tremor (any type)
- Any other neurological dysfunction
- Cognitive dysfunction
- Sexual dysfunction
- Systolic blood pressure
- Diastolic blood pressure
- QTc interval
- Liver enzymes (AST, ALT, ALP, or GGT)
- Bilirubin (total, direct, or indirect)
- Albumin
- International normalized ratio (INR)
- Low-density lipoprotein (LDL)
- High-density lipoprotein (HDL)
- Triglycerides
- HbA1C
- Leukocyte count
- Hemoglobin
- Platelet count
- Creatinine
- Urea or blood urea nitrogen (BUN)
- Estimated glomerular filtration rate
- Sodium
- Potassium
- Calcium
- Phosphate
- Magnesium
- Creatine kinase
- Prolactin
- Thyroid stimulating hormone
- Thyroid hormone

Prosser A, *et al. BMJ Open* 2023; 13:e075119. doi: 10.1136/bmjopen-2023-075119

- Weight loss
- Endpoint weight
- Total cholesterol
- Fasting blood glucose

Measures of effect:

For each primary study we will calculate the standardized mean difference (SMD, Hedges' *g*) for continuous measures (efficacy and tolerability data). If the study includes a comparator, we will use endpoint data between the active intervention vs. control intervention to calculate the SMD. If the study does not include a comparator, we will use pre vs. post data to calculate the SMD. If only change scores are reported, we will use change scores to calculate the SMD between the active intervention vs. control intervention. If both are reported, endpoint scores will be used. Dichotomous outcomes measured throughout the study and reported at as totals at endpoint (e.g., total number of self-harm episodes during the trial) will be considered endpoint data. Intention-to-treat (ITT) data will be used whenever available. Negative SMDs will indicate the active intervention was more beneficial than control intervention. The relative risk (RR) will be calculated for dichotomous tolerability data only. RR less than 1 will indicate the active intervention had less adverse effects than control intervention. Effect sizes of dichotomous efficacy data will be converted to the SMD using the procedure discussed shortly.

Combining multiple effect sizes within trials:

If a study reports multiple outcomes (efficacy or tolerability) per category, the effect sizes will be combined to produce a single composite effect size (SMD or RR) using the method in Borenstein et al (2009).[4] We will assume a within-cluster correlation between outcomes of $r = 0.45$. We will make this assumption because studies almost never report the inter-correlations between their outcome measures. $r = 0.45$ is the 95th percentile correlation across 708 meta-analytically-derived correlations in psychology between different constructs.[5] We will use the 95th percentile since it gives a more conservative estimate of the correlation between similar outcomes within studies. This is because smaller correlations lower the variance of combined effect sizes.[4]

If a continuous outcome is reported alongside its dichotomous outcome (e.g., response rate based on ≥ 50% reduction in the scale), only the continuous outcome will be extracted. This is due to the redundancy and the lower statistical power of dichotomized continuous scales. In order to combine continuous (e.g., self-report scale of impulsivity) and dichotomous (e.g., number of aggressive episodes) efficacy data within a symptom category (e.g., behavioural dyscontrol) we will perform the following procedure: an odds ratio (OR) will be calculated for the dichotomous outcome and converted to Cohen's *d* using established equations.[4] We will apply the *J* correction factor to convert the Cohen's *d* to Hedges' *g* (the SMD).[5] This will be combined with the SMD of the continuous outcome(s) within the symptom category. Note, the RR is preferred over the OR for RCTs and cohort studies. The OR approximates the RR when the incidence of the outcome is low (< 10%). As the incidence increases, the OR overestimates the RR when it is greater than 1 and underestimates the RR when it's less than 1.[6] To the best of our knowledge there are no established procedures to convert RRs directly to SMDs. Therefore, while imperfect, we will use this procedure with dichotomous outcomes given the advantages of combining multiple outcomes into a single effect size per symptom category.

Prosser A, *et al. BMJ Open* 2023; 13:e075119. doi: 10.1136/bmjopen-2023-075119

8. Data extraction (selection and coding)

1. **Selection of studies:** two reviewers will independently screen the titles and abstracts of the search results to determine potential eligibility. Full-text reports of the potentially eligible reports will then be retrieved and assessed by two reviewers using our inclusion/exclusion criteria to determine eligibility. Disagreements at either the screening or full-text review stages of the selection phase will be resolved through consensus between the two reviewers and, if necessary, a third reviewer. Decisions regarding eligibility will be documented.
2. **Data extraction:** Two reviewers will independently extract the main outcomes and additional outcomes from the reports into standardized electronic databases. Discrepancies in data extraction will be resolved through consensus between the two authors and, if necessary, a third author.

9. Risk of bias assessment

Risk of bias in the randomized controlled trials (RCTs) will be assessed using the Cochrane Risk of Bias 2 (ROB 2). Risk of bias in the non-randomized primary studies will be assessed using the Cochrane Risk of Bias in Non-randomized Studies of Interventions (ROBINS-I). Risk of bias will be assessed by two independent reviewers. All disagreements will be resolved through consensus between the two reviewers and a third reviewer if necessary. ROBINS-I requires listing in the protocol the confounding domains and co-interventions. Confounding domains and co-interventions are those for which adjustment is expected to lead to a clinically important change in the estimated effect of the intervention. We defined the following:

1. **Confounding domains:** age, sex, race/ethnicity, comorbidities, socioeconomic status.
2. **Co-interventions:** concurrent psychotherapy or pharmacotherapy.

10. Strategy for data synthesis

The characteristics of the included primary studies will be summarized in a tabular form using the PICO format along with a narrative summary of the results with the effect sizes of the efficacy/tolerability data when available. When appropriate, quality of evidence will be assessed using the GRADE approach.[7] The narrative summaries of the opinions from the non-primary studies will be summarized in tabular form.

Prosser A, *et al. BMJ Open* 2023; 13:e075119. doi: 10.1136/bmjopen-2023-075119

APPENDIX C

The following pivotal guidelines, psychotherapy manuals, book chapters, and articles will be reviewed as part of the qualitative assessment in order to extract opinions relevant to our key questions.

Guidelines

1. National Institute for Health and Clinical Excellence (NICE) guideline on treatment and management: Borderline personality disorder[8]
2. American Psychiatric Association (APA) practice guideline for the treatment of patients with borderline personality disorder[9]
3. National Health and Medical Research Council (NHMRC) clinical practice guideline for the management of borderline personality disorder[10]
4. World Federation of Societies of Biological Psychiatry (WFSBP) Guidelines for biological treatment of personality disorders[11]
5. The project BETA guidelines[12]
6. Garriga et al (2016) Assessment and management of agitation in psychiatry: Expert consensus. *The World Journal of Biological Psychiatry*.[13]

Psychotherapy manuals

1. Linehan (1993) Cognitive-behavioral treatment of borderline personality disorder.[14]
2. Bateman & Fonagy (2016) Mentalization-based treatment for personality disorders: A practical guide.[15]
3. Yeomans et al (2015) Transference focused psychotherapy for borderline personality disorder: A Clinical Guide.[16]
4. Kernberg (1984) Severe personality disorders: Psychotherapeutic Strategies.[17]
5. Gunderson & Links (2008) Borderline personality disorder: A clinical guide.[1]
6. Gunderson & Links (2014) Handbook of good psychiatric management for borderline personality disorder.[2]
7. Arntz & van Genderen (2021) Schema therapy for borderline personality disorder, 2nd Ed.[18]
8. Davidson (2008) Cognitive therapy for personality disorders: A guide for clinicians, 2nd Ed.[19]
9. Black & Blum (2016) Systems training for emotional predictability and problem solving for borderline personality disorder: Implementing STEPPS around the globe.[20]
10. Dawson & MacMillan (1993) Relationship management of the borderline patient: From understanding to treatment.[21]
11. Livesley (2003) Practical management of personality disorder.[22]
12. Livesley (2017) Integrated modular treatment for borderline personality disorder: A practical guide to combining effective treatment methods.[23]

Other books

1. Gabbard (2014) Psychodynamic psychiatry in clinical practice, 5th Ed.[24]
2. Gabbard & Wilkinson (2000) Management of countertransference with borderline patients.[25]

Prosser A, *et al. BMJ Open* 2023; 13:e075119. doi: 10.1136/bmjopen-2023-075119

3. Choi-Kain & Gunderson (2019) Applications of good psychiatric management for borderline personality disorder.[26]
4. Sonley & Choi-Kain (2020) Good psychiatric management and dialectical behavior therapy: A clinician's guide to integration and stepped care.[27]

Book chapters

1. Hong & Casher (2020) The inpatient with borderline personality disorder. In: *Manual of Inpatient Psychiatry, 2nd Ed*.[28]
2. Hong et al (2020) Personality disorders. In: *Emergency Psychiatry: Principles and Practice, 2nd Ed*.[29]
3. Hong et al (2019) Traitements dans les services d'urgence. In: *Trouble de la Personnalite Borderline: Pratiques Therapeutiques*.[30]
4. Curry & Riddle (2021) Personality disorders in the emergency department. In: *Behavioral Emergencies for Healthcare Providers*.[31]

Published papers

1. Hong (2016) Borderline personality disorder in the emergency department: good psychiatric management. *Harvard Review of Psychiatry*.[3]
2. Hong et al (2019) Antisocial and borderline personality disorders in the emergency department: conceptualizing and managing "Malingered" or "Exaggerated" symptoms. *Current Behavioral Neuroscience Reports*.[32]
3. Shaikh et al (2017). Patients with borderline personality disorder in emergency departments. *Frontiers in Psychiatry*.[33]
4. Broadbear et al (2022). Emergency department utilisation by patients with a diagnosis of borderline personality disorder: An acute response to a chronic disorder. *Emergency Medicine Australasia*.[34]
5. Cases et al (2020). Evidence of practice gaps in emergency psychiatric care for borderline personality disorder: how can this be explained? *BMC Psychiatry*.[35]
6. Acres et al (2021). From the community to the emergency department: A study of hospital emergency department nursing practices from the perspective of carers of a loved one with Borderline Personality Disorder. *Health & social care in the community*.[36]
7. Paris (2002) Chronic suicidality among patients with borderline personality disorder. *Psychiatric Services*.[37]
8. Paris (2004) Understanding self-mutilation in borderline personality disorder. *Harvard Review of Psychiatry*.[38]
9. Paris (2004) Half in love with easeful death: The meaning of chronic suicidality in borderline personality disorder. *Harvard Review of Psychiatry*.[39]
10. Paris (2004) Is hospitalization useful for suicidal patients with borderline personality disorder? *Journal of Personality Disorders*.[40]
11. Paris (2006) Predicting and preventing suicide: Do we know enough to do either? . *Harvard Review of Psychiatry*.[41]
12. Paris (2021) Can we predict or prevent suicide?: An update. *Preventative Medicine*.[42]
13. Goodman et al (2012) Suicidal risk and management in borderline personality disorder. *Current Psychiatry Reports*.[43]

Prosser A, *et al. BMJ Open* 2023; 13:e075119. doi: 10.1136/bmjopen-2023-075119

References

1. Gunderson J, Links P. Borderline Personality Disorder: A Clinical Guide. Washington, DC: American Psychiatric Association Publishing 2008.
2. Gunderson J, Links P. Handbook of Good Psychiatric Management for Borderline Personality Disorder. Washington, DC: American Psychiatric Association Publishing 2014.
3. Hong V. Borderline personality disorder in the emergency department: good psychiatric management. *Harv Rev Psychiatry* 2016;24(5):357-66. doi: 10.1097/HRP.0000000000000112
4. Borenstein M, Hedges LV, Higgins JP, et al. Introduction to Meta-analysis. Chichester, UK: Wiley 2009.
5. Gignac GE, Szodorai ET. Effect size guidelines for individual differences researchers. *Personality and individual differences* 2016;102:74-78.
6. Zhang J, Kai FY. What's the relative risk?: A method of correcting the odds ratio in cohort studies of common outcomes. *JAMA* 1998;280(19):1690-91.
7. Guyatt GH, Oxman AD, Vist GE, et al. GRADE: an emerging consensus on rating quality of evidence and strength of recommendations. *BMJ* 2008;336(7650):924. doi: 10.1136/bmj.39489.470347.AD
8. Health NCCfM. Borderline Personality Disorder: The NICE Guideline on Treatment and Management. National Clinical Practice Guideline No. 78: The British Psychological Society and The Royal College of Psychiatrists, 2009.
9. Association AP. Practice guideline for the treatment of patients with borderline personality disorder: American Psychiatric Association Practice Guidelines 2001.
10. Smith M, Chanen A, Ford F, et al. Clinical practice guideline for the management of borderline personality disorder. Melbourne: National Health and Medical Research Council (NHMRC), 2012.
11. Herpertz SC, Zanarini M, Schulz CS, et al. World Federation of Societies of Biological Psychiatry (WFSBP) guidelines for biological treatment of personality disorders. *The World Journal of Biological Psychiatry* 2007;8(4):212-44.
12. Holloman Jr GH, Zeller SL. Overview of Project BETA: best practices in evaluation and treatment of agitation. *Western Journal of Emergency Medicine* 2012;13(1):1.
13. Garriga M, Pacchiarotti I, Kasper S, et al. Assessment and management of agitation in psychiatry: Expert consensus. *The World Journal of Biological Psychiatry* 2016;17(2):86-128. doi: 10.3109/15622975.2015.1132007
14. Linehan M. Cognitive-behavioral Treatment of Borderline Personality Disorder. New York City, NY: Guilford Press 1993.
15. Bateman A, Fonagy P. Mentalization based treatment for personality disorders: A practical guide. Oxford, UK: Oxford University Press 2016.
16. Yeomans FE, Clarkin JF, Kernberg OF. Transference-Focused Psychotherapy for Borderline Personality Disorder: A Clinical Guide. Arlington, VA: American Psychiatric Association Publishing 2015.
17. Kernberg OF. Severe Personality Disorders: Psychotherapeutic Strategies. New Haven, CT: Yale University Press 1984.
18. Arntz A, Van Genderen H. Schema therapy for borderline personality disorder. West Sussex, UK: John Wiley & Sons, Ltd 2021.
19. Davidson K. Cognitive therapy for personality disorders: A guide for clinicians. 2nd ed. East Sussex, UK: Routledge 2008.

20. Black DW, Blum N. Systems training for emotional predictability and problem solving for borderline personality disorder: Implementing STEPPS around the globe. New York City, NY: Oxford University Press 2017.
21. Dawson DL, MacMillan HL. Relationship management of the borderline patient: from understanding to treatment. New York City, NY: Routledge 1993.
22. Livesley J. Practical Management of Personality Disorder. New York City, NY: Guilford Press 2003.
23. Livesley J. Integrated Treatment for Borderline Personality Disorder: A Practical Guide to Combining Effective Treatment Methods. Cambridge, UK: Cambridge University Press 2017.
24. Gabbard GO. Psychodynamic psychiatry in clinical practice. 5th ed. Arlington, VA: American Psychiatric Association Publishing 2014.
25. Gabbard GO, Wilkinson SM. Management of countertransference with borderline patients. Northvale, New Jersey: Jason Aronson Inc. 2000.
26. Choi-Kain LW, Gunderson JG. Applications of good psychiatric management for borderline personality disorder: a practical guide. Washington, DC: American Psychiatric Association Publishing 2019.
27. Sonley AK, Choi-Kain LW. Good psychiatric management and dialectical behavior therapy: A clinician's guide to integration and stepped care. Washington, DC: American Psychiatric Association Publishing 2021.
28. Hong V, Casher M. The inpatient with borderline personality disorder. In: Casher M, Bess J, eds. Manual of Inpatient Psychiatry. 2nd ed. Cambridge, UK: Cambridge University Press 2020.
29. Hong V, Reddy P, Entenman S. Personality Disorders. In: Glick R, Zeller S, Berlin J, eds. Emergency Psychiatry: Principles and Practice. 2nd ed. Philadelphia, PA: Wolters Kluwer 2020.
30. Hong V, Kozada K, Shobassy A. Traitements dans les services d’urgence. In: Kolly S, Charbon P, Kramer U, eds. Trouble de la Personnalite Borderline: Pratiques Therapeutiques France: Elsevier Masson SAS 2019.
31. Curry A, Riddle M. Personality Disorders in the Emergency Department. In: Zun L, Nordstrom K, Wilson M, eds. Behavioral Emergencies for Healthcare Providers. 2nd ed. Switzerland: Springer Nature 2021.
32. Hong V, Pirnie L, Shobassy A. Antisocial and borderline personality disorders in the emergency department: conceptualizing and managing “Malingered” or “Exaggerated” symptoms. *Current Behavioral Neuroscience Reports* 2019;6(4):127-32.
33. Shaikh U, Qamar I, Jafry F, et al. Patients with borderline personality disorder in emergency departments. *Frontiers in psychiatry* 2017;8:136.
34. Broadbear JH, Rotella J-A, Lorenze D, et al. Emergency department utilisation by patients with a diagnosis of borderline personality disorder: An acute response to a chronic disorder. *Emerg Med Australas* 2022;34(5):731-37.
35. Cases C, Lafont Rapnouil S, Gallini A, et al. Evidence of practice gaps in emergency psychiatric care for borderline personality disorder: how can this be explained? *BMC Psychiatry* 2020;20(1):476. doi: 10.1186/s12888-020-02892-7
36. Acres K, Loughhead M, Procter N. From the community to the emergency department: A study of hospital emergency department nursing practices from the perspective of carers

of a loved one with Borderline Personality Disorder. *Health Soc Care Community* 2022;30(5):1789-97.
37. Paris J. Chronic Suicidality Among Patients With Borderline Personality Disorder. *Psychiatr Serv* 2002;53(6):738-42. doi: 10.1176/appi.ps.53.6.738
38. Paris J. Understanding Self-mutilation in Borderline Personality Disorder. *Harv Rev Psychiatry* 2005;13(3):179-85. doi: 10.1080/10673220591003614
39. Paris J. Half in Love with Easeful Death: The Meaning of Chronic Suicidality in Borderline Personality Disorder. *Harv Rev Psychiatry* 2004;12(1):42-48. doi: 10.1080/10673220490279161
40. Paris J. Is Hospitalization Useful for Suicidal Patients with Borderline Personality Disorder? *J Personal Disord* 2004;18(3):240-47. doi: 10.1521/pedi.18.3.240.35443
41. Paris J. Predicting and Preventing Suicide: Do We Know Enough to Do Either? *Harv Rev Psychiatry* 2006;14(5):233-40. doi: 10.1080/10673220600968662
42. Paris J. Can we predict or prevent suicide?: An update. *Prev Med* 2021;152(Pt 1):106353. doi: 10.1016/j.ypmed.2020.106353 [published Online First: 2021/09/21]
43. Goodman M, Roiff T, Oakes AH, et al. Suicidal Risk and Management in Borderline Personality Disorder. *Curr Psychiatry Rep* 2012;14(1):79-85. doi: 10.1007/s11920-011-0249-4
