# Supplementary material for: Delphi consensus study to develop guidelines for the management of adults with borderline personality disorder in the emergency department: a protocol
Source: BMJ Open. 2023 Nov 9;13(11):e075119. doi: 10.1136/bmjopen-2023-075119 (PMC10649594; doi:10.1136/bmjopen-2023-075119)
Supplement: Author's manuscript [file bmjopen-2023-075119.draft_revisions.pdf]

BMJ Open is committed to open peer review. As part of this commitment we make the peer review history of every article we publish publicly available.

When an article is published we post the peer reviewers' comments and the authors' responses online. We also post the versions of the paper that were used during peer review. These are the versions that the peer review comments apply to.

The versions of the paper that follow are the versions that were submitted during the peer review process. They are not the versions of record or the final published versions. They should not be cited or distributed as the published version of this manuscript.

BMJ Open is an open access journal and the full, final, typeset and author-corrected version of record of the manuscript is available on our site with no access controls, subscription charges or pay-per-view fees (http://bmjopen.bmj.com).

If you have any questions on BMJ Open's open peer review process please email info.bmjopen@bmj.com

BMJ Open

Protocol for a Delphi consensus study to develop guidelines for the management of borderline personality disorder in the emergency department

Journal:	*BMJ Open*
Manuscript ID	bmjopen-2023-075119
Article Type:	Protocol
Date Submitted by the Author:	25-May-2023

Complete List of Authors:	Prosser, Aaron; Hamilton, Department of Psychiatry and Behavioural Neurosciences<br>Hong, Victor; University of Michigan, Department of Psychiatry<br>Helfer, Bartosz; University of Wroclaw, Institute of Psychology; University of Wroclaw, Meta-Research Centre<br>Fudge, David; Hamilton, Department of Psychiatry and Behavioural Neurosciences<br>Patterson, Janet; Hamilton, Department of Psychiatry and Behavioural Neurosciences<br>Rosebush, Patricia; Hamilton, Department of Psychiatry and Behavioural Neurosciences<br>Frey, Benicio; McMaster University, McMaster Integrative Neuroscience Discovery and Study (MiNDS); St. Joseph’s Healthcare Hamilton, Mood Disorders Program<br>Links, Paul; Hamilton, Department of Psychiatry and Behavioural Neurosciences

Keywords:	PSYCHIATRY, ACCIDENT & EMERGENCY MEDICINE, Personality disorders < PSYCHIATRY, Suicide & self-harm < PSYCHIATRY

SCHOLARONE™
Manuscripts

Protocol for a Delphi consensus study to develop guidelines for the management of borderline personality disorder in the emergency department

Aaron Prosser MD MSc[1]
Victor Hong MD[2]
Bartosz Helfer PhD MSc[3,4]
David Fudge MD[1]
Janet Patterson MD[1]
Patricia Rosebush MD[1]
Benicio Frey MD PhD[1]
Paul Links MD MSc[1]

[1]Department of Psychiatry and Behavioural Neurosciences, McMaster University, Hamilton, Ontario, Canada
[2]Department of Psychiatry, University of Michigan, Ann Arbor, MI, United States
[3]Institute of Psychology, University of Wroclaw, Wroclaw, Poland
[4]Meta-Research Centre, University of Wroclaw, Wroclaw, Poland

Corresponding author: Aaron Prosser (aaron.prosser@medportal.ca)

Word count: 4,487

For peer review only

ABSTRACT

Introduction: Clinicians caring for people with borderline personality disorder (BPD) in acute settings such as the emergency department (ED) have little evidence/guidance to base decisions on. Specific/detailed guidance for managing BPD in the ED is needed given the morbidity and mortality, high service utilization, unique challenges, and risk of iatrogenic interventions. The primary objective of this study is to use a consensus method to develop a guideline for managing BPD in the ED. This protocol and the key questions for the guideline were developed with the advice of people with BPD and their family members/support persons.

Methods and analysis: We will perform a 4-phase Delphi study of an expert panel of clinicians, researchers, people with BPD, and their family members/support persons. Various disciplines (psychiatry, psychology, emergency medicine, nursing, social work) and treatment approaches will be included in the expert panel. An online questionnaire will be developed from systematic reviews, qualitative assessments of pivotal literature, and opinions suggested by the panel (Phase 1). The panel will rate their agreement on opinions for each key question covering areas of emergency care of people with BPD using two rounds of this questionnaire (Phases 2 and 3). Opinions meeting pre-defined thresholds for consensus will be brought to consensus meetings moderated by an independent chair (Phase 4). The purpose of these meetings is to finalise the set and phrasing of the opinions for each area of emergency care. These final opinions will be the recommendations in the guideline. If there are significant differences of opinion, the guideline will present both recommendations alongside one another.

Ethics and dissemination: The Hamilton Integrated Research Ethics Board in Hamilton, Ontario, Canada, is currently reviewing an ethics application. The results of this study will be disseminated through peer-reviewed publications, conferences, and national professional and patient/family/support associations.

For peer review only

For peer review only - http://bmjopen.bmj.com/site/about/guidelines.xhtml

STRENGTHS AND LIMITATIONS OF THIS STUDY

- The primary objective of this study is to develop a guideline for managing BPD in the ED. This protocol and the key questions for the guideline were developed with the advice of people with BPD and their family members/support persons.
- The Delphi method will be used due to the limited research in this area to inform a guideline.
- Our methods use a questionnaire to minimise the disadvantages of group discussion (e.g., domination by individuals or pressure to conform) while the consensus meeting capitalises on its advantages (e.g., clarifying ambiguities or editing the phrasing of recommendations).
- The expert panel will include participants from different disciplines, backgrounds, and treatment approaches including people with BPD, their family members and support persons, clinicians, and researchers.
- The expert panel might not be fully representative since recruitment is not based on random sampling and the recommendations will be based on consensus, which is based on shared knowledge and practical experience rather than scientific proof.

For peer review only

For peer review only - http://bmjopen.bmj.com/site/about/guidelines.xhtml

INTRODUCTION

The need for guidelines

Borderline personality disorder (BPD) is a highly heritable (~55%) condition whose core pathology is a disturbance in identity, attachment, and emotion regulation.[1 2] BPD is often viewed as a trauma disorder since for many it emerges from psychosocial adversity interacting with a genetic diathesis.[1 2] The symptomatic expression of BPD includes dysphoria, dysregulation, recurrent self-harm/suicide attempts, and conflicted/unstable relationships.[3] These symptoms are often severe and disabling, causing frequent crises and presentations to the emergency department (ED), with frequent suicidal thoughts and self-harm behaviors being behavioral trademarks of the disorder. Specialized outpatient treatments for BPD have been developed and shown to be effective in randomised controlled trials (RCTs).[4] There is also limited RCT evidence for some medications targeting specific clusters of symptoms (e.g., emotion dysregulation, impulsivity).[5 6] These developments are a major accomplishment and have informed guidelines on BPD, notably the NICE,[7] APA,[8] and NHMRC[9] guidelines.

While crises and acute treatment are addressed in these guidelines, the sections are brief since most of the evidence pertains to outpatient settings. Therefore, we formed a Guideline Working Group (GWG) to oversee the development of a guideline on managing BPD in the ED. Our focus is on adults with BPD, although certainly many of the principles may apply to adolescents. Our primary aim will be to identify a set of recommendations for each key question (KQ) using Delphi consensus methods. Each KQ defines a key area of the emergency care of people with BPD. Specific and detailed guidance for managing BPD in the ED is needed given the common pitfalls related to high service utilization, unique challenges in the emergency setting, risk of iatrogenic interventions, and recurrent suicide attempts and self-harm.

High service utilization and unique challenges

People with BPD are frequent visitors to the ED, representing upwards of 9% of all visits in some jurisdictions, with recurrent visits being common.[10] Reasons for presenting to the ED are numerous, including self-harm, suicide attempts, situational crises, adjustment reactions, depression, anxiety, emotion dysregulation, agitation, intoxication, withdrawal, substance induced psychosis, and housing/financial problems. There are patient, staff, and systems factors which create unique challenges to managing BPD in ED settings.[11] Some are listed in Table 1.

Table 1. Challenges to managing BPD in ED settings

Patient	Staff	System
• Recurrent ED visits and high service utilization.<br>• Disruptive behaviours.<br>• Chronic high risk for suicide and self-harm.<br>• Transference reactions.	• Problematic staff attitudes.<br>• Varying levels of training.<br>• Ill-defined/time-limited responsibility for patients.<br>• Countertransference reactions.	• Transient/changing ED staff.<br>• Changing ED workloads.<br>• Disposition problems.<br>• Availability of inpatient beds, aftercare services, and rapid outpatient follow-up.

High risk of iatrogenic interventions in the emergency department

For peer review only - http://bmjopen.bmj.com/site/about/guidelines.xhtml

People with BPD appear to be at higher risk of iatrogenic interventions in all clinical settings, particularly acute settings like the ED.[11] These include inadequate risk assessment, unnecessary inpatient hospitalisations, excessive use of medications, or unnecessary use of seclusion or physical/chemical restraints. Staff attitudes and countertransference reactions can also drive iatrogenesis. Staff who are otherwise empathic, validating, supportive, and effective clinicians may have negative or positive reactions which – if not examined, accepted, and contained – can be the driver of harmful interventions.[12] Negative reactions include withdrawal, rejection, hostility, helplessness, hopelessness, anxiety, or feeling overwhelmed. Positive yet often unhelpful reactions include rescue fantasies or feeling excessively protective of or responsible for a patient. Lack of awareness of the separation of acute and chronic risk factors may play a role in these reactions. Iatrogenesis is likely caused by an interplay between these patient, staff, and systems factors, often amplified in the ED.

Risk of recurrent suicide attempts and self-harm

Recurrent suicidal acts, threats, urges and self-harm behaviours are defining symptoms of BPD.[3] The risk of suicide is 3%-10% in people with BPD and the average number of suicide attempts per individual is 3, with completed suicide occurring about once per 23 attempts.[1] About 75% of people with BPD self-harm and among these 90% repeatedly self-harm.[1] Taking on this risk is inherent to the work of caring for people with BPD. These risks are greatest for clinicians working in the ED because people with BPD presenting to the ED often do so because they are acutely dysphoric, actively suicidal, engaged in severe self-harm, and/or attempted to end their life. However, discerning the difference between acute vs. chronic suicide/self-harm risk is difficult when a patient is chronically at high risk. This is especially the case when the risk assessment/management must be done by a clinician who doesn't have a longitudinal relationship with the patient, which is the reality for most clinicians working in the ED. For these reasons, many clinicians experience fear not only for the safety of the people with BPD they care for, but also their own medico-legal liability. This is yet another reason why specific and detailed guidance for managing BPD in the ED is needed, especially regarding suicide/self-harm risk assessment/management and liability management.

METHODS AND ANALYSIS

Rationale for the Delphi method

Our project started by first determining the most appropriate method to develop the guideline. Guidelines are usually constructed from systematic reviews and meta-analyses of primary studies. However, our group was already aware that there is scant research in this area. This was confirmed by pilot systematic searches. Therefore, we decided to conduct a Delphi consensus study to come up with recommendations from clinicians, researchers, patients, and family members/support persons.

Development of key questions

Our KQs were built from the two main tasks which need to be addressed for all people with BPD who come to the ED. We used the analytic-graphical model as developed by Nelson et

For peer review only - http://bmjopen.bmj.com/site/about/guidelines.xhtml

al. (2010)[13] to visualize the analytic framework and our KQs (Figure 1). The first is a risk assessment (KQ2). This usually starts with the emergency medicine team who may or may not ask the psychiatrist and/or mental health clinicians (e.g., psychiatric nurses, social workers) for further assistance in the assessment and disposition planning. The second is to decide if to admit (KQ3) or discharge (KQ4) the person. Alongside this process is the overall care of the person with BPD while they are in the ED (KQ1). Here we mean the *general interventions* to use with all patients throughout their stay in the ED (e.g., empathy, validation, management of countertransference and bias) and *specific interventions* (e.g., psychoeducation, medications) to address the specific needs of a patient. A related key area are interventions for the management of agitation and aggression in people with BPD in the ED (KQ5). Another key area is the involvement and role of family members and support persons (KQ6) and outpatient providers (KQ7) while people with BPD are in the ED. The last key areas are approaches to providing optimal medical care (KQ8) and effective environmental elements (KQ9) for people with BPD presenting to the ED in a mental health crisis.

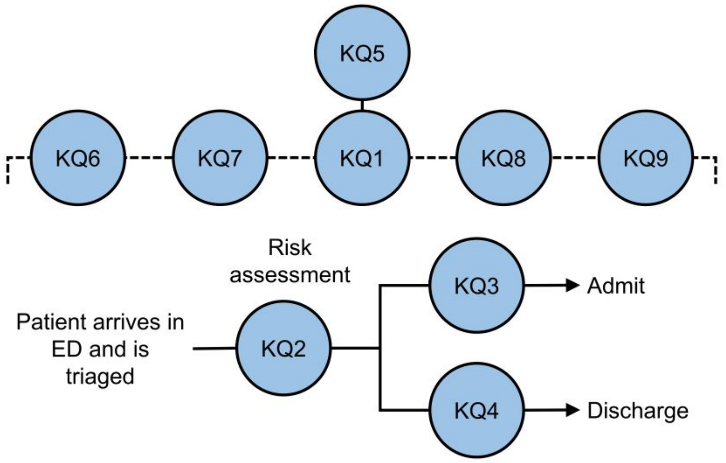

Figure 1. Analytic framework and key questions (KQs).

Based on this analytic framework, we constructed seven KQs:

1. What interventions in the ED are associated with benefit while minimising harm for people with BPD?
    a. What interventions in the ED are effective?
    b. What interventions in the ED are harmful?
2. What are the effective approaches to assess and manage the risk of suicide and self-harm of people with BPD?
3. What is the role of hospitalisation in managing people with BPD?
    a. What are the indications for hospitalisation?
    b. What is the effectiveness of hospitalisation?
    c. What are the harms of hospitalisation?
4. What aftercare services can help people with BPD who present to the ED but are not hospitalised?
5. What interventions in the ED for agitation and aggression are associated with benefit while minimising harm for people with BPD?
    a. What interventions for agitation and aggression are effective?

For peer review only - http://bmjopen.bmj.com/site/about/guidelines.xhtml

b. What interventions for agitation and aggression are harmful?

6. What are effective approaches to involving family members and support persons in the care of people with BPD in the ED?
7. What are effective approaches to involving outpatient providers in the care of people with BPD in the ED?
8. What are effective approaches to providing optimal medical care for people with BPD presenting in a mental health crisis to the ED ?
9. What are the most effective environmental elements for people with BPD in the ED?

We will systematically review the literature on each KQ, the results of which will be integrated into the Delphi process (see methods section for details). The Delphi process will be used to provide guidance on each of these KQs. It will draw on the expertise of people across disciplines (psychiatry, psychology, emergency medicine, nursing, social work), backgrounds (clinicians, researchers, patients, family, support persons), and treatment approaches. The latter will include dialectical behavior therapy (DBT), transference-focused psychotherapy (TFP), mentalization-based treatment (MBT), good psychiatric management (GPM), schema focused therapy (SFT), cognitive behavior therapy (CBT), systems training for emotional predictability and problem solving (STEPPS), integrated modular treatment (IMT), and psychopharmacology. Other content experts who may not align with a specific treatment approach will be contacted for participation. The participant recruitment plan is described below.

Engagement of persons with BPD and their family members/support persons in development of key questions

While developing this protocol and the KQs included here, we sought advice from people with BPD and their family members/support persons on what are the most important questions to include from their perspective as well as feedback on the earlier draft of the protocol. VH contacted potential participants by utilizing the listserve from several groups including the National Alliance on Mental Illness (NAMI) and the National Education Alliance for Borderline Personality Disorder (NEA-BPD), and 13 provided constructive feedback including 3 persons with BPD and 9 family members and 1 support person of someone with BPD. Participants were asked to review the KQs, provide feedback, and propose other KQs. All 13 participants were those who themselves or had supported a person who had experience with an emergency department presentation for BPD-related issues. They agreed on the set of proposed KQs and 4 participants included specific suggestions. One person with BPD wanted KQ7 to be explored further. Family members and the support person requested additional KQs and edits, all of them were applied after consultation with the GWG and are listed here: (1) two additional KQ (8 and 9) were added, (2) the order of questions was changed (KQ4 became KQ1 and the numbering of the others was adjusted accordingly), (3) "support person" was added to KQ6 and the word "aggression" to KQ5. We have also edited the methods of the guideline development process below to include the wider support circle of people with BPD (going beyond family members or caregivers). 10 others expressed interest in participating in this phase of the project. 1 did not meet criteria because they did not have BPD, 1 ultimately did not choose to participate, and 8 did not respond to the protocol when it was shared. Additional feedback included preferences relating to the need to consider biases based on social-economic-status, race and gender as well during the guideline development process. Their preferences will be included in the Delphi

For peer review only - http://bmjopen.bmj.com/site/about/guidelines.xhtml

process and shared with the guideline development team. Moreover, special attention will be given to these biases while preparing the systematic review.

Study design

This study will use the Delphi method in order to achieve consensus across the group of participants. This method is appropriate for our purposes because it maximises the benefits of group discussion while minimising its disadvantages.[14] The main disadvantages are domination by powerful individuals, coalitions, vested interests, the biasing effects of personality and seniority, that only one person can speak at a time, and group pressures for conformity. The Delphi method provides a structured, democratic, transparent, and iterative procedure with controlled feedback to participants to determine the extent of agreement amongst a group about a given issue.[14,15] The procedure will consist of questionnaire development (Phase 1), rating evaluation (Phase 2), re-rating (Phase 3), and consensus meeting (Phase 4). Phase 1 will include systematic reviews of our KQs to incorporate any recommendations which may come from the existing literature.

Study governance

The GWG is comprised of five psychiatrists (VH, DF, PR, JP, PL), a psychiatry resident (AP), and a meta-researcher (BH) with expertise in guideline development. All clinicians in the GWG currently treat people with BPD in outpatient, inpatient, and emergency settings. One of the members (PL) was a co-developer of GPM[1,16] and another (VH) published an expert opinion on the management of BPD in the ED based on GPM principles.[11] As a result, PL and VH will also be participants given their recognized expertise. However, this also is a potential source of bias. We detail below how we will mitigate this. Study methods for the protocol were established in virtual meetings and email correspondence. The GWG will meet at minimum once quarterly and at higher frequency as needed until projected completion. Meetings will be held virtually and we will have regular email correspondence. To further address any outstanding issues with study governance, we will follow the complete interaction approach with full transparency between the systematic review team and eventual guideline developers.[17]

Involvement of persons with BPD and their families/support persons

People with BPD, their family members and support persons will have a voice in these guidelines since the recommendations will directly impact them. We recognize that there may be differences of opinion between patient/family/support persons and clinician/researcher on some questions during the Delphi process. If there are significant differences in opinion, we will be transparent about these differences in the guideline. This ensures that patient/family/support persons voices are not silenced by majority opinion since we anticipate there will be more clinician/researcher participants. Patients or patients' representatives will be continuously involved throughout the guideline development process following a modified model by Armstrong et al. (2010).[18] Following NICE recommendations, at least at least two patient/caregiver/advocate members will be involved in the Delphi process and consensus meeting as panelists.[19]

For peer review only - http://bmjopen.bmj.com/site/about/guidelines.xhtml

Participant selection/recruitment

Participants will be included if they are clinicians and/or researchers who have significant experience in the management of BPD in the ED. We will also recruit patients and family members/support persons. The clinician/researcher sample will be recruited using a number of strategies. First, we will contact national professional organizations for emergency medicine, psychiatry, nursing, and social work in English-speaking countries, specifically the United States, Canada, United Kingdom, Ireland, South Africa, Australia, and New Zealand. We will ask them to advertise our study through their email mailing lists. Second, the GWG is already aware of a number of experts who we will invite to participate. These individuals were either a developer or published expert in a treatment approach for BPD, a clinician/group member on the NICE, APA, and NHMRC guidelines, or is a published expert in the management of BPD in the ED. Third, the publication of this protocol will also help advertise this study. Patients and family members/support persons will be recruited by contacting national patient advocacy and support organizations. We will ask these organizations to advertise our study through their email mailing lists and social media to help with recruitment. We will ask all participants to provide suggestions too. Readers of this protocol may also give suggestions. Suggestions can be made to the GWG by contacting the corresponding author of this protocol (aaron.prosser@medportal.ca). The deadline for suggesting a participant is 3 months after the publication date of this protocol.

Delphi procedure

The Delphi process is an iterative procedure which typically proceeds in three phases.[15] We will add a fourth phase in order to have a consensus meeting moderated by an independent chair, thus combining the Delphi method with the consensus conference method. This is because we believe that an open-ended group discussion has advantages over questionnaires. In particular, group discussion can be helpful for clarifying ambiguities and differences and editing phrasing for the final recommendations of the guideline. Furthermore, a meeting provides an opportunity to reach consensus on issues where consensus has not yet been achieved.

Phase 1 – Questionnaire development

Phase 1 has three goals: (1) create the expert panel, (2) create a pool of opinions on each KQ from three sources of information, and (3) create an online questionnaire using this pool of opinions for rating evaluation by the expert panel. The expert panel will be created using the selection and recruitment strategies described above.

The first source of information we will use for generating the opinion pool will be systematic reviews of the literature on each KQ. Full details of our methods, including search syntax, are in Appendix A of the supplementary materials. Summary opinions will be generated by the GWG based on the results of these systematic reviews.

The second source of information is a qualitative assessment of pivotal guidelines, psychotherapy manuals, book chapters, and articles (Appendix B of the supplementary materials). This allows us to include pivotal literature which will not necessarily be identified in the systematic reviews (e.g., psychotherapy manuals). AP will search these sources to extract a narrative summary of the opinions on each KQ. Quality control will be confirmed by an independent assessor on a random 20% subset of the opinions from these pivotal sources.

For peer review only - http://bmjopen.bmj.com/site/about/guidelines.xhtml

The third source of information will be our expert panel. After the expert panel has been created, we will invite them to provide written opinions on each KQ. They will be given the results of the systematic reviews and qualitative assessment in tabular form to review before providing their opinions.

Any evidence synthesis components employed in the process of questionnaire development will follow the relevant recommendations of the Institute of Medicine's Committee on Standards for Systematic Reviews of Comparative Effectiveness Research, especially regarding the qualitative assessment.[20]

We anticipate there will be nearly identical opinions for some KQs across the three sources of information. This will lead to significant redundancy which will unnecessarily burden the participants with repeating ratings on nearly identical opinions. For this reason, two independent assessors will thematically group the identical opinions and meet for consensus. After consensus on the groupings is achieved, they will then draft a single summary opinion. This pool of non-redundant opinions will be grouped together under their respective KQs and prepared for circulation to all participants in the form of an online questionnaire. The sources of the opinions will be masked to all participants to eliminate bias (e.g., allegiance to a treatment approach).

Phase 2 – Rating evaluation

Participants will be emailed the questionnaire and asked to rate each opinion individually on a 5-point Likert scale of agreement: 1 = strongly disagree, 2 = disagree, 3 = neutral, 4 = agree, and 5 = strongly agree. The responses will be de-identified and the opinions will be grouped into their respective KQs. Once all the questionnaires have been received, the ratings will be statistically summarized for each opinion. This will involve calculating and graphically depicting the median, inter-quartile range (IQR), and the percentages in each response category for each opinion, separately for patient/family/support persons and clinician/researcher groups. This statistical summary will be given as feedback to all participants, along with their own rating, so they can see where their ratings are relative to others for each opinion under each KQ.

Phase 3 – Re-rating

Participants will be emailed the same questionnaire and asked to re-rate their agreement on each opinion. This gives participants the opportunity to change their ratings in light of the feedback. After all the questionnaires have been received, the ratings will be statistically analysed using pre-defined consensus thresholds (Table 2).

Opinions meeting threshold for essential, recommended, or no consensus will be brought forward to the consensus meeting for review. If at least one opinion meets one of these thresholds, then we will organize a virtual video meeting. Opinions meeting threshold for exclusion will not be brought to the consensus meeting. If no opinions can be brought to the consensus meeting, then a third round of ratings will be performed. If no opinions can be brought forward after a third round, then the consensus meeting will be held on all the opinions.

Table 2. Consensus thresholds

Essential	The first quartile (Q1) of the ratings for the opinion is > 3 (neutral) in *both* the patient/family/support persons *and* clinician/researcher groups.

For peer review only - http://bmjopen.bmj.com/site/about/guidelines.xhtml

Recommended	The first quartile (Q1) of the ratings for the opinion is > 3 (neutral) in *either* the patient/family/support persons *or* clinician/researcher groups.
Exclusion	The third quartile (Q3) of the ratings for the opinion is < 2 (disagree) in *both* the patient/family/support persons *and* clinician/researcher groups.
No consensus	Essential, recommended, or exclusion thresholds have not been met.

Phase 4 – Moderated consensus meeting

The purpose of this meeting is to finalise the set of opinions and phrasing for each KQ. We will have an independent chair for this meeting to minimise the disadvantages of group discussion by moderating the discussion. The chair will also help identify when significant differences of opinion between patient/family/support persons vs. clinician/researcher exist. Multiple meetings may take place at the discretion of the chair. The meetings will be recorded for reference if necessary and securely stored. However, all opinions will be considered final at the end of this consensus meeting process. Email correspondence will be allowed during Phase 4 amongst the chair and participants in order to edit phrasing of opinions.

Guideline development

The final opinions from Phase 4 will be the recommendations in the guideline, which will be written up by the GWG. No changes to the opinions will be made by the GWG. If there are significant differences of opinion, the guideline will present the differing recommendations alongside one another.

Risk of bias mitigation

PL and VH will be participants in the Delphi process given their recognized expertise. This means there is a potential source of bias due to their involvement in developing GPM[1,16] and GPM applications to ED settings.[11] This will be mitigated by ensuring they have no access to the data or involvement in the data analysis. The chair of the consensus meeting will have no affiliation with a treatment approach.

Conflict of interest management

All members participating in the GWG as well as in the Delphi process will submit a conflict of interest (COI) disclosure form, developed based on the International Committee of Medical Journal Editors (ICMJE) form. Any reported COI will be documented and discussed by the GWG in light of its potential impact on the proposed recommendations. Participants with any declared COI will represent no more than a minority of all study participants. The chair of the consensus meeting will have no COI.

Statistical analysis

We will analyse the ratings using medians, IQRs, and percentages for each response category. These values will also be graphically depicted in the feedback provided to the participants. We will statistically compare the agreement ratings between patient/family/support

For peer review only - http://bmjopen.bmj.com/site/about/guidelines.xhtml
For peer review only

persons vs. clinician/researcher groups using independent samples *t*-tests or Wilcoxon rank-sum tests depending on if parametric assumptions are met or not, respectively, and document these differences in the final version of the guideline. All tests of significance will be two-tailed with α set at 0.05.

ETHICS

The Hamilton Integrated Research Ethics Board in Hamilton, Ontario, Canada, is currently reviewing an ethics application for this study. Potential participants will be informed that responding to the online questionnaire implies consent to participate in the study. Prior to responding, they will be informed of the purpose of the study, that their ratings will be de-identified, and their de-identified responses will be included in feedback and analyses. The consensus meeting chair will also clearly explain the purpose of the meeting and obtain written informed consent beforehand. All data and the recording of the consensus meeting will be securely stored in a locked office. Only the GWG, except PL and VH, will have access to the data and it will be destroyed after 10 years.

DISSEMINATION PLAN

We anticipate that the guideline emerging from this Delphi study will be published in a high-impact peer-reviewed journal. The guideline will be disseminated to regional, national, and international audiences through presentations at regional, national, and international conferences. Finally, we will disseminate the guideline through the national and international associations for psychiatry, emergency medicine, nursing, and social work.

DISCUSSION

Clinicians caring for people with BPD in acute settings such as the ED have little guidance or evidence to base their decisions on. In this vacuum, patients are at risk of a wide variety of treatment approaches and practices, ranging from excellent to iatrogenic. Therefore, specific and detailed guidance for managing BPD in the ED is urgently needed for the sake of patients and their families. Consensus methods such as Delphi play an important role in science where there is limited or contradictory evidence.[15] Through a structured process, these methods assess the degree of agreement and/or resolve disagreement amongst experts. The consensus can provide helpful scaffolding for clinical guidelines until empirical study fills the gaps. Furthermore, the clinical guidelines will provide important hypotheses for these empirical investigations. This is the rationale for our Delphi study. The opinions emerging from this study will become the first guideline on the management of BPD in the ED.

The nominal group technique is another consensus method used in health research.[15] It uses a facilitated meeting of experts who gather information, rate, discuss, and re-rate/discuss a series of items, questions, or opinions. Each participant contributes their ideas to the facilitator who thematically groups the ideas for discussion. The ideas are then rated, summarized, and the summary is fed back to the participants for discussion and re-rating. The final ratings are then summarized and evaluated for consensus using pre-defined thresholds. The disadvantage of this technique is that it is often not feasible for all relevant experts to participate in the meeting. This is why typically the nominal group technique usually involves small groups of 9-12

For peer review only - http://bmjopen.bmj.com/site/about/guidelines.xhtml

individuals.[15] Moreover, experts often do not have the time to devote to these steps commensurate with the complexity of the issues. By contrast, the Delphi method allows researchers to synthesize a large amount of information across many individuals with relatively minimal imposition on the participants' time.

The main limitation of this study is that, even if we achieve consensus on some or all opinions, this does not guarantee that the correct recommendations have been found. Consensus does not necessarily imply truth. Only empirical studies can validate the opinions generated from a consensus process, which at this time are lacking. Therefore, our study is best viewed as a structured sampling of expert opinions rather than an attempt to reach a definitive conclusion about what are truly the best practices in managing BPD in the ED. Another limitation is that our expert panel will not be representative of the disciplines, backgrounds, and treatment approaches we are sampling. Relatedly, we anticipate there will be more clinician/researcher participants on the panel, which risks silencing patient/family/support persons voices. This risk will be mitigated by documenting and discussing patient/family/support persons recommendations if significant differences of opinion exist. Notwithstanding these limitations, we hope the guidance emerging from this Delphi study will be a helpful clinical resource to improve the care of people with BPD in the ED.

For peer review only

For peer review only - http://bmjopen.bmj.com/site/about/guidelines.xhtml

Contributors
All authors contributed equally to the conception of this study. AP, VH, BH, and PL contributed equally to the design of this study. AP wrote the first draft of the protocol. All authors gave critical feedback on the revised protocol and approved the final version of the manuscript. All authors provided final approval for publication and agree to be accountable for all aspects of this work. The corresponding author attests all listed authors meet authorship criteria and that no others meeting criteria have been omitted.

Funding
None.

Competing interests
PL is a co-developer of GPM and VH published an expert opinion on the management of BPD in the ED based on GPM principles.

Acknowledgement
We would like to express our gratitude to the people with BPD and their family members/support persons for reviewing and commenting on an earlier draft of this protocol, as well as providing advice on the key questions for the guideline.

For peer review only

For peer review only - http://bmjopen.bmj.com/site/about/guidelines.xhtml

REFERENCES

1. Gunderson J, Links P. Handbook of Good Psychiatric Management for Borderline Personality Disorder. Washington, DC: American Psychiatric Association Publishing 2014.
2. Livesley J. Integrated Treatment for Borderline Personality Disorder: A Practical Guide to Combining Effective Treatment Methods. Cambridge, UK: Cambridge University Press 2017.
3. APA. Diagnostic and Statistical Manual of Mental Disorders. 5th ed. Washington, DC: Author. 2013.
4. Cristea IA, Gentili C, Cotet CD, et al. Efficacy of Psychotherapies for Borderline Personality Disorder: A Systematic Review and Meta-analysis. *JAMA psychiatry* 2017;74(4):319-28. doi: 10.1001/jamapsychiatry.2016.4287
5. Lieb K, Völlm B, Rücker G, et al. Pharmacotherapy for borderline personality disorder: Cochrane systematic review of randomised trials. *The British Journal of Psychiatry* 2010;196(1):4-12. doi: 10.1192/bjp.bp.108.062984
6. Stoffers-Winterling J, Storebø OJ, Lieb K. Pharmacotherapy for borderline personality disorder: an update of published, unpublished and ongoing studies. *Curr Psychiatry Rep* 2020;22:1-10. doi: 10.1007/s11920-020-01164-1
7. Health NCCfM. Borderline Personality Disorder: The NICE Guideline on Treatment and Management. National Clinical Practice Guideline No. 78: The British Psychological Society and The Royal College of Psychiatrists, 2009.
8. Association AP. Practice guideline for the treatment of patients with borderline personality disorder: American Psychiatric Association Practice Guidelines 2001.
9. Smith M, Chanen A, Ford F, et al. Clinical practice guideline for the management of borderline personality disorder. Melbourne: National Health and Medical Research Council (NHMRC), 2012.
10. Juan C. Pascual MD, David Córcoles MD, Juan Castaño MD, et al. Hospitalization and Pharmacotherapy for Borderline Personality Disorder in a Psychiatric Emergency Service. *Psychiatr Serv* 2007;58(9):1199-204. doi: 10.1176/ps.2007.58.9.1199
11. Hong V. Borderline personality disorder in the emergency department: good psychiatric management. *Harv Rev Psychiatry* 2016;24(5):357-66. doi: 10.1097/HRP.0000000000000112
12. Gabbard GO, Wilkinson SM. Management of countertransference with borderline patients. Northvale, New Jersey: Jason Aronson Inc. 2000.
13. Nelson HD, Haney EM, Dana T, et al. Screening for Osteoporosis: An Update for the U.S. Preventive Services Task Force. *Ann Intern Med* 2010;153(2):99-111. doi: 10.7326/0003-4819-153-2-201007200-00262 %m 20621892
14. Powell C. The Delphi technique: myths and realities. *J Adv Nurs* 2003;41(4):376-82. doi: 10.1046/j.1365-2648.2003.02537.x
15. Jones J, Hunter D. Qualitative Research: Consensus methods for medical and health services research. *BMJ* 1995;311(7001):376-80. doi: 10.1136/bmj.311.7001.376
16. Gunderson J, Links P. Borderline Personality Disorder: A Clinical Guide. Washington, DC: American Psychiatric Association Publishing 2008.
17. Lau J. Models of interaction between clinical practice guidelines (CPG) groups and systematic review (SR) teams. IOM Committee on Standards for Developing Trustworthy Clinical Practice Guidelines meeting (January 12, 2010). Washington, DC, 2010.

For peer review only - http://bmjopen.bmj.com/site/about/guidelines.xhtml

18. Armstrong MJ, Rueda J-D, Gronseth GS, et al. Framework for enhancing clinical practice guidelines through continuous patient engagement. *Health Expect* 2017;20(1):3-10. doi: 10.1111/hex.12467
19. Excellence NIfHaC. The Guidelines Manual: National Institute for Health and Clinical Excellence (NICE), 2012.
20. Eden J, Levit L, Berg A, et al. Finding What Works in Health Care: Standards for Systematic Reviews. Washington, DC: Institute of Medicine (US) Committee on Standards for Systematic Reviews of Comparative Effectiveness Research, National Academies Press 2011.

For peer review only

Supplementary Materials

Protocol for a Delphi consensus study to develop guidelines for the management of borderline personality disorder in the emergency department

For peer review only

For peer review only - http://bmjopen.bmj.com/site/about/guidelines.xhtml

APPENDIX A

Protocol for systematic reviews

1. Review questions

We will conduct five systematic reviews to search the literature for articles on the following key questions (KQs) related to the key areas of care of people with borderline personality disorder (BPD) in the emergency department (ED):

1. What interventions in the ED are associated with benefit while minimising harm for people with BPD?
    a. What interventions in the ED are effective?
    b. What interventions in the ED are harmful?
2. What are the effective approaches to assess and manage the risk of suicide and self-harm of people with BPD?
3. What is the role of hospitalisation in managing people with BPD?
    a. What are the indications for hospitalisation?
    b. What is the effectiveness of hospitalisation?
    c. What are the harms of hospitalisation?
4. What aftercare services can help people with BPD who present to the ED but are not hospitalised?
5. What interventions in the ED for agitation and aggression are associated with benefit while minimising harm for people with BPD?
    a. What interventions for agitation and aggression are effective?
    b. What interventions for agitation and aggression are harmful?
6. What are effective approaches to involving family members and support persons in the care of people with BPD in the ED?
7. What are effective approaches to involving outpatient providers in the care of people with BPD in the ED?
8. What are effective approaches to providing optimal medical care for people with BPD presenting in a mental health crisis to the ED ?
9. What are the most effective environmental elements for people with BPD in the ED?

2. Searches

The following databases will be searched from inception: MEDLINE, MEDLINE In-Process, Embase, Global Health, and PsychInfo with no language restrictions. The results of the individual databases will be transferred into a reference manager program and duplicates will be eliminated. To search for additional reports, we will search the reference lists of the included studies. A PRISMA flow chart will be included in the final publication.

3. Search strategy

KQ1

1. ((borderline$ or cluster$) adj3 personalit$).mp

For peer review only - http://bmjopen.bmj.com/site/about/guidelines.xhtml

2. (borderline$ or BPD).mp
3. ((difficult$ or challeng$) adj3 patient$).mp
4. (recurrent$ or repeat$) adj3 (suicid$ or self-harm$ or self-injur$ or self-cut$ or mutilat$).mp
5. or/1-4
6. (emergency adj3 (department$ or room$ or treatment$ or medicine$ or health service$)).mp
7. 5 and 6

KQ2

1. ((borderline$ or cluster$) adj3 personalit$).mp
2. (borderline$ or BPD).mp
3. ((difficult$ or challeng$) adj3 patient$).mp
4. (recurrent$ or repeat$) adj3 (suicid$ or self-harm$ or self-injur$ or self-cut$ or mutilat$).mp
5. or/1-4
6. ((suicid$ or self harm$ or self injur$ or self cut$ or mutilat$ or NSSI or DSH) and (risk$ adj5 (assess$ or evaluat$))).mp
7. 5 and 6

KQ3

1. ((borderline$ or cluster$) adj3 personalit$).mp
2. (borderline$ or BPD).mp
3. ((difficult$ or challeng$) adj3 patient$).mp
4. (recurrent$ or repeat$) adj3 (suicid$ or self-harm$ or self-injur$ or self-cut$ or mutilat$).mp
5. or/1-4
6. (hospitalization$ or hospitalisation$ or admit$ or admission$).mp
7. (((single$ or double$ or trebl$ or tripl$) adj5 (blind$ or mask$)) or trial$ or (clinic$ adj2 trial$) or open-label$ or openlabel$ or (pre$ adj5 post$) or (pretest adj5 posttest) or cohort or (program$ adj5 evaluate$) or ((assurance$ or improv$ or indicator$ or measure$ or metric$) adj2 quality)).mp
8. 5 and 6 and 7

KQ4

1. ((borderline$ or cluster$) adj3 personalit$).mp
2. (borderline$ or BPD).mp
3. ((difficult$ or challeng$) adj3 patient$).mp
4. (recurrent$ or repeat$) adj3 (suicid$ or self-harm$ or self-injur$ or self-cut$ or mutilat$).mp
5. or/1-4
6. (emergency adj3 (department$ or room$ or treatment$ or medicine$ or health service$)).mp
7. (aftercare$ or (discharge adj5 (plan$ or service$ or manag$ or treatment$ or therap$))).mp
8. 5 and 6 and 7

For peer review only - http://bmjopen.bmj.com/site/about/guidelines.xhtml

KQ5

1. ((borderline$ or cluster$) adj3 personalit$).mp
2. (borderline$ or BPD).mp
3. ((difficult$ or challeng$) adj3 patient$).mp
4. (recurrent$ or repeat$) adj3 (suicid$ or self-harm$ or self-injur$ or self-cut$ or mutilat$).mp
5. or/1-4
6. (agitat$ or aggress$ or violen$ or anxiet$ or anxious$).mp
7. (((single$ or double$ or trebl$ or tripl$) adj5 (blind$ or mask$)) or trial$ or (clinic$ adj2 trial$) or open-label$ or openlabel$ or (pre$ adj5 post$) or (pretest adj5 posttest) or cohort or (program$ adj5 evaluate$) or ((assurance$ or improv$ or indicator$ or measure$ or metric$) adj2 quality)).mp
8. (emergency adj3 (department$ or room$ or treatment$ or medicine$ or health service$)).mp
9. 5 and 6 and 9 and 8

KQ6

1. ((borderline$ or cluster$) adj3 personalit$).mp
2. (borderline$ or BPD).mp
3. ((difficult$ or challeng$) adj3 patient$).mp
4. (recurrent$ or repeat$) adj3 (suicid$ or self-harm$ or self-injur$ or self-cut$ or mutilat$).mp
5. or/1-4
6. (emergency adj3 (department$ or room$ or treatment$ or medicine$ or health service$)).mp
7. (famil$ or partner$ or caregiver$ or parent$ or guardian$ or children$ or support$).mp
8. 5 and 6 and 7

KQ7

1. ((borderline$ or cluster$) adj3 personalit$).mp
2. (borderline$ or BPD).mp
3. ((difficult$ or challeng$) adj3 patient$).mp
4. (recurrent$ or repeat$) adj3 (suicid$ or self-harm$ or self-injur$ or self-cut$ or mutilat$).mp
5. or/1-4
6. (emergency adj3 (department$ or room$ or treatment$ or medicine$ or health service$)).mp
7. ((outpatient$ or ambulator$) adj5 (provider$ or team$ or caregiver$ or treater$ or physician$ or psychiatrist$ or psychologist$)).mp
8. 5 and 6 and 7

KQ8

1. ((borderline$ or cluster$) adj3 personalit$).mp
2. (borderline$ or BPD).mp
3. ((difficult$ or challeng$) adj3 patient$).mp

For peer review only - http://bmjopen.bmj.com/site/about/guidelines.xhtml

4. (recurrent$ or repeat$) adj3 (suicid$ or self-harm$ or self-injur$ or self-cut$ or mutilat$).mp
5. or/1-4
6. (emergency adj3 (department$ or room$ or treatment$ or medicine$ or health service$)).mp
7. ((medical$ or physical health$) adj3 (care$ or treatment$ or management$)).mp
8. 5 and 6 and 7

KQ9

1. ((borderline$ or cluster$) adj3 personalit$).mp
2. (borderline$ or BPD).mp
3. ((difficult$ or challeng$) adj3 patient$).mp
4. (recurrent$ or repeat$) adj3 (suicid$ or self-harm$ or self-injur$ or self-cut$ or mutilat$).mp
5. or/1-4
6. (emergency adj3 (department$ or room$ or treatment$ or medicine$ or health service$)).mp
7. (environment$ or architecture$ or design$ or layout$ or decor or ergonomic$).mp
8. 5 and 6 and 7

4. Condition or domain being studied

We will study the key areas of emergency care of people with BPD defined by our KQs.

5. Inclusion/exclusion criteria

Inclusion: the report is (1) a primary study (clinical trial, cohort study, cross-sectional study, pre/post evaluation, program evaluation, or quality assurance), guideline, standards, consensus statement, decision-making tool, or expert opinion, (2) includes a sample of at least >75% people with BPD in an emergency setting (if a primary study) or is about BPD in emergency settings (if a guideline, standard, consensus statement, decision-making tool, or expert opinion), and is about either (i) the overall care of people with BPD in the ED (KQ1), (ii) suicide/self-harm risk assessment and/or management of people with BPD (KQ2), (iii) hospitalisation of people with BPD (KQ3), (iv) aftercare services of people with BPD who present to the ED but are not hospitalised (KQ4), (v) the pharmacological and/or non-pharmacological management of agitation/aggression in people with BPD (KQ5), (vi) approaches to involving family members/support persons in the care of people with BPD in the ED (KQ6), (vii) approaches to involving outpatient providers in the care of people with BPD in the ED (KQ7), (viii) general medical care of people with BPD presenting to the ED in a mental health crisis (KQ8), and (ix) environmental elements (e.g., architecture, design, layout, décor, ergonomics, etc.) of the ED in relation to people with BPD presenting to the ED in a mental health crisis (KQ9).
Exclusion: none.

6. Main outcome(s)

For peer review only - http://bmjopen.bmj.com/site/about/guidelines.xhtml

The primary outcome for the primary studies is efficacy which will be categorized as follows. These outcomes will be summarized narratively along with an effect size if it can be calculated from the report. If an effect size cannot be calculated from the report, the outcome will be narratively summarized only.

1. *Suicidality outcomes*
   i. Self-harm
   ii. Suicidal ideation
   iii. Suicide attempt
   iv. Death by suicide
2. *BPD-related outcomes*
   i. Emotion dysregulation
   ii. Depression
   iii. Anxiety
   iv. Anger/irritability
   v. Aggression
   vi. Paranoia
   vii. Dissociation
   viii. Interpersonal dysfunction
   ix. Self dysfunction (e.g., identity disturbance)
3. *Global outcomes*
   i. Quality of life
   ii. Global psychosocial functioning
   iii. Global symptom severity
4. *Service utilization outcomes*
   i. Patient satisfaction
   ii. Hospitalisation
   iii. ED visits
   iv. Adherence with medications
   v. Adherence with appointments.
   vi. Time to all-cause treatment discontinuation

We will exclude measures when they cannot be categorized. Note, suicidal ideation, planning, and behaviour (including self-harm) will be always coded under behavioural dyscontrol, even if in the report it may have been coded as a tolerability outcome. The primary outcome for non-primary studies (i.e., guideline, standards, consensus statement, decision-making tool, or expert opinions) will be a narrative summary of the opinion on a given KQ.

Measures of effect: See Additional outcomes section below.

7. Additional outcome(s)

The secondary outcome for the primary studies is tolerability which will be categorized as follows. These outcomes will be summarized narratively along with an effect size if it can be calculated from the report. If an effect size cannot be calculated from the report, the outcome will be narratively summarized only. There will be no secondary outcomes for non-primary studies.

For peer review only - http://bmjopen.bmj.com/site/about/guidelines.xhtml

- Any adverse event
- Serious adverse event
- Death (non-suicide)
- Nausea
- Constipation
- Diarrhea
- Dry mouth
- Headache
- Dizziness
- Sedation
- Sleep disturbance
- Restlessness/agitation
- Menstrual changes
- Increased appetite
- Weight gain
- Decreased appetite
- Weight loss
- Weight loss
- Endpoint weight

- Extrapyramidal symptoms
- Akathisia
- Tardive dyskinesia
- Tremor (any type)
- Any other neurological dysfunction
- Cognitive dysfunction
- Sexual dysfunction
- Systolic blood pressure
- Diastolic blood pressure
- QTc interval
- Liver enzymes (AST, ALT, ALP, or GGT)
- Bilirubin (total, direct, or indirect)
- Albumin
- International normalized ratio (INR)
- Low-density lipoprotein (LDL)
- High-density lipoprotein (HDL)
- Triglycerides
- Total cholesterol
- Fasting blood glucose

- HbA1C
- Leukocyte count
- Hemoglobin
- Platelet count
- Creatinine
- Urea or blood urea nitrogen (BUN)
- Estimated glomerular filtration rate
- Sodium
- Potassium
- Calcium
- Phosphate
- Magnesium
- Creatine kinase
- Prolactin
- Thyroid stimulating hormone
- Thyroid hormone

For peer review only

Measures of effect:

For each primary study we will calculate the standardized mean difference (SMD, Hedges' *g*) for continuous measures (efficacy and tolerability data). If the study includes a comparator, we will use endpoint data between the active intervention vs. control intervention to calculate the SMD. If the study does not include a comparator, we will use pre vs. post data to calculate the SMD. If only change scores are reported, we used change scores to calculate the SMD between the active intervention vs. control intervention. If both are reported, endpoint scores will be used. Dichotomous outcomes measured throughout the study and reported at as totals at endpoint (e.g., total number of self-harm episodes during the trial) will be considered endpoint data. Intention-to-treat (ITT) data will be used whenever available. Negative SMDs will indicate the active intervention was more beneficial than control intervention. The relative risk (RR) will be

For peer review only - http://bmjopen.bmj.com/site/about/guidelines.xhtml

calculated for dichotomous tolerability data only. RR less than 1 will indicate the active intervention had less adverse effects than control intervention. Effect sizes of dichotomous efficacy data will be converted to the SMD using the procedure discussed shortly.

Combining multiple effect sizes within trials:

If a study reports multiple outcomes (efficacy or tolerability) per category, the effect sizes will be combined to produce a single composite effect size (SMD or RR) using the method in Borenstein et al (2009).[1] We will assume a within-cluster correlation between outcomes of $r = 0.45$. We will make this assumption because studies almost never report the inter-correlations between their outcome measures. $r = 0.45$ is the 95th percentile correlation across 708 meta-analytically-derived correlations in psychology between different constructs.[2] We used the 95th percentile since it gives a more conservative estimate of the correlation between similar outcomes within studies. This is because smaller correlations lower the variance of combined effect sizes.[1]

If a continuous outcome is reported alongside its dichotomous outcome (e.g., response rate based on ≥ 50% reduction in the scale), only the continuous outcome will be extracted. This is due to the redundancy and the lower statistical power of dichotomized continuous scales. In order to combine continuous (e.g., self-report scale of impulsivity) and dichotomous (e.g., number of aggressive episodes) efficacy data within a symptom category (e.g., behavioural dyscontrol) we will perform the following procedure: an odds ratio (OR) will be calculated for the dichotomous outcome and converted to Cohen's *d* using established equations.[1] We will apply the *J* correction factor to convert the Cohen's *d* to Hedges' *g* (the SMD).[2] This will be combined with the SMD of the continuous outcome(s) within the symptom category. Note, the RR is preferred over the OR for RCTs and cohort studies. The OR approximates the RR when the incidence of the outcome is low (< 10%). As the incidence increases, the OR overestimates the RR when it is greater than 1 and underestimates the RR when it's less than 1.[3] To the best of our knowledge there are no established procedures to convert RRs directly to SMDs. Therefore, while imperfect, we will use this procedure with dichotomous outcomes given the advantages of combing multiple outcomes into a single effect size per symptom category.

8. Data extraction (selection and coding)

1. **Selection of studies:** two reviewers will independently screen the titles and abstracts of the search results to determine potential eligibility. Full-text reports of the potentially eligible reports will then be retrieved and assessed by two reviewers using our inclusion/exclusion criteria to determine eligibility. Disagreements at either the screening or full-text review stages of the selection phase will be resolved through consensus between the two reviewers and, if necessary, a third reviewer. Decisions regarding eligibility will be documented.
2. **Data extraction:** Two reviewers will independently extract the main outcomes and additional outcomes from the reports into standardized electronic databases. Discrepancies in data extraction will be resolved through consensus between the two authors and, if necessary, a third author.

9. Risk of bias assessment

For peer review only - http://bmjopen.bmj.com/site/about/guidelines.xhtml

Risk of bias in the randomized controlled trials (RCTs) will be assessed using the Cochrane Risk of Bias 2 (ROB 2). Risk of bias in the non-randomized primary studies will be assessed using the Cochrane Risk of Bias in Non-randomized Studies of Interventions (ROBINS-I). Risk of bias will be assessed by two independent reviewers. All disagreements will be resolved through consensus between the two reviewers and a third reviewers if necessary. ROBINS-I requires listing in the protocol the confounding domains and co-interventions. Confounding domains and co-interventions are those for which adjustment is expected to lead to a clinically important change in the estimated effect of the intervention. We defined the following:

1. **Confound domains:** age, sex, race/ethnicity, comorbidities, socioeconomic status.
2. **Co-interventions:** concurrent psychotherapy or pharmacotherapy.

10. Strategy for data synthesis

The characteristics of the included primary studies will be summarized in a tabular form using the PICO format along with a narrative summary of the results with the effect sizes of the efficacy/tolerability data when available. When appropriate, quality of evidence will be assessed using the GRADE approach.[4] The narrative summaries of the opinions from the non-primary studies will be summarized in tabular form.

For peer review only

APPENDIX B

The following pivotal guidelines, psychotherapy manuals, book chapters, and articles will be reviewed as part of the qualitative assessment in order to extract opinions relevant to our key questions.

Guidelines

1. National Institute for Health and Clinical Excellence (NICE) guideline on treatment and management: Borderline personality disorder[5]
2. American Psychiatric Association (APA) practice guideline for the treatment of patients with borderline personality disorder[6]
3. National Health and Medical Research Council (NHMRC) clinical practice guideline for the management of borderline personality disorder[7]
4. World Federation of Societies of Biological Psychiatry (WFSBP) Guidelines for biological treatment of personality disorders[8]
5. The project BETA guidelines[9]
6. Garriga et al (2016) Assessment and management of agitation in psychiatry: Expert consensus. *The World Journal of Biological Psychiatry*.[10]

Psychotherapy manuals

1. Linehan (1993) Cognitive-behavioral treatment of borderline personality disorder.[11]
2. Bateman & Fonagy (2016) Mentalization-based treatment for personality disorders: A practical guide.[12]
3. Yeomans et al (2015) Transference focused psychotherapy for borderline personality disorder: A Clinical Guide.[13]
4. Kernberg (1984) Severe personality disorders: Psychotherapeutic Strategies.[14]
5. Gunderson & Links (2008) Borderline personality disorder: A clinical guide.[15]
6. Gunderson & Links (2014) Handbook of good psychiatric management for borderline personality disorder.[16]
7. Arntz & van Genderen (2021) Schema therapy for borderline personality disorder, 2nd Ed.[17]
8. Davidson (2008) Cognitive therapy for personality disorders: A guide for clinicians, 2nd Ed.[18]
9. Black & Blum (2016) Systems training for emotional predictability and problem solving for borderline personality disorder: Implementing STEPPS around the globe.[19]
10. Dawson & MacMillan (1993) Relationship management of the borderline patient: From understanding to treatment.[20]
11. Livesley (2003) Practical management of personality disorder.[21]
12. Livesley (2017) Integrated modular treatment for borderline personality disorder: A practical guide to combining effective treatment methods.[22]

Other books

1. Gabbard (2014) Psychodynamic psychiatry in clinical practice, 5th Ed.[23]
2. Gabbard & Wilkinson (2000) Management of countertransference with borderline patients.[24]

For peer review only - http://bmjopen.bmj.com/site/about/guidelines.xhtml

3. Choi-Kain & Gunderson (2019) Applications of good psychiatric management for borderline personality disorder.[25]
4. Sonley & Choi-Kain (2020) Good psychiatric management and dialectical behavior therapy: A clinician's guide to integration and stepped care.[26]

Book chapters

1. Hong & Casher (2020) The inpatient with borderline personality disorder. In: *Manual of Inpatient Psychiatry, 2nd Ed*.[27]
2. Hong et al (2020) Personality disorders. In: *Emergency Psychiatry: Principles and Practice, 2nd Ed*.[28]
3. Hong et al (2019) Traitements dans les services d'urgence. In: *Trouble de la Personnalite Borderline: Pratiques Therapeutiques*.[29]
4. Curry & Riddle (2021) Personality disorders in the emergency department. In: *Behavioral Emergencies for Healthcare Providers*.[30]

Published papers

1. Hong (2016) Borderline personality disorder in the emergency department: good psychiatric management. *Harvard Review of Psychiatry*.[31]
2. Hong et al (2019) Antisocial and borderline personality disorders in the emergency department: conceptualizing and managing "Malingered" or "Exaggerated" symptoms. *Current Behavioral Neuroscience Reports*.[32]
3. Shaikh et al (2017). Patients with borderline personality disorder in emergency departments. *Frontiers in Psychiatry*.[33]
4. Broadbear et al (2022). Emergency department utilisation by patients with a diagnosis of borderline personality disorder: An acute response to a chronic disorder. *Emergency Medicine Australasia*.[34]
5. Cases et al (2020). Evidence of practice gaps in emergency psychiatric care for borderline personality disorder: how can this be explained? *BMC Psychiatry*.[35]
6. Acres et al (2021). From the community to the emergency department: A study of hospital emergency department nursing practices from the perspective of carers of a loved one with Borderline Personality Disorder. *Health & social care in the community*.[36]
7. Paris (2002) Chronic suicidality among patients with borderline personality disorder. *Psychiatric Services*.[37]
8. Paris (2004) Understanding self-mutilation in borderline personality disorder. *Harvard Review of Psychiatry*.[38]
9. Paris (2004) Half in love with easeful death: The meaning of chronic suicidality in borderline personality disorder. *Harvard Review of Psychiatry*.[39]
10. Paris (2004) Is hospitalization useful for suicidal patients with borderline personality disorder? *Journal of Personality Disorders*.[40]
11. Paris (2006) Predicting and preventing suicide: Do we know enough to do either? . *Harvard Review of Psychiatry*.[41]
12. Paris (2021) Can we predict or prevent suicide?: An update. *Preventative Medicine*.[42]
13. Goodman et al (2012) Suicidal risk and management in borderline personality disorder. *Current Psychiatry Reports*.[43]

For peer review only - http://bmjopen.bmj.com/site/about/guidelines.xhtml

References

1. Borenstein M, Hedges LV, Higgins JP, et al. Introduction to Meta-analysis. Chichester, UK: Wiley 2009.
2. Gignac GE, Szodorai ET. Effect size guidelines for individual differences researchers. *Personality and individual differences* 2016;102:74-78.
3. Zhang J, Kai FY. What's the relative risk?: A method of correcting the odds ratio in cohort studies of common outcomes. *JAMA* 1998;280(19):1690-91.
4. Guyatt GH, Oxman AD, Vist GE, et al. GRADE: an emerging consensus on rating quality of evidence and strength of recommendations. *BMJ* 2008;336(7650):924. doi: 10.1136/bmj.39489.470347.AD
5. Health NCCfM. Borderline Personality Disorder: The NICE Guideline on Treatment and Management. National Clinical Practice Guideline No. 78: The British Psychological Society and The Royal College of Psychiatrists, 2009.
6. Association AP. Practice guideline for the treatment of patients with borderline personality disorder: American Psychiatric Association Practice Guidelines 2001.
7. Smith M, Chanen A, Ford F, et al. Clinical practice guideline for the management of borderline personality disorder. Melbourne: National Health and Medical Research Council (NHMRC), 2012.
8. Herpertz SC, Zanarini M, Schulz CS, et al. World Federation of Societies of Biological Psychiatry (WFSBP) guidelines for biological treatment of personality disorders. *The World Journal of Biological Psychiatry* 2007;8(4):212-44.
9. Holloman Jr GH, Zeller SL. Overview of Project BETA: best practices in evaluation and treatment of agitation. *Western Journal of Emergency Medicine* 2012;13(1):1.
10. Garriga M, Pacchiarotti I, Kasper S, et al. Assessment and management of agitation in psychiatry: Expert consensus. *The World Journal of Biological Psychiatry* 2016;17(2):86-128. doi: 10.3109/15622975.2015.1132007
11. Linehan M. Cognitive-behavioral Treatment of Borderline Personality Disorder. New York City, NY: Guilford Press 1993.
12. Bateman A, Fonagy P. Mentalization based treatment for personality disorders: A practical guide. Oxford, UK: Oxford University Press 2016.
13. Yeomans FE, Clarkin JF, Kernberg OF. Transference-Focused Psychotherapy for Borderline Personality Disorder: A Clinical Guide. Arlington, VA: American Psychiatric Association Publishing 2015.
14. Kernberg OF. Severe Personality Disorders: Psychotherapeutic Strategies. New Haven, CT: Yale University Press 1984.
15. Gunderson J, Links P. Borderline Personality Disorder: A Clinical Guide. Washington, DC: American Psychiatric Association Publishing 2008.
16. Gunderson J, Links P. Handbook of Good Psychiatric Management for Borderline Personality Disorder. Washington, DC: American Psychiatric Association Publishing 2014.
17. Arntz A, Van Genderen H. Schema therapy for borderline personality disorder. West Sussex, UK: John Wiley & Sons, Ltd 2021.
18. Davidson K. Cognitive therapy for personality disorders: A guide for clinicians. 2nd ed. East Sussex, UK: Routledge 2008.

For peer review only - http://bmjopen.bmj.com/site/about/guidelines.xhtml

19. Black DW, Blum N. Systems training for emotional predictability and problem solving for borderline personality disorder: Implementing STEPPS around the globe. New York City, NY: Oxford University Press 2017.
20. Dawson DL, MacMillan HL. Relationship management of the borderline patient: from understanding to treatment. New York City, NY: Routledge 1993.
21. Livesley J. Practical Management of Personality Disorder. New York City, NY: Guilford Press 2003.
22. Livesley J. Integrated Treatment for Borderline Personality Disorder: A Practical Guide to Combining Effective Treatment Methods. Cambridge, UK: Cambridge University Press 2017.
23. Gabbard GO. Psychodynamic psychiatry in clinical practice. 5th ed. Arlington, VA: American Psychiatric Association Publishing 2014.
24. Gabbard GO, Wilkinson SM. Management of countertransference with borderline patients. Northvale, New Jersey: Jason Aronson Inc. 2000.
25. Choi-Kain LW, Gunderson JG. Applications of good psychiatric management for borderline personality disorder: a practical guide. Washington, DC: American Psychiatric Association Publishing 2019.
26. Sonley AK, Choi-Kain LW. Good psychiatric management and dialectical behavior therapy: A clinician's guide to integration and stepped care. Washington, DC: American Psychiatric Association Publishing 2021.
27. Hong V, Casher M. The inpatient with borderline personality disorder. In: Casher M, Bess J, eds. Manual of Inpatient Psychiatry. 2nd ed. Cambridge, UK: Cambridge University Press 2020.
28. Hong V, Reddy P, Entenman S. Personality Disorders. In: Glick R, Zeller S, Berlin J, eds. Emergency Psychiatry: Principles and Practice. 2nd ed. Philadelphia, PA: Wolters Kluwer 2020.
29. Hong V, Kozada K, Shobassy A. Traitements dans les services d'urgence. In: Kolly S, Charbon P, Kramer U, eds. Trouble de la Personnalite Borderline: Pratiques Therapeutiques France: Elsevier Masson SAS 2019.
30. Curry A, Riddle M. Personality Disorders in the Emergency Department. In: Zun L, Nordstrom K, Wilson M, eds. Behavioral Emergencies for Healthcare Providers. 2nd ed. Switzerland: Springer Nature 2021.
31. Hong V. Borderline personality disorder in the emergency department: good psychiatric management. *Harv Rev Psychiatry* 2016;24(5):357-66. doi: 10.1097/HRP.0000000000000112
32. Hong V, Pirnie L, Shobassy A. Antisocial and borderline personality disorders in the emergency department: conceptualizing and managing "Malingered" or "Exaggerated" symptoms. *Current Behavioral Neuroscience Reports* 2019;6(4):127-32.
33. Shaikh U, Qamar I, Jafry F, et al. Patients with borderline personality disorder in emergency departments. *Frontiers in psychiatry* 2017;8:136.
34. Broadbear JH, Rotella J-A, Lorenze D, et al. Emergency department utilisation by patients with a diagnosis of borderline personality disorder: An acute response to a chronic disorder. *Emerg Med Australas* 2022;34(5):731-37.
35. Cases C, Lafont Rapnouil S, Gallini A, et al. Evidence of practice gaps in emergency psychiatric care for borderline personality disorder: how can this be explained? *BMC Psychiatry* 2020;20(1):476. doi: 10.1186/s12888-020-02892-7

For peer review only - http://bmjopen.bmj.com/site/about/guidelines.xhtml

36. Acres K, Loughhead M, Procter N. From the community to the emergency department: A study of hospital emergency department nursing practices from the perspective of carers of a loved one with Borderline Personality Disorder. *Health Soc Care Community* 2022;30(5):1789-97.
37. Joel Paris, M.D. Chronic Suicidality Among Patients With Borderline Personality Disorder. *Psychiatr Serv* 2002;53(6):738-42. doi: 10.1176/appi.ps.53.6.738
38. Paris J. Understanding Self-mutilation in Borderline Personality Disorder. *Harv Rev Psychiatry* 2005;13(3):179-85. doi: 10.1080/10673220591003614
39. Paris J. Half in Love with Easeful Death: The Meaning of Chronic Suicidality in Borderline Personality Disorder. *Harv Rev Psychiatry* 2004;12(1):42-48. doi: 10.1080/10673220490279161
40. Joel Paris. Is Hospitalization Useful for Suicidal Patients with Borderline Personality Disorder? *J Personal Disord* 2004;18(3):240-47. doi: 10.1521/pedi.18.3.240.35443
41. Paris J. Predicting and Preventing Suicide: Do We Know Enough to Do Either? *Harv Rev Psychiatry* 2006;14(5):233-40. doi: 10.1080/10673220600968662
42. Paris J. Can we predict or prevent suicide?: An update. *Prev Med* 2021;152(Pt 1):106353. doi: 10.1016/j.ypmed.2020.106353 [published Online First: 2021/09/21]
43. Goodman M, Roiff T, Oakes AH, et al. Suicidal Risk and Management in Borderline Personality Disorder. *Curr Psychiatry Rep* 2012;14(1):79-85. doi: 10.1007/s11920-011-0249-4

For peer review only

For peer review only - http://bmjopen.bmj.com/site/about/guidelines.xhtml

BMJ Open

Protocol for a Delphi consensus study to develop guidelines for the management of adults with borderline personality disorder in the emergency department

Journal:	*BMJ Open*
Manuscript ID	bmjopen-2023-075119.R1
Article Type:	Protocol
Date Submitted by the Author:	28-Sep-2023
Complete List of Authors:	Prosser, Aaron; McMaster University, Psychiatry<br>Hong, Victor; University of Michigan, Department of Psychiatry<br>Helfer, Bartosz; University of Wroclaw, Institute of Psychology; University of Wroclaw, Meta-Research Centre<br>Fudge, David; Hamilton, Department of Psychiatry and Behavioural Neurosciences<br>Patterson, Janet; Hamilton, Department of Psychiatry and Behavioural Neurosciences<br>Rosebush, Patricia; Hamilton, Department of Psychiatry and Behavioural Neurosciences<br>Frey, Benicio; McMaster University, McMaster Integrative Neuroscience Discovery and Study (MiNDS); St. Joseph's Healthcare Hamilton, Mood Disorders Program<br>Links, Paul; Hamilton, Department of Psychiatry and Behavioural Neurosciences
**Primary Subject Heading**:	Mental health
Secondary Subject Heading:	Emergency medicine
Keywords:	PSYCHIATRY, ACCIDENT & EMERGENCY MEDICINE, Personality disorders < PSYCHIATRY, Suicide & self-harm < PSYCHIATRY

SCHOLARONE™
Manuscripts

Protocol for a Delphi consensus study to develop guidelines for the management of adults with borderline personality disorder in the emergency department

Aaron Prosser MD MSc[1]
Victor Hong MD[2]
Bartosz Helfer PhD MSc[3,4]
David Fudge MD[1]
Janet Patterson MD[1]
Patricia Rosebush MD[1]
Benicio N. Frey MD PhD[1]
Paul Links MD MSc[1]

[1]Department of Psychiatry and Behavioural Neurosciences, McMaster University, Hamilton, Ontario, Canada
[2]Department of Psychiatry, University of Michigan, Ann Arbor, MI, United States
[3]Institute of Psychology, University of Wroclaw, Wroclaw, Poland
[4]Meta-Research Centre, University of Wroclaw, Wroclaw, Poland

Corresponding author: Aaron Prosser (aaron.prosser@medportal.ca)

Word count: 4,560

For peer review only

ABSTRACT

Introduction: Clinicians caring for adults with borderline personality disorder (BPD) in acute settings such as the emergency department (ED) have little evidence/guidance to base decisions on. Specific/detailed guidance for managing BPD in the ED is needed given the morbidity and mortality risks, high service utilization, unique challenges, and risk of iatrogenic interventions. The primary objective of this study is to use a consensus method to develop a guideline for managing adults with BPD in the ED. This protocol and the key questions for the guideline were developed with the advice of people with BPD and their family members/support persons.

Methods and analysis: We will perform a 4-phase Delphi study of an expert panel of clinicians, researchers, adults with BPD, and their family members/support persons. Various disciplines (psychiatry, psychology, emergency medicine, nursing, social work) and treatment approaches will be included in the expert panel. An online questionnaire will be developed from systematic reviews, qualitative assessments of pivotal literature, and opinions suggested by the panel (Phase 1). The panel will rate their agreement on opinions for each key question covering areas of emergency care of adults with BPD using two rounds of this questionnaire (Phases 2 and 3). Opinions meeting pre-defined thresholds for consensus will be brought to consensus meetings moderated by an independent chair (Phase 4). The purpose of these meetings is to finalise the set and phrasing of the opinions for each area of emergency care. These final opinions will be the recommendations in the guideline. If there are significant differences of opinion, the guideline will present both recommendations alongside one another.

Ethics and dissemination: This study has received ethics approval by the Hamilton Integrated Research Ethics Board in Hamilton, Ontario, Canada. The results of this study will be disseminated through peer-reviewed publications, conferences, and national professional and patient/family/support associations.

For peer review only

For peer review only - http://bmjopen.bmj.com/site/about/guidelines.xhtml

STRENGTHS AND LIMITATIONS OF THIS STUDY

- The primary objective of this study is to develop a guideline for managing adults with BPD in the ED, incorporating the advice and feedback of people with BPD and their family members/support persons.
- The Delphi method will be used due to the limited research in this area to inform a guideline.
- Our methods use a questionnaire to minimise the disadvantages of group discussion (e.g., domination by individuals or pressure to conform) while the consensus meeting capitalises on its advantages (e.g., clarifying ambiguities or editing the phrasing of recommendations).
- The expert panel will include participants from different disciplines, backgrounds, and treatment approaches including adults with BPD, their family members and support persons, clinicians, and researchers.
- The expert panel might not be fully representative since recruitment is not based on random sampling and the recommendations will be based on consensus, which is based on shared knowledge and practical experience rather than scientific proof.

For peer review only

For peer review only - http://bmjopen.bmj.com/site/about/guidelines.xhtml

INTRODUCTION

The need for guidelines

Borderline personality disorder (BPD) is a highly heritable (~55%) condition whose core pathology is a disturbance in identity, attachment, and emotion regulation [1, 2]. BPD is often viewed as a trauma disorder since for many it emerges from psychosocial adversity interacting with a genetic diathesis [1, 2]. The symptomatic expression of BPD includes dysphoria, dysregulation, recurrent self-harm/suicide attempts, and conflicted/unstable relationships [3]. These symptoms are often severe and disabling, causing frequent crises and presentations to the emergency department (ED), with frequent suicidal thoughts and self-harm behaviors being behavioral trademarks of the disorder. Specialized outpatient treatments for BPD have been developed and shown to be effective in randomised controlled trials (RCTs) [4]. There is also limited RCT evidence for some medications targeting specific clusters of symptoms (e.g., emotion dysregulation, impulsivity) [5, 6]. These developments are a major accomplishment and have informed guidelines on BPD, notably the NICE [7], APA [8], and NHMRC [9] guidelines.

While crises and acute treatment are addressed in these guidelines, the sections are brief since most of the evidence pertains to outpatient settings. Therefore, we formed a Guideline Working Group (GWG) to oversee the development of a guideline on managing BPD in the ED. Our focus is on adults with BPD, although certainly many of the principles may apply to adolescents. Our primary aim will be to identify a set of recommendations for each key question (KQ) using Delphi consensus methods. Each KQ defines a key area of the emergency care of adults with BPD. Specific and detailed guidance for managing adults BPD in the ED is needed given the common pitfalls related to high service utilization, unique challenges in the emergency setting, risk of iatrogenic interventions, and recurrent suicide attempts and self-harm.

High service utilization and unique challenges

Adults with BPD are frequent visitors to the ED, representing upwards of 9% of all visits in some jurisdictions, with recurrent visits being common [10]. Reasons for presenting to the ED are numerous, including self-harm, suicide attempts, situational crises, adjustment reactions, depression, anxiety, emotion dysregulation, agitation, intoxication, withdrawal, substance induced psychosis, and housing/financial problems. There are patient, staff, and systems factors which create unique challenges to managing BPD in ED settings [11]. Some are listed in Table 1.

Table 1. Challenges to managing BPD in ED settings

Patient	Staff	System
• Recurrent ED visits and high service utilization.<br>• Disruptive behaviours.<br>• Chronic high risk for suicide and self-harm.<br>• Transference reactions.	• Problematic staff attitudes.<br>• Varying levels of training.<br>• Ill-defined/time-limited responsibility for patients.<br>• Countertransference reactions.	• Transient/changing ED staff.<br>• Changing ED workloads.<br>• Disposition problems.<br>• Availability of inpatient beds, aftercare services, and rapid outpatient follow-up.

High risk of iatrogenic interventions in the emergency department

For peer review only - http://bmjopen.bmj.com/site/about/guidelines.xhtml

Adults with BPD appear to be at higher risk of iatrogenic interventions in all clinical settings, particularly acute settings like the ED [11]. These include inadequate risk assessment, unnecessary inpatient hospitalisations, excessive use of medications, or unnecessary use of seclusion or physical/chemical restraints. Staff attitudes and countertransference reactions can also drive iatrogenesis. Staff who are otherwise empathic, validating, supportive, and effective clinicians may have negative or positive reactions which – if not examined, accepted, and contained – can be the driver of harmful interventions [12]. Negative reactions include withdrawal, rejection, hostility, helplessness, hopelessness, anxiety, or feeling overwhelmed. Positive yet often unhelpful reactions include rescue fantasies or feeling excessively protective of or responsible for a patient. Lack of awareness of the separation of acute and chronic risk factors may play a role in these reactions. Given the prevalence of comorbidities in individuals with BPD, this adds further complexity to the clinical decision making [13]. Iatrogenesis is likely caused by an interplay between these patient, staff, and systems factors, often amplified in the ED.

Risk of recurrent suicide attempts and self-harm

Recurrent suicidal acts, threats, urges and self-harm behaviours are defining symptoms of BPD.[3] The risk of suicide is 3%-10% in people with BPD and the average number of suicide attempts per individual is 3, with completed suicide occurring about once per 23 attempts [1]. About 75% of people with BPD self-harm and among these 90% repeatedly self-harm [1]. Taking on this risk is inherent to the work of caring for people with BPD. These risks are greatest for clinicians working in the ED because people with BPD presenting to the ED often do so because they are acutely dysphoric, actively suicidal, engaged in severe self-harm, and/or attempted to end their life. However, discerning the difference between acute vs. chronic suicide/self-harm risk is difficult when a patient is chronically at high risk. This is especially the case when the risk assessment/management must be done by a clinician who doesn't have a longitudinal relationship with the patient, which is the reality for most clinicians working in the ED. For these reasons, many clinicians experience fear not only for the safety of the people with BPD they care for, but also their own medico-legal liability. This is yet another reason why specific and detailed guidance for managing BPD in the ED is needed, especially regarding suicide/self-harm risk assessment/management.

METHODS AND ANALYSIS

Rationale for the Delphi method

Our project started by first determining the most appropriate method to develop the guideline. Guidelines are usually constructed from systematic reviews and meta-analyses of primary studies. However, our group was already aware that there is scant research in this area. This was confirmed by pilot systematic searches. Therefore, we decided to conduct a Delphi consensus study to come up with recommendations from clinicians, researchers, patients, and family members/support persons.

Development of key questions

For peer review only - http://bmjopen.bmj.com/site/about/guidelines.xhtml

Our KQs were built from the two main tasks which need to be addressed for all people with BPD who come to the ED. We used the analytic-graphical model as developed by Nelson et al. (2010) [14] to visualize the analytic framework and our KQs (Figure 1). A primary consideration in the ED is the risk assessment (KQ2) and, if a diagnosis isn't established yet, screening for BPD (KQ3). This usually starts with the emergency medicine team who may or may not ask the psychiatrist and/or mental health clinicians (e.g., psychiatric nurses, social workers) for further assistance in the assessment and disposition planning. Another consideration is to decide if to admit the patient to hospital (KQ4) or discharge the person to aftercare psychosocial services (KQ5). Alongside this process is the overall care of the person with BPD while they are in the ED (KQ1). Here we mean the *general interventions* to use with all patients throughout their stay in the ED (e.g., empathy, validation, management of countertransference and bias) and *specific interventions* (e.g., psychoeducation, medications) to address the specific needs of a patient. A related key area are interventions for the management of agitation and aggression in people with BPD in the ED (KQ6). Another key area is the involvement and role of family members and support persons (KQ7) and outpatient providers (KQ8) while people with BPD are in the ED. The last key areas are approaches to providing optimal medical care (KQ9) and effective environmental elements (e.g., physical layout, interior design) for people with BPD presenting to the ED in a mental health crisis (KQ10).

Based on this analytic framework, we constructed ten KQs:

1. What psychosocial and pharmacological interventions in the ED are associated with benefit while minimising harm for adults with BPD, with considerations regarding potential comorbidities?
    a. What interventions in the ED are effective?
    b. What interventions in the ED are harmful?
2. What are the effective approaches to assess and manage the risk of suicide and self-harm of adults with BPD, with considerations regarding potential comorbidities?
3. What should be included as part of the emergency evaluation of adults to screen for previously undiagnosed BPD?
4. What is the role of hospitalisation in managing adults with BPD?
    a. What are the indications for hospitalisation?
    b. What is the effectiveness of hospitalisation?
    c. What are the harms of hospitalisation?
5. What psychosocial aftercare services (including partial hospitalisation) can help adults with BPD who present to the ED but are not hospitalised?
6. What interventions in the ED for agitation and aggression are associated with benefit while minimising harm for adults with BPD?
    a. What interventions for agitation and aggression are effective?
    b. What interventions for agitation and aggression are harmful?
7. What are effective approaches to involving family members and support persons in the care of adults with BPD in the ED?
8. What are effective approaches to involving outpatient providers in the care of adults with BPD in the ED?
9. What are effective approaches to providing optimal medical care for adults with BPD presenting in a mental health crisis to the ED ?

For peer review only - http://bmjopen.bmj.com/site/about/guidelines.xhtml

10. What are the most effective environmental elements (physical layout and interior design) for adults with BPD in the ED?

We will systematically review the literature on each KQ, the results of which will be integrated into the Delphi process (see methods section for details). The Delphi process will be used to provide guidance on each of these KQs. It will draw on the expertise of people across disciplines (psychiatry, psychology, emergency medicine, nursing, social work), backgrounds (clinicians, researchers, patients, family, support persons), and treatment approaches. The latter will hopefully include Dialectical Behavior Therapy (DBT), Transference-Focused Psychotherapy (TFP), Mentalization-Based Treatment (MBT), Good Psychiatric Management (GPM), Schema Focused Therapy (SFT), Cognitive Behavior Therapy (CBT), Systems Training for Emotional Predictability and Problem Solving (STEPPS), Integrated Modular Treatment (IMT), and psychopharmacology. Other content experts who may not align with a specific treatment approach will be contacted for participation. While developing this protocol and the KQs included here, we sought advice from people with BPD and their family members/support persons on what are the most important questions to include from their perspective as well as feedback on an earlier draft of the protocol (additional study development and design details are in Appendix A).

Study design

This study will use the Delphi method in order to achieve consensus across the group of participants. This method is appropriate for our purposes because it maximises the benefits of group discussion while minimising its disadvantages [15]. The main disadvantages are domination by powerful individuals, coalitions, vested interests, the biasing effects of personality and seniority, that only one person can speak at a time, and group pressures for conformity. The Delphi method provides a structured, democratic, transparent, and iterative procedure with controlled feedback to participants to determine the extent of agreement amongst a group about a given issue [15, 16]. The procedure will consist of questionnaire development (Phase 1), rating evaluation (Phase 2), re-rating (Phase 3), and consensus meeting (Phase 4). Phase 1 will include systematic reviews of our KQs to incorporate any recommendations which may come from the existing literature.

Study governance

The GWG is comprised of six psychiatrists (VH, DF, JP, PR, BF, PL), a psychiatry resident (AP), and a meta-researcher (BH) with expertise in guideline development. All clinicians in the GWG currently treat people with BPD in outpatient, inpatient, and emergency settings. One of the members (PL) was a co-developer of GPM [1, 17] and another (VH) published an expert opinion on the management of BPD in the ED based on GPM principles [11]. As a result, PL and VH will also be participants given their recognized expertise. However, this also is a potential source of bias. We detail how we will mitigate this (Appendix A). Study methods for the protocol were established in virtual meetings and email correspondence. The GWG will meet at minimum once quarterly and at higher frequency as needed until projected completion. Meetings will be held virtually and we will have regular email correspondence. To further address any outstanding issues with study governance, we will follow the complete

For peer review only - http://bmjopen.bmj.com/site/about/guidelines.xhtml

interaction approach with full transparency between the systematic review team and eventual guideline developers [18].

Involvement of persons with BPD and their families/support persons

Adults with BPD, their family members and support persons will have a voice in these guidelines since the recommendations will directly impact them. We recognize that there may be differences of opinion between patients/family/support persons and clinicians/researchers on some questions during the Delphi process. If there are significant differences in opinion, we will be transparent about these differences in the guideline. This ensures that patient/family/support persons voices are not silenced by majority opinion since we anticipate there will be more clinician/researcher participants. Patients or patients' representatives will be continuously involved throughout the guideline development process following a modified model by Armstrong et al. (2010) [19]. Following NICE recommendations, at least at least two patient/caregiver/advocate members will be involved in the Delphi process and consensus meeting as panelists [20].

Participant selection/recruitment

Participants will be included if they are clinicians and/or researchers who have significant experience in the management of BPD in the ED. We will also recruit patients and family members/support persons. The clinician/researcher sample will be recruited using a number of strategies. First, we will contact national professional organizations for emergency medicine, psychiatry, nursing, and social work in English-speaking countries, specifically the United States, Canada, United Kingdom, Ireland, South Africa, Australia, and New Zealand. We will ask them to advertise our study through their email mailing lists. Second, the GWG is already aware of a number of experts who we will invite to participate. These individuals were either a developer or published expert in a treatment approach for BPD, a clinician/group member on the NICE, APA, and NHMRC guidelines, or is a published expert in the management of BPD in the ED. Third, the publication of this protocol will also help advertise this study. Patients and family members/support persons will be recruited by contacting national patient advocacy and support organizations. We will ask these organizations to advertise our study through their email mailing lists and social media to help with recruitment. We will ask all participants to provide suggestions too. Readers of this protocol may also give suggestions. Suggestions can be made to the GWG by contacting the corresponding author of this protocol (aaron.prosser@medportal.ca). The deadline for suggesting a participant is 3 months after the publication date of this protocol.

Representation

We believe that representation is important to the guideline development process. Thus we will attempt to build a balanced panel of experts based on the following demographic data: discipline (psychiatry, psychology, nursing, emergency medicine, social work), group (clinician, researcher, adult with BPD, family member/support person), treatment modality allegiance, ethnicity, gender, and sexual orientation. In order to do this, at the recruitment stage, the study coordinator will email a form to potential participants who have expressed interest to participate. In this form, participants will be invited to provide basic demographic data to allow us to build a

For peer review only - http://bmjopen.bmj.com/site/about/guidelines.xhtml

balanced panel. There will be an option to select "Prefer not to answer", "Do not know", and "Other" for all questions. If a demographic factor has reached saturation, the person may be ineligible to participate. This demographic data will be collected before consent is obtained to participate in this study. Therefore, if a person is ineligible to participate or doesn't consent to participate in the study after reviewing the consent form, the demographic form will be promptly deleted and will not be kept part of the study records. If the person is eligible to participate and consents, the form will be retained as part of the study records, de-identified, and statistically summarized so that we can show the representativeness of the expert panel in the publication of the guideline. Saturation will be determined on a case-by-case basis depending on our pool of potential participants and the representativeness of those who have already consented to participate.

Delphi procedure

The Delphi process is an iterative procedure which typically proceeds in three phases [16]. We will add a fourth phase in order to have a consensus meeting moderated by an independent chair, thus combining the Delphi method with the consensus conference method. This is because we believe that an open-ended group discussion has advantages over questionnaires. In particular, group discussion can be helpful for clarifying ambiguities and differences and editing phrasing for the final recommendations of the guideline. Furthermore, a meeting provides an opportunity to reach consensus on issues where consensus has not yet been achieved. Questionnaires will be delivered and collected from participants using REDCap. Data will be de-identified by giving participants an arbitrary study ID linked to their questionnaire responses, however no identifying data will be collected (e.g., names, age, date of birth) with the questionnaire responses.

Phase 1 – Questionnaire development

Phase 1 has three goals: (1) create the expert panel, (2) create a pool of opinions on each KQ from three sources of information, and (3) create an online questionnaire using this pool of opinions for rating evaluation by the expert panel. The expert panel will be created using the selection and recruitment strategies described above.

The first source of information we will use for generating the opinion pool will be systematic reviews of the literature on each KQ. Full details of our methods, including search syntax, are in Appendix B of the supplementary materials. Summary opinions will be generated by the GWG based on the results of these systematic reviews.

The second source of information is a qualitative assessment of pivotal guidelines, psychotherapy manuals, book chapters, and articles (Appendix C of the supplementary materials). This allows us to include pivotal literature which will not necessarily be identified in the systematic reviews (e.g., psychotherapy manuals). AP will search these sources to extract a narrative summary of the opinions on each KQ. Quality control will be confirmed by an independent assessor on a random 20% subset of the opinions from these pivotal sources.

The third source of information will be our expert panel. We will invite the expert panelists to provide written opinions on each KQ once the participant has consented to participate. They will be given the results of the systematic reviews and qualitative assessment in tabular form to review before providing their opinions.

For peer review only - http://bmjopen.bmj.com/site/about/guidelines.xhtml

Any evidence synthesis components employed in the process of questionnaire development will follow the relevant recommendations of the Institute of Medicine's Committee on Standards for Systematic Reviews of Comparative Effectiveness Research, especially regarding the qualitative assessment [21].

We anticipate there will be nearly identical opinions for some KQs across the three sources of information. This will lead to significant redundancy which will unnecessarily burden the participants with repeating ratings on nearly identical opinions. For this reason, two independent assessors will thematically group the identical opinions and meet for consensus. After consensus on the groupings is achieved, they will then draft a single summary opinion. This pool of non-redundant opinions will be grouped together under their respective KQs and prepared for circulation to all participants in the form of an online questionnaire hosted on REDCap. The sources of the opinions will be masked to all participants to eliminate bias (e.g., allegiance to a treatment approach).

Phase 2 – Rating evaluation

Participants will be emailed the questionnaire and asked to rate each opinion individually on a 5-point Likert scale of agreement: 1 = strongly disagree, 2 = disagree, 3 = neutral, 4 = agree, and 5 = strongly agree. The questionnaire responses will be de-identified and the opinions will be grouped into their respective KQs. Once all the questionnaires have been received, the ratings will be statistically summarized for each opinion. This will involve calculating and graphically depicting the median, inter-quartile range (IQR), and the percentages in each response category for each opinion, separately for patient/family/support persons and clinician/researcher groups. This statistical summary will be given as feedback to all participants, along with their own rating, so they can see where their ratings are relative to others for each opinion under each KQ.

Phase 3 – Re-rating

Participants will be emailed the same questionnaire and asked to re-rate their agreement on each opinion. This gives participants the opportunity to change their ratings in light of the feedback. After all the questionnaires have been received, the ratings will be statistically analysed using pre-defined consensus thresholds (Table 2).

Opinions meeting threshold for essential, recommended, or no consensus will be brought forward to the consensus meeting for review. If at least one opinion meets one of these thresholds, then we will organize a virtual video meeting. Opinions meeting threshold for exclusion will not be brought to the consensus meeting. If no opinions can be brought to the consensus meeting, then a third round of ratings will be performed. If no opinions can be brought forward after a third round, then the consensus meeting will be held on all the opinions.

Table 2. Consensus thresholds

Essential	The first quartile (Q1) of the ratings for the opinion is > 3 (neutral) in *both* the patient/family/support persons *and* clinician/researcher groups.
Recommended	The first quartile (Q1) of the ratings for the opinion is > 3 (neutral) in *either* the patient/family/support persons *or* clinician/researcher groups.
Exclusion	The third quartile (Q3) of the ratings for the opinion is < 3 (neutral) in *both* the patient/family/support persons *and* clinician/researcher groups.

For peer review only - http://bmjopen.bmj.com/site/about/guidelines.xhtml

No consensus	Essential, recommended, or exclusion thresholds have not been met.

Phase 4 – Moderated consensus meeting

The purpose of this meeting is to finalise the set of opinions and phrasing for each KQ. We will have an independent chair for this meeting to minimise the disadvantages of group discussion by moderating the discussion. The chair will also help identify when significant differences of opinion between patient/family/support persons vs. clinicians/researchers exist. Multiple meetings may take place at the discretion of the chair. The meetings will be recorded for reference if necessary and securely stored. However, all opinions will be considered final at the end of this consensus meeting process. Email correspondence will be allowed during Phase 4 amongst the chair and participants in order to edit phrasing of opinions.

Guideline development

The final opinions from Phase 4 will be the recommendations in the guideline, which will be written up by the GWG. No changes to the opinions will be made by the GWG. If there are significant differences of opinion, the guideline will present the differing recommendations alongside one another.

Conflict of interest management

All members of the GWG and potential participants will submit a conflict of interest (COI) disclosure form, developed based on the International Committee of Medical Journal Editors (ICMJE) form. Potential participants will have to submit this form along with the demographic form in order to be considered for eligibility to participate in the expert panel. COIs will be considered by the study coordinator and, if needed, by the GWG, to determine eligibility. The principle guiding eligibility decisions is to ensure that COIs represent no more than a minority of all study participants. The chair of the consensus meeting will have no COI. Any reported COI will be discussed by the GWG and documented in the guideline in light of its potential impact on the proposed recommendations. This COI data will be collected before consent is obtained to participate in this study. Therefore, if a person is ineligible to participate or doesn't consent to participate in the study after reviewing the consent form, the COI form will be promptly deleted and will not be kept part of the study records.

Statistical analysis

We will analyse the ratings using medians, IQRs, and percentages for each response category. These values will also be graphically depicted in the feedback provided to the participants. We will statistically compare the agreement ratings between patient/family/support persons vs. clinician/researcher groups using independent samples *t*-tests or Wilcoxon rank-sum tests depending on if parametric assumptions are met or not, respectively, and document these differences in the final version of the guideline. All tests of significance will be two-tailed with α set at 0.05.

ETHICS

For peer review only - http://bmjopen.bmj.com/site/about/guidelines.xhtml

This study has received ethics approval by the Hamilton Integrated Research Ethics Board in Hamilton, Ontario, Canada.

DISSEMINATION PLAN

We anticipate that the guideline emerging from this Delphi study will be published in a high-impact peer-reviewed journal. The guideline will be disseminated to regional, national, and international audiences through presentations at regional, national, and international conferences. Finally, we will disseminate the guideline through the national and international associations for psychiatry, psychology, emergency medicine, nursing, and social work.

DISCUSSION

Clinicians caring for adults with BPD in acute settings such as the ED have little guidance or evidence to base their decisions on. In this vacuum, patients are at risk of a wide variety of treatment approaches and practices, ranging from excellent to iatrogenic. Therefore, specific and detailed guidance for managing adults with BPD in the ED is urgently needed for the sake of patients and their families/support persons. Consensus methods such as Delphi play an important role in science where there is limited or contradictory evidence [16]. Through a structured process, these methods assess the degree of agreement and/or resolve disagreement amongst experts. The consensus can provide helpful scaffolding for clinical guidelines until empirical study fills the gaps. Furthermore, the clinical guidelines will provide important hypotheses for these empirical investigations. This is the rationale for our Delphi study. The opinions emerging from this study will become the first consensus guideline on the management of adults with BPD in the ED.

The nominal group technique is another consensus method used in health research.[15] It uses a facilitated meeting of experts who gather information, rate, discuss, and re-rate/discuss a series of items, questions, or opinions. Each participant contributes their ideas to the facilitator who thematically groups the ideas for discussion. The ideas are then rated, summarized, and the summary is fed back to the participants for discussion and re-rating. The final ratings are then summarized and evaluated for consensus using pre-defined thresholds. The disadvantage of this technique is that it is often not feasible for all relevant experts to participate in the meeting. This is why typically the nominal group technique usually involves small groups of 9-12 individuals [16]. Moreover, experts often do not have the time to devote to these steps commensurate with the complexity of the issues. By contrast, the Delphi method allows researchers to synthesize a large amount of information across many individuals with relatively minimal imposition on the participants' time.

The main limitation of this study is that, even if we achieve consensus on some or all opinions, this does not guarantee that the correct recommendations have been found. Consensus does not necessarily imply truth. Only empirical studies can validate the opinions generated from a consensus process, which at this time are lacking. Therefore, our study is best viewed as a structured sampling of expert opinions rather than an attempt to reach a definitive conclusion about what are truly the best practices in managing adults with BPD in the ED. Another limitation is that our expert panel will not be representative of the disciplines, backgrounds, and treatment approaches we are sampling. Relatedly, we anticipate there will be more

For peer review only - http://bmjopen.bmj.com/site/about/guidelines.xhtml

For peer review only

clinician/researcher participants on the panel, which risks silencing patient/family/support persons voices. This risk will be mitigated by documenting and discussing the recommendations of patient/family/support persons if significant differences of opinion exist. Additionally, this study does not focus on guidelines for pediatric patients with BPD. While many of the guidelines here will be relevant for pediatric patients, a logical next step would be to develop guidelines for that population, given the inherent differences in the assessment and management of adults versus youth. Notwithstanding these limitations, we hope the guidance emerging from this Delphi study will be a helpful clinical resource to improve the care of adults with BPD in the ED.

For peer review only

For peer review only - http://bmjopen.bmj.com/site/about/guidelines.xhtml

Contributors

AP, VH, BH, DF, JP, PR, BF, and PL are responsible for the design of the study and early drafts of the protocol. PL is the principal investigator. AP is the study coordinator and wrote the first draft of the protocol. AP, VH, BH, and PL are responsible for final draft of the protocol manuscript. All authors have read and approved the final manuscript, provided final approval for publication, and agree to be accountable for all aspects of this work. AP attests all listed authors meet authorship criteria and that no others meeting criteria have been omitted.

Funding

This research was funded in part by National Science Centre, Poland (grant number 2021/41/B/HS6/02844). For the purpose of Open Access, the author has applied a CC-BY public copyright licence to any Author Accepted Manuscript (AAM) version arising from this submission.

Competing interests

PL is a co-developer of GPM and VH published an expert opinion on the management of BPD in the ED based on GPM principles.

Acknowledgement

We would like to express our gratitude to the people with BPD and their family members/support persons for reviewing and commenting on an earlier draft of this protocol, as well as providing advice on the key questions for the guideline. We would also like to thank the patients with BPD who we have cared for and learned from over the years. Their lives inspired this project.

For peer review only

For peer review only - http://bmjopen.bmj.com/site/about/guidelines.xhtml

Figure legends

Figure 1. Analytic framework and key questions (KQs).

For peer review only

For peer review only - http://bmjopen.bmj.com/site/about/guidelines.xhtml

REFERENCES

1. Gunderson, J. and P. Links, *Handbook of Good Psychiatric Management for Borderline Personality Disorder*. 2014, Washington, DC: American Psychiatric Association Publishing.
2. Livesley, J., *Integrated Treatment for Borderline Personality Disorder: A Practical Guide to Combining Effective Treatment Methods*. 2017, Cambridge, UK: Cambridge University Press.
3. APA, *Diagnostic and Statistical Manual of Mental Disorders*. 5th ed. 2013, Washington, DC: Author.
4. Cristea, I.A., et al., *Efficacy of Psychotherapies for Borderline Personality Disorder: A Systematic Review and Meta-analysis.* JAMA Psychiatry, 2017. **74**(4): p. 319-328.
5. Lieb, K., et al., *Pharmacotherapy for borderline personality disorder: Cochrane systematic review of randomised trials.* The British Journal of Psychiatry, 2010. **196**(1): p. 4-12.
6. Stoffers-Winterling, J., O.J. Storebø, and K. Lieb, *Pharmacotherapy for borderline personality disorder: an update of published, unpublished and ongoing studies.* Current psychiatry reports, 2020. **22**: p. 1-10.
7. Health, N.C.C.f.M., *Borderline Personality Disorder: The NICE Guideline on Treatment and Management. National Clinical Practice Guideline No. 78*. 2009, The British Psychological Society and The Royal College of Psychiatrists.
8. Association, A.P., *Practice guideline for the treatment of patients with borderline personality disorder*. 2001: American Psychiatric Association Practice Guidelines.
9. Smith, M., et al., *Clinical practice guideline for the management of borderline personality disorder.* 2012, National Health and Medical Research Council (NHMRC): Melbourne.
10. Pascual, J.C., et al., *Hospitalization and Pharmacotherapy for Borderline Personality Disorder in a Psychiatric Emergency Service.* Psychiatric Services, 2007. **58**(9): p. 1199-1204.
11. Hong, V., *Borderline personality disorder in the emergency department: good psychiatric management.* Harvard Review of Psychiatry, 2016. **24**(5): p. 357-366.
12. Gabbard, G.O. and S.M. Wilkinson, *Management of countertransference with borderline patients*. 2000, Northvale, New Jersey: Jason Aronson Inc.
13. Shah, R. and M.C. Zanarini, *Comorbidity of Borderline Personality Disorder: Current Status and Future Directions.* Psychiatric Clinics of North America, 2018. **41**(4): p. 583-593.
14. Nelson, H.D., et al., *Screening for Osteoporosis: An Update for the U.S. Preventive Services Task Force.* Annals of Internal Medicine, 2010. **153**(2): p. 99-111.
15. Powell, C., *The Delphi technique: myths and realities.* Journal of Advanced Nursing, 2003. **41**(4): p. 376-382.
16. Jones, J. and D. Hunter, *Qualitative Research: Consensus methods for medical and health services research.* BMJ, 1995. **311**(7001): p. 376-380.
17. Gunderson, J. and P. Links, *Borderline Personality Disorder: A Clinical Guide*. 2008, Washington, DC: American Psychiatric Association Publishing.
18. Lau, J., *Models of interaction between clinical practice guidelines (CPG) groups and systematic review (SR) teams*, in *IOM Committee on Standards for Developing*

For peer review only - http://bmjopen.bmj.com/site/about/guidelines.xhtml

Trustworthy Clinical Practice Guidelines meeting (January 12, 2010). 2010: Washington, DC.
19. Armstrong, M.J., et al., *Framework for enhancing clinical practice guidelines through continuous patient engagement.* Health Expectations, 2017. **20**(1): p. 3-10.
20. Excellence, N.I.f.H.a.C., *The Guidelines Manual*. 2012, National Institute for Health and Clinical Excellence (NICE).
21. Eden, J., et al., *Finding What Works in Health Care: Standards for Systematic Reviews*. 2011, Washington, DC: Institute of Medicine (US) Committee on Standards for Systematic Reviews of Comparative Effectiveness Research, National Academies Press.

For peer review only

For peer review only - http://bmjopen.bmj.com/site/about/guidelines.xhtml

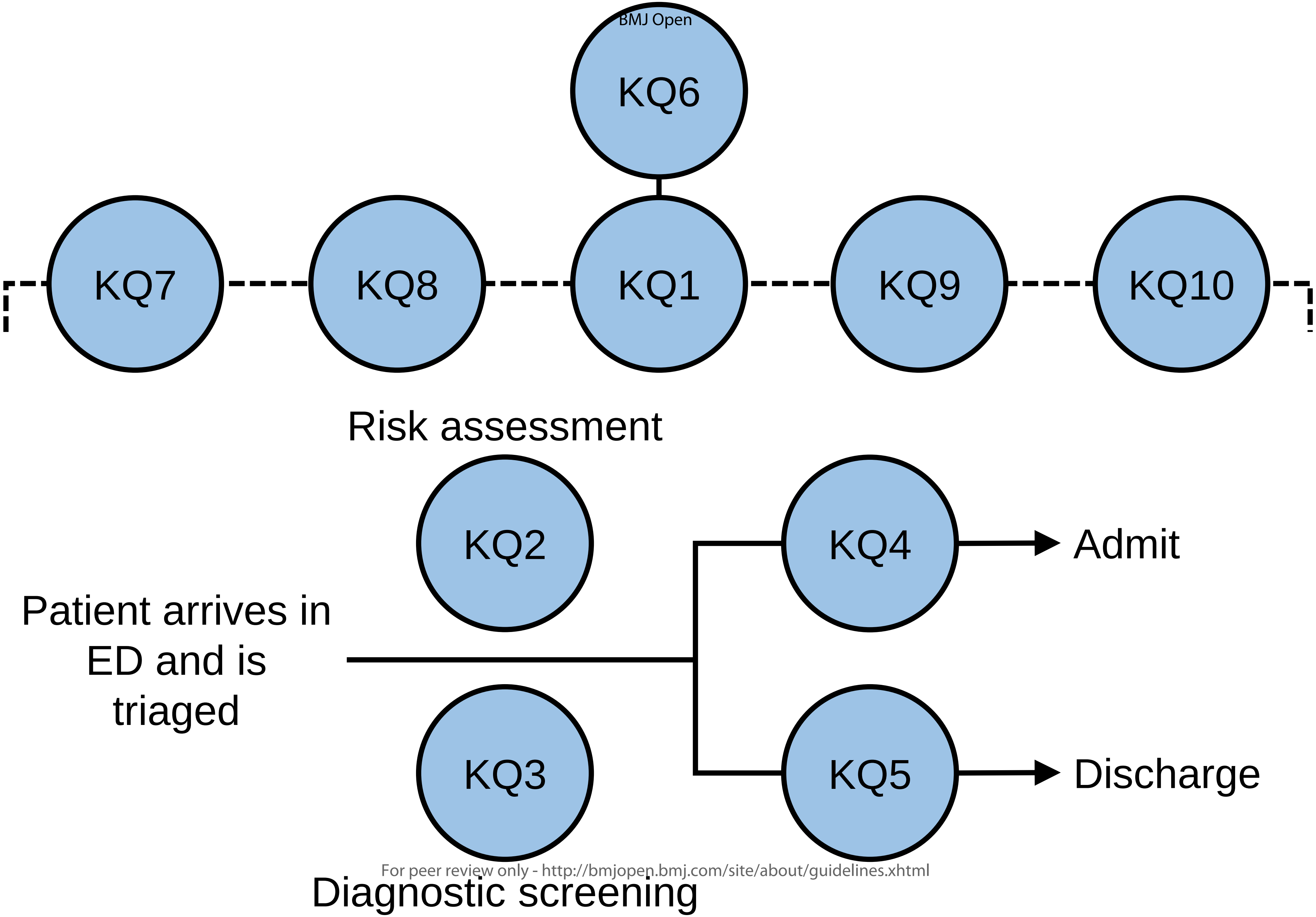
KQ6
KQ7
KQ8
KQ1
KQ9
KQ10
Risk assessment
KQ2
KQ4
Admit
Patient arrives in ED and is triaged
KQ3
KQ5
Discharge
Diagnostic screening

Supplementary Materials

Protocol for a Delphi consensus study to develop guidelines for the management of adults with borderline personality disorder in the emergency department

For peer review only

For peer review only - http://bmjopen.bmj.com/site/about/guidelines.xhtml

APPENDIX A

Additional study development and design information.

Patient and public involvement

While developing this protocol and the KQs included here, we sought advice from people with BPD and their family members/support persons on what are the most important questions to include from their perspective as well as feedback on an earlier draft of the protocol. VH contacted potential participants by utilizing the listserve from several advocacy organizations, and 13 provided constructive feedback including 3 persons with BPD and 9 family members and 1 support person of someone with BPD. Participants were asked to review the KQs, provide feedback, and propose other KQs. All 13 participants were those who themselves or had supported a person who had experience with an emergency department presentation for BPD-related issues. They agreed on the set of proposed KQs and 4 participants included specific suggestions. One person with BPD wanted KQ8 to be explored further. Family members and the support person requested additional KQs and edits, all of them were applied after consultation with the GWG and are listed here: (1) two additional KQ (9 and 10) were added, (2) the order of questions was changed (KQ4 became KQ1 and the numbering of the others was adjusted accordingly), (3) "support person" was added to KQ7 and the word “aggression” to KQ6. We have also edited the methods of the guideline development process below to include the wider support circle of people with BPD (going beyond family members or caregivers). 10 others expressed interest in participating in this phase of the project. 1 did not meet criteria because they did not have BPD, 1 ultimately did not choose to participate, and 8 did not respond to the protocol when it was shared. Additional feedback included preferences relating to the need to consider biases based on social-economic-status, race and gender as well during the guideline development process. Their preferences will be included in the Delphi process and shared with the guideline development team. Moreover, special attention will be given to these biases while preparing the systematic review. The guidelines will be shared with the participants in the development of guidelines.

Risk of bias mitigation

PL and VH will be participants in the Delphi process given their recognized expertise. This means there is a potential source of bias due to their involvement in developing GPM[1 2] and GPM applications to ED settings.[3] This will be mitigated by ensuring they have no access to the data or involvement in the data analysis. The chair of the consensus meeting will have no affiliation with a treatment approach.

For peer review only - http://bmjopen.bmj.com/site/about/guidelines.xhtml

APPENDIX B

Protocol for systematic reviews

1. Review questions

We will conduct ten systematic reviews to search the literature for articles on the following key questions (KQs) related to the key areas of care of adults with borderline personality disorder (BPD) in the emergency department (ED):

1. What psychosocial and pharmacological interventions in the ED are associated with benefit while minimising harm for adults with BPD, with considerations regarding potential comorbidities?
    a. What interventions in the ED are effective?
    b. What interventions in the ED are harmful?
2. What are the effective approaches to assess and manage the risk of suicide and self-harm of adults with BPD, with considerations regarding potential comorbidities?
3. What should be included as part of the emergency evaluation of adults to screen for previously undiagnosed BPD?
4. What is the role of hospitalisation in managing adults with BPD?
    a. What are the indications for hospitalisation?
    b. What is the effectiveness of hospitalisation?
    c. What are the harms of hospitalisation?
5. What psychosocial aftercare services (including partial hospitalisation) can help adults with BPD who present to the ED but are not hospitalised?
6. What interventions in the ED for agitation and aggression are associated with benefit while minimising harm for adults with BPD?
    a. What interventions for agitation and aggression are effective?
    b. What interventions for agitation and aggression are harmful?
7. What are effective approaches to involving family members and support persons in the care of adults with BPD in the ED?
8. What are effective approaches to involving outpatient providers in the care of adults with BPD in the ED?
9. What are effective approaches to providing optimal medical care for adults with BPD presenting in a mental health crisis to the ED ?
10. What are the most effective environmental elements (physical layout and interior design) for adults with BPD in the ED?

2. Searches

The following databases will be searched from inception: MEDLINE, MEDLINE In-Process, Embase, Global Health, and PsychInfo with no language restrictions. The results of the individual databases will be transferred into a reference manager program and duplicates will be eliminated. To search for additional reports, we will search the reference lists of the included studies. A PRISMA flow chart will be included in the final publication.

For peer review only - http://bmjopen.bmj.com/site/about/guidelines.xhtml

3. Search strategy

KQ1

1. ((borderline$ or cluster$) adj3 personalit$).mp
2. (borderline$ or BPD).mp
3. ((difficult$ or challeng$) adj3 patient$).mp
4. (recurrent$ or repeat$) adj3 (suicid$ or self-harm$ or self-injur$ or self-cut$ or mutilat$).mp
5. or/1-4
6. (emergency adj3 (department$ or room$ or treatment$ or medicine$ or health service$)).mp
7. 5 and 6

KQ2

1. ((borderline$ or cluster$) adj3 personalit$).mp
2. (borderline$ or BPD).mp
3. ((difficult$ or challeng$) adj3 patient$).mp
4. (recurrent$ or repeat$) adj3 (suicid$ or self-harm$ or self-injur$ or self-cut$ or mutilat$).mp
5. or/1-4
6. ((suicid$ or self harm$ or self injur$ or self cut$ or mutilat$ or NSSI or DSH) and (risk$ adj5 (assess$ or evaluat$))).mp
7. 5 and 6

KQ3

1. ((borderline$ or cluster$) adj3 personalit$).mp
2. (borderline$ or BPD).mp
3. ((difficult$ or challeng$) adj3 patient$).mp
4. (recurrent$ or repeat$) adj3 (suicid$ or self-harm$ or self-injur$ or self-cut$ or mutilat$).mp
5. or/1-4
6. (assessment$ or screening$ or diagnostic$).mp
7. 5 and 6

KQ4

1. ((borderline$ or cluster$) adj3 personalit$).mp
2. (borderline$ or BPD).mp
3. ((difficult$ or challeng$) adj3 patient$).mp
4. (recurrent$ or repeat$) adj3 (suicid$ or self-harm$ or self-injur$ or self-cut$ or mutilat$).mp
5. or/1-4
6. (hospitalization$ or hospitalisation$ or admit$ or admission$).mp
7. (((single$ or double$ or trebl$ or tripl$) adj5 (blind$ or mask$)) or trial$ or (clinic$ adj2 trial$) or open-label$ or openlabel$ or (pre$ adj5 post$) or (pretest adj5 posttest) or cohort or (program$ adj5 evaluate$) or ((assurance$ or improv$ or indicator$ or measure$ or metric$) adj2 quality)).mp

For peer review only

For peer review only - http://bmjopen.bmj.com/site/about/guidelines.xhtml

8. 5 and 6 and 7

KQ5

1. ((borderline$ or cluster$) adj3 personalit$).mp
2. (borderline$ or BPD).mp
3. ((difficult$ or challeng$) adj3 patient$).mp
4. (recurrent$ or repeat$) adj3 (suicid$ or self-harm$ or self-injur$ or self-cut$ or mutilat$).mp
5. or/1-4
6. (emergency adj3 (department$ or room$ or treatment$ or medicine$ or health service$)).mp
7. (aftercare$ or (discharge adj5 (plan$ or service$ or manag$ or treatment$ or therap$))).mp
8. 5 and 6 and 7

KQ6

1. ((borderline$ or cluster$) adj3 personalit$).mp
2. (borderline$ or BPD).mp
3. ((difficult$ or challeng$) adj3 patient$).mp
4. (recurrent$ or repeat$) adj3 (suicid$ or self-harm$ or self-injur$ or self-cut$ or mutilat$).mp
5. or/1-4
6. (agitat$ or aggress$ or violen$ or anxiet$ or anxious$).mp
7. (((single$ or double$ or trebl$ or tripl$) adj5 (blind$ or mask$)) or trial$ or (clinic$ adj2 trial$) or open-label$ or openlabel$ or (pre$ adj5 post$) or (pretest adj5 posttest) or cohort or (program$ adj5 evaluate$) or ((assurance$ or improv$ or indicator$ or measure$ or metric$) adj2 quality)).mp
8. (emergency adj3 (department$ or room$ or treatment$ or medicine$ or health service$)).mp
9. 5 and 6 and 9 and 8

KQ7

1. ((borderline$ or cluster$) adj3 personalit$).mp
2. (borderline$ or BPD).mp
3. ((difficult$ or challeng$) adj3 patient$).mp
4. (recurrent$ or repeat$) adj3 (suicid$ or self-harm$ or self-injur$ or self-cut$ or mutilat$).mp
5. or/1-4
6. (emergency adj3 (department$ or room$ or treatment$ or medicine$ or health service$)).mp
7. (famil$ or partner$ or caregiver$ or parent$ or guardian$ or children$ or support$).mp
8. 5 and 6 and 7

KQ8

1. ((borderline$ or cluster$) adj3 personalit$).mp
2. (borderline$ or BPD).mp

For peer review only

3. ((difficult$ or challeng$) adj3 patient$).mp
4. (recurrent$ or repeat$) adj3 (suicid$ or self-harm$ or self-injur$ or self-cut$ or mutilat$).mp
5. or/1-4
6. (emergency adj3 (department$ or room$ or treatment$ or medicine$ or health service$)).mp
7. ((outpatient$ or ambulator$) adj5 (provider$ or team$ or caregiver$ or treater$ or physician$ or psychiatrist$ or psychologist$)).mp
8. 5 and 6 and 7

KQ9
1. ((borderline$ or cluster$) adj3 personalit$).mp
2. (borderline$ or BPD).mp
3. ((difficult$ or challeng$) adj3 patient$).mp
4. (recurrent$ or repeat$) adj3 (suicid$ or self-harm$ or self-injur$ or self-cut$ or mutilat$).mp
5. or/1-4
6. (emergency adj3 (department$ or room$ or treatment$ or medicine$ or health service$)).mp
7. ((medical$ or physical health$) adj3 (care$ or treatment$ or management$)).mp
8. 5 and 6 and 7

KQ10
1. ((borderline$ or cluster$) adj3 personalit$).mp
2. (borderline$ or BPD).mp
3. ((difficult$ or challeng$) adj3 patient$).mp
4. (recurrent$ or repeat$) adj3 (suicid$ or self-harm$ or self-injur$ or self-cut$ or mutilat$).mp
5. or/1-4
6. (emergency adj3 (department$ or room$ or treatment$ or medicine$ or health service$)).mp
7. (environment$ or architecture$ or design$ or layout$ or decor or ergonomic$).mp
8. 5 and 6 and 7

4. Condition or domain being studied

We will study the key areas of emergency care of adults with BPD defined by our KQs.

5. Article inclusion/exclusion criteria

Inclusion: the report is (1) a primary study (clinical trial, cohort study, cross-sectional study, pre/post evaluation, program evaluation, or quality assurance), guideline, standards, consensus statement, decision-making tool, assessment/diagnostic/screening tool, or expert opinion, (2) includes a sample of at least >75% adults with BPD in an emergency setting (if a primary study) or is about adults with BPD in emergency settings (if a guideline, standard, consensus statement, decision-making tool, assessment/diagnostic/screening tool, or expert opinion), and is about

For peer review only - http://bmjopen.bmj.com/site/about/guidelines.xhtml

either (i) the overall care of adults with BPD in the ED using psychosocial and/or pharmacological interventions (KQ1), (ii) suicide/self-harm risk assessment and/or management of adults with BPD (KQ2), (iii) diagnostic screening for BPD, (iv) hospitalisation of adults with BPD (KQ4), (v) psychosocial aftercare services (including partial hospitalisation) of adults with BPD who present to the ED but are not hospitalised (KQ5), (vi) the pharmacological and/or non-pharmacological management of agitation/aggression in adults with BPD (KQ6), (vii) approaches to involving family members/support persons in the care of adults with BPD in the ED (KQ7), (viii) approaches to involving outpatient providers in the care of adults with BPD in the ED (KQ8), (ix) general medical care of adults with BPD presenting to the ED in a mental health crisis (KQ9), and (x) environmental elements (physical layout and interior design) of the ED in relation to adults with BPD presenting to the ED in a mental health crisis (KQ10).
Exclusion: none.

6. Main outcome(s)

The primary outcome for the primary studies is efficacy which will be categorized as follows. These outcomes will be summarized narratively along with an effect size if it can be calculated from the report. If an effect size cannot be calculated from the report, the outcome will be narratively summarized only.

1. *Suicidality outcomes*
   i. Self-harm
   ii. Suicidal ideation
   iii. Suicide attempt
   iv. Death by suicide
2. *BPD-related outcomes*
   i. Emotion dysregulation
   ii. Depression
   iii. Anxiety
   iv. Anger/irritability
   v. Aggression
   vi. Paranoia
   vii. Dissociation
   viii. Interpersonal dysfunction
   ix. Self dysfunction (e.g., identity disturbance)
3. *Global outcomes*
   i. Quality of life
   ii. Global psychosocial functioning
   iii. Global symptom severity
4. *Service utilization outcomes*
   i. Patient satisfaction
   ii. Hospitalisation
   iii. ED visits
   iv. Adherence with medications
   v. Adherence with appointments.
   vi. Time to all-cause treatment discontinuation

For peer review only - http://bmjopen.bmj.com/site/about/guidelines.xhtml

We will exclude measures when they cannot be categorized. Note, suicidal ideation, planning, and behaviour (including self-harm) will be always coded under behavioural dyscontrol, even if in the report it may have been coded as a tolerability outcome. The primary outcome for non-primary studies (i.e., guideline, standards, consensus statement, decision-making tool, assessment/diagnostic/screening tool, or expert opinions) will be a narrative summary of the opinion on a given KQ.

Measures of effect: See Additional outcomes section below.

7. Additional outcome(s)

The secondary outcome for the primary studies is tolerability which will be categorized as follows. These outcomes will be summarized narratively along with an effect size if it can be calculated from the report. If an effect size cannot be calculated from the report, the outcome will be narratively summarized only. There will be no secondary outcomes for non-primary studies.

- Any adverse event
- Serious adverse event
- Death (non-suicide)
- Nausea
- Constipation
- Diarrhea
- Dry mouth
- Headache
- Dizziness
- Sedation
- Sleep disturbance
- Restlessness/agitation
- Menstrual changes
- Increased appetite
- Weight gain
- Decreased appetite
- Weight loss
- Extrapyramidal symptoms
- Akathisia
- Tardive dyskinesia
- Tremor (any type)
- Any other neurological dysfunction
- Cognitive dysfunction
- Sexual dysfunction
- Systolic blood pressure
- Diastolic blood pressure
- QTc interval
- Liver enzymes (AST, ALT, ALP, or GGT)
- Bilirubin (total, direct, or indirect)
- Albumin
- International normalized ratio (INR)
- Low-density lipoprotein (LDL)
- High-density lipoprotein (HDL)
- Triglycerides
- HbA1C
- Leukocyte count
- Hemoglobin
- Platelet count
- Creatinine
- Urea or blood urea nitrogen (BUN)
- Estimated glomerular filtration rate
- Sodium
- Potassium
- Calcium
- Phosphate
- Magnesium
- Creatine kinase
- Prolactin
- Thyroid stimulating hormone
- Thyroid hormone

For peer review only - http://bmjopen.bmj.com/site/about/guidelines.xhtml

- Weight loss
- Endpoint weight
- Total cholesterol
- Fasting blood glucose

Measures of effect:

For each primary study we will calculate the standardized mean difference (SMD, Hedges' *g*) for continuous measures (efficacy and tolerability data). If the study includes a comparator, we will use endpoint data between the active intervention vs. control intervention to calculate the SMD. If the study does not include a comparator, we will use pre vs. post data to calculate the SMD. If only change scores are reported, we will use change scores to calculate the SMD between the active intervention vs. control intervention. If both are reported, endpoint scores will be used. Dichotomous outcomes measured throughout the study and reported at as totals at endpoint (e.g., total number of self-harm episodes during the trial) will be considered endpoint data. Intention-to-treat (ITT) data will be used whenever available. Negative SMDs will indicate the active intervention was more beneficial than control intervention. The relative risk (RR) will be calculated for dichotomous tolerability data only. RR less than 1 will indicate the active intervention had less adverse effects than control intervention. Effect sizes of dichotomous efficacy data will be converted to the SMD using the procedure discussed shortly.

Combining multiple effect sizes within trials:

If a study reports multiple outcomes (efficacy or tolerability) per category, the effect sizes will be combined to produce a single composite effect size (SMD or RR) using the method in Borenstein et al (2009).[4] We will assume a within-cluster correlation between outcomes of $r = 0.45$. We will make this assumption because studies almost never report the inter-correlations between their outcome measures. $r = 0.45$ is the 95th percentile correlation across 708 meta-analytically-derived correlations in psychology between different constructs.[5] We will use the 95th percentile since it gives a more conservative estimate of the correlation between similar outcomes within studies. This is because smaller correlations lower the variance of combined effect sizes.[4]

If a continuous outcome is reported alongside its dichotomous outcome (e.g., response rate based on ≥ 50% reduction in the scale), only the continuous outcome will be extracted. This is due to the redundancy and the lower statistical power of dichotomized continuous scales. In order to combine continuous (e.g., self-report scale of impulsivity) and dichotomous (e.g., number of aggressive episodes) efficacy data within a symptom category (e.g., behavioural dyscontrol) we will perform the following procedure: an odds ratio (OR) will be calculated for the dichotomous outcome and converted to Cohen's *d* using established equations.[4] We will apply the *J* correction factor to convert the Cohen's *d* to Hedges' *g* (the SMD).[5] This will be combined with the SMD of the continuous outcome(s) within the symptom category. Note, the RR is preferred over the OR for RCTs and cohort studies. The OR approximates the RR when the incidence of the outcome is low (< 10%). As the incidence increases, the OR overestimates the RR when it is greater than 1 and underestimates the RR when it's less than 1.[6] To the best of our knowledge there are no established procedures to convert RRs directly to SMDs. Therefore, while imperfect, we will use this procedure with dichotomous outcomes given the advantages of combining multiple outcomes into a single effect size per symptom category.

For peer review only - http://bmjopen.bmj.com/site/about/guidelines.xhtml

8. Data extraction (selection and coding)

1. **Selection of studies:** two reviewers will independently screen the titles and abstracts of the search results to determine potential eligibility. Full-text reports of the potentially eligible reports will then be retrieved and assessed by two reviewers using our inclusion/exclusion criteria to determine eligibility. Disagreements at either the screening or full-text review stages of the selection phase will be resolved through consensus between the two reviewers and, if necessary, a third reviewer. Decisions regarding eligibility will be documented.
2. **Data extraction:** Two reviewers will independently extract the main outcomes and additional outcomes from the reports into standardized electronic databases. Discrepancies in data extraction will be resolved through consensus between the two authors and, if necessary, a third author.

9. Risk of bias assessment

Risk of bias in the randomized controlled trials (RCTs) will be assessed using the Cochrane Risk of Bias 2 (ROB 2). Risk of bias in the non-randomized primary studies will be assessed using the Cochrane Risk of Bias in Non-randomized Studies of Interventions (ROBINS-I). Risk of bias will be assessed by two independent reviewers. All disagreements will be resolved through consensus between the two reviewers and a third reviewer if necessary. ROBINS-I requires listing in the protocol the confounding domains and co-interventions. Confounding domains and co-interventions are those for which adjustment is expected to lead to a clinically important change in the estimated effect of the intervention. We defined the following:

1. **Confounding domains:** age, sex, race/ethnicity, comorbidities, socioeconomic status.
2. **Co-interventions:** concurrent psychotherapy or pharmacotherapy.

10. Strategy for data synthesis

The characteristics of the included primary studies will be summarized in a tabular form using the PICO format along with a narrative summary of the results with the effect sizes of the efficacy/tolerability data when available. When appropriate, quality of evidence will be assessed using the GRADE approach.[7] The narrative summaries of the opinions from the non-primary studies will be summarized in tabular form.

For peer review only - http://bmjopen.bmj.com/site/about/guidelines.xhtml

APPENDIX C

The following pivotal guidelines, psychotherapy manuals, book chapters, and articles will be reviewed as part of the qualitative assessment in order to extract opinions relevant to our key questions.

Guidelines

1. National Institute for Health and Clinical Excellence (NICE) guideline on treatment and management: Borderline personality disorder[8]
2. American Psychiatric Association (APA) practice guideline for the treatment of patients with borderline personality disorder[9]
3. National Health and Medical Research Council (NHMRC) clinical practice guideline for the management of borderline personality disorder[10]
4. World Federation of Societies of Biological Psychiatry (WFSBP) Guidelines for biological treatment of personality disorders[11]
5. The project BETA guidelines[12]
6. Garriga et al (2016) Assessment and management of agitation in psychiatry: Expert consensus. *The World Journal of Biological Psychiatry*.[13]

Psychotherapy manuals

1. Linehan (1993) Cognitive-behavioral treatment of borderline personality disorder.[14]
2. Bateman & Fonagy (2016) Mentalization-based treatment for personality disorders: A practical guide.[15]
3. Yeomans et al (2015) Transference focused psychotherapy for borderline personality disorder: A Clinical Guide.[16]
4. Kernberg (1984) Severe personality disorders: Psychotherapeutic Strategies.[17]
5. Gunderson & Links (2008) Borderline personality disorder: A clinical guide.[1]
6. Gunderson & Links (2014) Handbook of good psychiatric management for borderline personality disorder.[2]
7. Arntz & van Genderen (2021) Schema therapy for borderline personality disorder, 2nd Ed.[18]
8. Davidson (2008) Cognitive therapy for personality disorders: A guide for clinicians, 2nd Ed.[19]
9. Black & Blum (2016) Systems training for emotional predictability and problem solving for borderline personality disorder: Implementing STEPPS around the globe.[20]
10. Dawson & MacMillan (1993) Relationship management of the borderline patient: From understanding to treatment.[21]
11. Livesley (2003) Practical management of personality disorder.[22]
12. Livesley (2017) Integrated modular treatment for borderline personality disorder: A practical guide to combining effective treatment methods.[23]

Other books

1. Gabbard (2014) Psychodynamic psychiatry in clinical practice, 5th Ed.[24]
2. Gabbard & Wilkinson (2000) Management of countertransference with borderline patients.[25]

For peer review only - http://bmjopen.bmj.com/site/about/guidelines.xhtml

3. Choi-Kain & Gunderson (2019) Applications of good psychiatric management for borderline personality disorder.[26]
4. Sonley & Choi-Kain (2020) Good psychiatric management and dialectical behavior therapy: A clinician's guide to integration and stepped care.[27]

Book chapters

1. Hong & Casher (2020) The inpatient with borderline personality disorder. In: *Manual of Inpatient Psychiatry, 2nd Ed*.[28]
2. Hong et al (2020) Personality disorders. In: *Emergency Psychiatry: Principles and Practice, 2nd Ed*.[29]
3. Hong et al (2019) Traitements dans les services d'urgence. In: *Trouble de la Personnalite Borderline: Pratiques Therapeutiques*.[30]
4. Curry & Riddle (2021) Personality disorders in the emergency department. In: *Behavioral Emergencies for Healthcare Providers*.[31]

Published papers

1. Hong (2016) Borderline personality disorder in the emergency department: good psychiatric management. *Harvard Review of Psychiatry*.[3]
2. Hong et al (2019) Antisocial and borderline personality disorders in the emergency department: conceptualizing and managing "Malingered" or "Exaggerated" symptoms. *Current Behavioral Neuroscience Reports*.[32]
3. Shaikh et al (2017). Patients with borderline personality disorder in emergency departments. *Frontiers in Psychiatry*.[33]
4. Broadbear et al (2022). Emergency department utilisation by patients with a diagnosis of borderline personality disorder: An acute response to a chronic disorder. *Emergency Medicine Australasia*.[34]
5. Cases et al (2020). Evidence of practice gaps in emergency psychiatric care for borderline personality disorder: how can this be explained? *BMC Psychiatry*.[35]
6. Acres et al (2021). From the community to the emergency department: A study of hospital emergency department nursing practices from the perspective of carers of a loved one with Borderline Personality Disorder. *Health & social care in the community*.[36]
7. Paris (2002) Chronic suicidality among patients with borderline personality disorder. *Psychiatric Services*.[37]
8. Paris (2004) Understanding self-mutilation in borderline personality disorder. *Harvard Review of Psychiatry*.[38]
9. Paris (2004) Half in love with easeful death: The meaning of chronic suicidality in borderline personality disorder. *Harvard Review of Psychiatry*.[39]
10. Paris (2004) Is hospitalization useful for suicidal patients with borderline personality disorder? *Journal of Personality Disorders*.[40]
11. Paris (2006) Predicting and preventing suicide: Do we know enough to do either? . *Harvard Review of Psychiatry*.[41]
12. Paris (2021) Can we predict or prevent suicide?: An update. *Preventative Medicine*.[42]
13. Goodman et al (2012) Suicidal risk and management in borderline personality disorder. *Current Psychiatry Reports*.[43]

For peer review only - http://bmjopen.bmj.com/site/about/guidelines.xhtml

References

1. Gunderson J, Links P. Borderline Personality Disorder: A Clinical Guide. Washington, DC: American Psychiatric Association Publishing 2008.
2. Gunderson J, Links P. Handbook of Good Psychiatric Management for Borderline Personality Disorder. Washington, DC: American Psychiatric Association Publishing 2014.
3. Hong V. Borderline personality disorder in the emergency department: good psychiatric management. *Harv Rev Psychiatry* 2016;24(5):357-66. doi: 10.1097/HRP.0000000000000112
4. Borenstein M, Hedges LV, Higgins JP, et al. Introduction to Meta-analysis. Chichester, UK: Wiley 2009.
5. Gignac GE, Szodorai ET. Effect size guidelines for individual differences researchers. *Personality and individual differences* 2016;102:74-78.
6. Zhang J, Kai FY. What's the relative risk?: A method of correcting the odds ratio in cohort studies of common outcomes. *JAMA* 1998;280(19):1690-91.
7. Guyatt GH, Oxman AD, Vist GE, et al. GRADE: an emerging consensus on rating quality of evidence and strength of recommendations. *BMJ* 2008;336(7650):924. doi: 10.1136/bmj.39489.470347.AD
8. Health NCCfM. Borderline Personality Disorder: The NICE Guideline on Treatment and Management. National Clinical Practice Guideline No. 78: The British Psychological Society and The Royal College of Psychiatrists, 2009.
9. Association AP. Practice guideline for the treatment of patients with borderline personality disorder: American Psychiatric Association Practice Guidelines 2001.
10. Smith M, Chanen A, Ford F, et al. Clinical practice guideline for the management of borderline personality disorder. Melbourne: National Health and Medical Research Council (NHMRC), 2012.
11. Herpertz SC, Zanarini M, Schulz CS, et al. World Federation of Societies of Biological Psychiatry (WFSBP) guidelines for biological treatment of personality disorders. *The World Journal of Biological Psychiatry* 2007;8(4):212-44.
12. Holloman Jr GH, Zeller SL. Overview of Project BETA: best practices in evaluation and treatment of agitation. *Western Journal of Emergency Medicine* 2012;13(1):1.
13. Garriga M, Pacchiarotti I, Kasper S, et al. Assessment and management of agitation in psychiatry: Expert consensus. *The World Journal of Biological Psychiatry* 2016;17(2):86-128. doi: 10.3109/15622975.2015.1132007
14. Linehan M. Cognitive-behavioral Treatment of Borderline Personality Disorder. New York City, NY: Guilford Press 1993.
15. Bateman A, Fonagy P. Mentalization based treatment for personality disorders: A practical guide. Oxford, UK: Oxford University Press 2016.
16. Yeomans FE, Clarkin JF, Kernberg OF. Transference-Focused Psychotherapy for Borderline Personality Disorder: A Clinical Guide. Arlington, VA: American Psychiatric Association Publishing 2015.
17. Kernberg OF. Severe Personality Disorders: Psychotherapeutic Strategies. New Haven, CT: Yale University Press 1984.
18. Arntz A, Van Genderen H. Schema therapy for borderline personality disorder. West Sussex, UK: John Wiley & Sons, Ltd 2021.
19. Davidson K. Cognitive therapy for personality disorders: A guide for clinicians. 2nd ed. East Sussex, UK: Routledge 2008.

For peer review only

For peer review only - http://bmjopen.bmj.com/site/about/guidelines.xhtml

20. Black DW, Blum N. Systems training for emotional predictability and problem solving for borderline personality disorder: Implementing STEPPS around the globe. New York City, NY: Oxford University Press 2017.
21. Dawson DL, MacMillan HL. Relationship management of the borderline patient: from understanding to treatment. New York City, NY: Routledge 1993.
22. Livesley J. Practical Management of Personality Disorder. New York City, NY: Guilford Press 2003.
23. Livesley J. Integrated Treatment for Borderline Personality Disorder: A Practical Guide to Combining Effective Treatment Methods. Cambridge, UK: Cambridge University Press 2017.
24. Gabbard GO. Psychodynamic psychiatry in clinical practice. 5th ed. Arlington, VA: American Psychiatric Association Publishing 2014.
25. Gabbard GO, Wilkinson SM. Management of countertransference with borderline patients. Northvale, New Jersey: Jason Aronson Inc. 2000.
26. Choi-Kain LW, Gunderson JG. Applications of good psychiatric management for borderline personality disorder: a practical guide. Washington, DC: American Psychiatric Association Publishing 2019.
27. Sonley AK, Choi-Kain LW. Good psychiatric management and dialectical behavior therapy: A clinician's guide to integration and stepped care. Washington, DC: American Psychiatric Association Publishing 2021.
28. Hong V, Casher M. The inpatient with borderline personality disorder. In: Casher M, Bess J, eds. Manual of Inpatient Psychiatry. 2nd ed. Cambridge, UK: Cambridge University Press 2020.
29. Hong V, Reddy P, Entenman S. Personality Disorders. In: Glick R, Zeller S, Berlin J, eds. Emergency Psychiatry: Principles and Practice. 2nd ed. Philadelphia, PA: Wolters Kluwer 2020.
30. Hong V, Kozada K, Shobassy A. Traitements dans les services d’urgence. In: Kolly S, Charbon P, Kramer U, eds. Trouble de la Personnalite Borderline: Pratiques Therapeutiques France: Elsevier Masson SAS 2019.
31. Curry A, Riddle M. Personality Disorders in the Emergency Department. In: Zun L, Nordstrom K, Wilson M, eds. Behavioral Emergencies for Healthcare Providers. 2nd ed. Switzerland: Springer Nature 2021.
32. Hong V, Pirnie L, Shobassy A. Antisocial and borderline personality disorders in the emergency department: conceptualizing and managing “Malingered” or “Exaggerated” symptoms. *Current Behavioral Neuroscience Reports* 2019;6(4):127-32.
33. Shaikh U, Qamar I, Jafry F, et al. Patients with borderline personality disorder in emergency departments. *Frontiers in psychiatry* 2017;8:136.
34. Broadbear JH, Rotella J-A, Lorenze D, et al. Emergency department utilisation by patients with a diagnosis of borderline personality disorder: An acute response to a chronic disorder. *Emerg Med Australas* 2022;34(5):731-37.
35. Cases C, Lafont Rapnouil S, Gallini A, et al. Evidence of practice gaps in emergency psychiatric care for borderline personality disorder: how can this be explained? *BMC Psychiatry* 2020;20(1):476. doi: 10.1186/s12888-020-02892-7
36. Acres K, Loughhead M, Procter N. From the community to the emergency department: A study of hospital emergency department nursing practices from the perspective of carers

For peer review only

For peer review only - http://bmjopen.bmj.com/site/about/guidelines.xhtml

of a loved one with Borderline Personality Disorder. *Health Soc Care Community* 2022;30(5):1789-97.
37. Paris J. Chronic Suicidality Among Patients With Borderline Personality Disorder. *Psychiatr Serv* 2002;53(6):738-42. doi: 10.1176/appi.ps.53.6.738
38. Paris J. Understanding Self-mutilation in Borderline Personality Disorder. *Harv Rev Psychiatry* 2005;13(3):179-85. doi: 10.1080/10673220591003614
39. Paris J. Half in Love with Easeful Death: The Meaning of Chronic Suicidality in Borderline Personality Disorder. *Harv Rev Psychiatry* 2004;12(1):42-48. doi: 10.1080/10673220490279161
40. Paris J. Is Hospitalization Useful for Suicidal Patients with Borderline Personality Disorder? *J Personal Disord* 2004;18(3):240-47. doi: 10.1521/pedi.18.3.240.35443
41. Paris J. Predicting and Preventing Suicide: Do We Know Enough to Do Either? *Harv Rev Psychiatry* 2006;14(5):233-40. doi: 10.1080/10673220600968662
42. Paris J. Can we predict or prevent suicide?: An update. *Prev Med* 2021;152(Pt 1):106353. doi: 10.1016/j.ypmed.2020.106353 [published Online First: 2021/09/21]
43. Goodman M, Roiff T, Oakes AH, et al. Suicidal Risk and Management in Borderline Personality Disorder. *Curr Psychiatry Rep* 2012;14(1):79-85. doi: 10.1007/s11920-011-0249-4

For peer review only

For peer review only - http://bmjopen.bmj.com/site/about/guidelines.xhtml

BMJ Open

Protocol for a Delphi consensus study to develop guidelines for the management of adults with borderline personality disorder in the emergency department

Journal:	*BMJ Open*
Manuscript ID	bmjopen-2023-075119.R2
Article Type:	Protocol
Date Submitted by the Author:	11-Oct-2023

Complete List of Authors:	Prosser, Aaron; McMaster University, Psychiatry<br>Hong, Victor; University of Michigan, Department of Psychiatry<br>Helfer, Bartosz; University of Wroclaw, Institute of Psychology; University of Wroclaw, Meta-Research Centre<br>Fudge, David; Hamilton, Department of Psychiatry and Behavioural Neurosciences<br>Patterson, Janet; Hamilton, Department of Psychiatry and Behavioural Neurosciences<br>Rosebush, Patricia; Hamilton, Department of Psychiatry and Behavioural Neurosciences<br>Frey, Benicio; McMaster University, McMaster Integrative Neuroscience Discovery and Study (MiNDS); St. Joseph's Healthcare Hamilton, Mood Disorders Program<br>Links, Paul; Hamilton, Department of Psychiatry and Behavioural Neurosciences

**Primary Subject Heading**:	Mental health
Secondary Subject Heading:	Emergency medicine
Keywords:	PSYCHIATRY, ACCIDENT & EMERGENCY MEDICINE, Personality disorders < PSYCHIATRY, Suicide & self-harm < PSYCHIATRY

SCHOLARONE™
Manuscripts

Protocol for a Delphi consensus study to develop guidelines for the management of adults with borderline personality disorder in the emergency department

Aaron Prosser MD MSc[1]
Victor Hong MD[2]
Bartosz Helfer PhD MSc[3,4]
David Fudge MD[1]
Janet Patterson MD[1]
Patricia Rosebush MD[1]
Benicio N. Frey MD PhD[1]
Paul Links MD MSc[1]

[1]Department of Psychiatry and Behavioural Neurosciences, McMaster University, Hamilton, Ontario, Canada
[2]Department of Psychiatry, University of Michigan, Ann Arbor, MI, United States
[3]Institute of Psychology, University of Wroclaw, Wroclaw, Poland
[4]Meta-Research Centre, University of Wroclaw, Wroclaw, Poland

Corresponding author: Aaron Prosser (aaron.prosser@medportal.ca)

Word count: 4,895

For peer review only

For peer review only - http://bmjopen.bmj.com/site/about/guidelines.xhtml

ABSTRACT

Introduction: Clinicians caring for adults with borderline personality disorder (BPD) in acute settings such as the emergency department (ED) have little evidence/guidance to base decisions on. Specific/detailed guidance for managing BPD in the ED is needed given the morbidity and mortality risks, high service utilization, unique challenges, and risk of iatrogenic interventions. The primary objective of this study is to use a consensus method to develop a guideline for managing adults with BPD in the ED. This protocol and the key questions for the guideline were developed with the advice of people with BPD and their family members/support persons.

Methods and analysis: We will perform a 4-phase Delphi study of an expert panel of clinicians, researchers, adults with BPD, and their family members/support persons. Various disciplines (psychiatry, psychology, emergency medicine, nursing, social work) and treatment approaches will be included in the expert panel. An online questionnaire will be developed from systematic reviews, qualitative assessments of pivotal literature, and opinions suggested by the panel (Phase 1). The panel will rate their agreement on opinions for each key question covering areas of emergency care of adults with BPD using two rounds of this questionnaire (Phases 2 and 3). Opinions meeting pre-defined thresholds for consensus will be brought to consensus meetings moderated by an independent chair (Phase 4). The purpose of these meetings is to finalise the set and phrasing of the opinions for each area of emergency care. These final opinions will be the recommendations in the guideline. If there are significant differences of opinion, the guideline will present both recommendations alongside one another.

Ethics and dissemination: This study has received ethics approval by the Hamilton Integrated Research Ethics Board in Hamilton, Ontario, Canada. The results of this study will be disseminated through peer-reviewed publications, conferences, and national professional and patient/family/support associations.

For peer review only - http://bmjopen.bmj.com/site/about/guidelines.xhtml

STRENGTHS AND LIMITATIONS OF THIS STUDY

- The Delphi method will be used due to the limited research on managing adults with BPD in the ED to inform a guideline.
- Our protocol incorporated the advice and feedback of people with BPD and their family members/support persons.
- Our methods use a questionnaire to minimise the disadvantages of group discussion (e.g., domination by individuals or pressure to conform) while the consensus meeting capitalises on its advantages (e.g., clarifying ambiguities or editing the phrasing of recommendations).
- The expert panel will include participants from different disciplines, backgrounds, and treatment approaches including adults with BPD, their family members and support persons, clinicians, and researchers.
- The expert panel might not be fully representative since recruitment is not based on random sampling and the recommendations will be based on consensus, which is based on shared knowledge and practical experience rather than scientific proof.

For peer review only

For peer review only - http://bmjopen.bmj.com/site/about/guidelines.xhtml

INTRODUCTION

The need for guidelines

Borderline personality disorder (BPD) is a highly heritable (~55%) condition whose core pathology is a disturbance in identity, attachment, and emotion regulation [1, 2]. BPD is often viewed as a trauma disorder since for many it emerges from psychosocial adversity interacting with a genetic diathesis [1, 2]. The symptomatic expression of BPD includes dysphoria, dysregulation, recurrent self-harm/suicide attempts, and conflicted/unstable relationships [3]. These symptoms are often severe and disabling, causing frequent crises and presentations to the emergency department (ED), with frequent suicidal thoughts and self-harm behaviors being behavioral trademarks of the disorder. Specialized outpatient treatments for BPD have been developed and shown to be effective in randomised controlled trials (RCTs) [4]. There is also limited RCT evidence for some medications targeting specific clusters of symptoms (e.g., emotion dysregulation, impulsivity) [5, 6]. These developments are a major accomplishment and have informed guidelines on BPD, notably the NICE [7], APA [8], and NHMRC [9] guidelines.

While crises and acute treatment are addressed in these guidelines, the sections are brief since most of the evidence pertains to outpatient settings. Therefore, we formed a Guideline Working Group (GWG) to oversee the development of a guideline on managing BPD in the ED. Our focus is on adults with BPD, although certainly many of the principles may apply to adolescents. Our primary aim will be to identify a set of recommendations for each key question (KQ) using Delphi consensus methods. Each KQ defines a key area of the emergency care of adults with BPD. Specific and detailed guidance for managing adults BPD in the ED is needed given the common pitfalls related to high service utilization, unique challenges in the emergency setting, risk of iatrogenic interventions, and recurrent suicide attempts and self-harm.

High service utilization and unique challenges

Adults with BPD are frequent visitors to the ED, representing upwards of 9% of all visits in some jurisdictions, with recurrent visits being common [10]. Reasons for presenting to the ED are numerous, including self-harm, suicide attempts, situational crises, adjustment reactions, depression, anxiety, emotion dysregulation, agitation, intoxication, withdrawal, substance induced psychosis, and housing/financial problems. There are patient, staff, and systems factors which create unique challenges to managing BPD in ED settings [11]. Some are listed in Table 1.

Table 1. Challenges to managing BPD in ED settings

Patient	Staff	System
• Recurrent ED visits and high service utilization.<br>• Disruptive behaviours.<br>• Chronic high risk for suicide and self-harm.<br>• Transference reactions.	• Problematic staff attitudes.<br>• Varying levels of training.<br>• Ill-defined/time-limited responsibility for patients.<br>• Countertransference reactions.	• Transient/changing ED staff.<br>• Changing ED workloads.<br>• Disposition problems.<br>• Availability of inpatient beds, aftercare services, and rapid outpatient follow-up.

High risk of iatrogenic interventions in the emergency department

For peer review only - http://bmjopen.bmj.com/site/about/guidelines.xhtml

Adults with BPD appear to be at higher risk of iatrogenic interventions in all clinical settings, particularly acute settings like the ED [11]. These include inadequate risk assessment, unnecessary inpatient hospitalisations, excessive use of medications, or unnecessary use of seclusion or physical/chemical restraints. Staff attitudes and countertransference reactions can also drive iatrogenesis. Staff who are otherwise empathic, validating, supportive, and effective clinicians may have negative or positive reactions which – if not examined, accepted, and contained – can be the driver of harmful interventions [12]. Negative reactions include withdrawal, rejection, hostility, helplessness, hopelessness, anxiety, or feeling overwhelmed. Positive yet often unhelpful reactions include rescue fantasies or feeling excessively protective of or responsible for a patient. Lack of awareness of the separation of acute and chronic risk factors may play a role in these reactions. Given the prevalence of comorbidities in individuals with BPD, this adds further complexity to the clinical decision making [13]. Iatrogenesis is likely caused by an interplay between these patient, staff, and systems factors, often amplified in the ED.

Risk of recurrent suicide attempts and self-harm

Recurrent suicidal acts, threats, urges and self-harm behaviours are defining symptoms of BPD.[3] The risk of suicide is 3%-10% in people with BPD and the average number of suicide attempts per individual is 3, with completed suicide occurring about once per 23 attempts [1]. About 75% of people with BPD self-harm and among these 90% repeatedly self-harm [1]. Taking on this risk is inherent to the work of caring for people with BPD. These risks are greatest for clinicians working in the ED because people with BPD presenting to the ED often do so because they are acutely dysphoric, actively suicidal, engaged in severe self-harm, and/or attempted to end their life. However, discerning the difference between acute vs. chronic suicide/self-harm risk is difficult when a patient is chronically at high risk. This is especially the case when the risk assessment/management must be done by a clinician who doesn't have a longitudinal relationship with the patient, which is the reality for most clinicians working in the ED. For these reasons, many clinicians experience fear not only for the safety of the people with BPD they care for, but also their own medico-legal liability. This is yet another reason why specific and detailed guidance for managing BPD in the ED is needed, especially regarding suicide/self-harm risk assessment/management.

METHODS AND ANALYSIS

Rationale for the Delphi method

Our project started by first determining the most appropriate method to develop the guideline. Guidelines are usually constructed from systematic reviews and meta-analyses of primary studies. However, our group was already aware that there is scant research in this area. This was confirmed by pilot systematic searches. Therefore, we decided to conduct a Delphi consensus study to come up with recommendations from clinicians, researchers, patients, and family members/support persons.

Development of key questions

For peer review only

Our KQs were built from the two main tasks which need to be addressed for all people with BPD who come to the ED. We used the analytic-graphical model as developed by Nelson et al. (2010) [14] to visualize the analytic framework and our KQs (Figure 1). A primary consideration in the ED is the risk assessment (KQ2) and, if a diagnosis isn't established yet, screening for BPD (KQ3). This usually starts with the emergency medicine team who may or may not ask the psychiatrist and/or mental health clinicians (e.g., psychiatric nurses, social workers) for further assistance in the assessment and disposition planning. Another consideration is to decide if to admit the patient to hospital (KQ4) or discharge the person to aftercare psychosocial services (KQ5). Alongside this process is the overall care of the person with BPD while they are in the ED (KQ1). Here we mean the *general interventions* to use with all patients throughout their stay in the ED (e.g., empathy, validation, management of countertransference and bias) and *specific interventions* (e.g., psychoeducation, medications) to address the specific needs of a patient. A related key area are interventions for the management of agitation and aggression in people with BPD in the ED (KQ6). Another key area is the involvement and role of family members and support persons (KQ7) and outpatient providers (KQ8) while people with BPD are in the ED. The last key areas are approaches to providing optimal medical care (KQ9) and effective environmental elements (e.g., physical layout, interior design) for people with BPD presenting to the ED in a mental health crisis (KQ10).

Based on this analytic framework, we constructed ten KQs:

1. What psychosocial and pharmacological interventions in the ED are associated with benefit while minimising harm for adults with BPD, with considerations regarding potential comorbidities?
    a. What interventions in the ED are effective?
    b. What interventions in the ED are harmful?
2. What are the effective approaches to assess and manage the risk of suicide and self-harm of adults with BPD, with considerations regarding potential comorbidities?
3. What should be included as part of the emergency evaluation of adults to screen for previously undiagnosed BPD?
4. What is the role of hospitalisation in managing adults with BPD?
    a. What are the indications for hospitalisation?
    b. What is the effectiveness of hospitalisation?
    c. What are the harms of hospitalisation?
5. What psychosocial aftercare services (including partial hospitalisation) can help adults with BPD who present to the ED but are not hospitalised?
6. What interventions in the ED for agitation and aggression are associated with benefit while minimising harm for adults with BPD?
    a. What interventions for agitation and aggression are effective?
    b. What interventions for agitation and aggression are harmful?
7. What are effective approaches to involving family members and support persons in the care of adults with BPD in the ED?
8. What are effective approaches to involving outpatient providers in the care of adults with BPD in the ED?
9. What are effective approaches to providing optimal medical care for adults with BPD presenting in a mental health crisis to the ED ?

For peer review only - http://bmjopen.bmj.com/site/about/guidelines.xhtml

10. What are the most effective environmental elements (physical layout and interior design) for adults with BPD in the ED?

We will systematically review the literature on each KQ, the results of which will be integrated into the Delphi process (see methods section for details). The Delphi process will be used to provide guidance on each of these KQs. It will draw on the expertise of people across disciplines (psychiatry, psychology, emergency medicine, nursing, social work), backgrounds (clinicians, researchers, patients, family, support persons), and treatment approaches. The latter will hopefully include Dialectical Behavior Therapy (DBT), Transference-Focused Psychotherapy (TFP), Mentalization-Based Treatment (MBT), Good Psychiatric Management (GPM), Schema Focused Therapy (SFT), Cognitive Behavior Therapy (CBT), Systems Training for Emotional Predictability and Problem Solving (STEPPS), Integrated Modular Treatment (IMT), and psychopharmacology. Other content experts who may not align with a specific treatment approach will be contacted for participation. While developing this protocol and the KQs included here, we sought advice from people with BPD and their family members/support persons on what are the most important questions to include from their perspective as well as feedback on an earlier draft of the protocol.

Patient and public involvement

While developing this protocol and the KQs included here, we sought advice from people with BPD and their family members/support persons on what are the most important questions to include from their perspective as well as feedback on an earlier draft of the protocol. VH contacted potential participants by utilizing the listserve from several advocacy organizations, and 13 provided constructive feedback including 3 persons with BPD and 9 family members and 1 support person of someone with BPD. Participants were asked to review the KQs, provide feedback, and propose other KQs. All 13 participants were those who themselves or had supported a person who had experience with an emergency department presentation for BPD-related issues. They agreed on the set of proposed KQs and 4 participants included specific suggestions. One person with BPD wanted KQ8 to be explored further. Family members and the support person requested additional KQs and edits, all of them were applied after consultation with the GWG and are listed here: (1) two additional KQ (9 and 10) were added, (2) the order of questions was changed (KQ4 became KQ1 and the numbering of the others was adjusted accordingly), (3) "support person" was added to KQ7 and the word “aggression” to KQ6. We have also edited the methods of the guideline development process below to include the wider support circle of people with BPD (going beyond family members or caregivers). 10 others expressed interest in participating in this phase of the project. 1 did not meet criteria because they did not have BPD, 1 ultimately did not choose to participate, and 8 did not respond to the protocol when it was shared. Additional feedback included preferences relating to the need to consider biases based on social-economic-status, race and gender as well during the guideline development process. Their preferences will be included in the Delphi process and shared with the guideline development team. Moreover, special attention will be given to these biases while preparing the systematic review. The guidelines will be shared with the participants in the development of guidelines.

Study design

For peer review only - http://bmjopen.bmj.com/site/about/guidelines.xhtml

This study will use the Delphi method in order to achieve consensus across the group of participants. This method is appropriate for our purposes because it maximises the benefits of group discussion while minimising its disadvantages [15]. The main disadvantages are domination by powerful individuals, coalitions, vested interests, the biasing effects of personality and seniority, that only one person can speak at a time, and group pressures for conformity. The Delphi method provides a structured, democratic, transparent, and iterative procedure with controlled feedback to participants to determine the extent of agreement amongst a group about a given issue [15, 16]. The procedure will consist of questionnaire development (Phase 1), rating evaluation (Phase 2), re-rating (Phase 3), and consensus meeting (Phase 4). Phase 1 will include systematic reviews of our KQs to incorporate any recommendations which may come from the existing literature.

Study governance

The GWG is comprised of six psychiatrists (VH, DF, JP, PR, BF, PL), a psychiatry resident (AP), and a meta-researcher (BH) with expertise in guideline development. All clinicians in the GWG currently treat people with BPD in outpatient, inpatient, and emergency settings. One of the members (PL) was a co-developer of GPM [1, 17] and another (VH) published an expert opinion on the management of BPD in the ED based on GPM principles [11]. As a result, PL and VH will also be participants given their recognized expertise. However, this also is a potential source of bias. We detail how we will mitigate this (Appendix A). Study methods for the protocol were established in virtual meetings and email correspondence. The GWG will meet at minimum once quarterly and at higher frequency as needed until projected completion. Meetings will be held virtually and we will have regular email correspondence. To further address any outstanding issues with study governance, we will follow the complete interaction approach with full transparency between the systematic review team and eventual guideline developers [18].

Involvement of persons with BPD and their families/support persons

Adults with BPD, their family members and support persons will have a voice in these guidelines since the recommendations will directly impact them. We recognize that there may be differences of opinion between patients/family/support persons and clinicians/researchers on some questions during the Delphi process. If there are significant differences in opinion, we will be transparent about these differences in the guideline. This ensures that patient/family/support persons voices are not silenced by majority opinion since we anticipate there will be more clinician/researcher participants. Patients or patients' representatives will be continuously involved throughout the guideline development process following a modified model by Armstrong et al. (2010) [19]. Following NICE recommendations, at least at least two patient/caregiver/advocate members will be involved in the Delphi process and consensus meeting as panelists [20].

Participant selection/recruitment

For peer review only - http://bmjopen.bmj.com/site/about/guidelines.xhtml

Participants will be included if they are clinicians and/or researchers who have significant experience in the management of BPD in the ED. We will also recruit patients and family members/support persons. The clinician/researcher sample will be recruited using a number of strategies. First, we will contact national professional organizations for emergency medicine, psychiatry, nursing, and social work in English-speaking countries, specifically the United States, Canada, United Kingdom, Ireland, South Africa, Australia, and New Zealand. We will ask them to advertise our study through their email mailing lists. Second, the GWG is already aware of a number of experts who we will invite to participate. These individuals were either a developer or published expert in a treatment approach for BPD, a clinician/group member on the NICE, APA, and NHMRC guidelines, or is a published expert in the management of BPD in the ED. Third, the publication of this protocol will also help advertise this study. Patients and family members/support persons will be recruited by contacting national patient advocacy and support organizations. We will ask these organizations to advertise our study through their email mailing lists and social media to help with recruitment. We will ask all participants to provide suggestions too. Readers of this protocol may also give suggestions. Suggestions can be made to the GWG by contacting the corresponding author of this protocol (aaron.prosser@medportal.ca). The deadline for suggesting a participant is 3 months after the publication date of this protocol.

Representation

We believe that representation is important to the guideline development process. Thus we will attempt to build a balanced panel of experts based on the following demographic data: discipline (psychiatry, psychology, nursing, emergency medicine, social work), group (clinician, researcher, adult with BPD, family member/support person), treatment modality allegiance, ethnicity, gender, and sexual orientation. In order to do this, at the recruitment stage, the study coordinator will email a form to potential participants who have expressed interest to participate. In this form, participants will be invited to provide basic demographic data to allow us to build a balanced panel. There will be an option to select “Prefer not to answer”, “Do not know”, and “Other” for all questions. If a demographic factor has reached saturation, the person may be ineligible to participate. This demographic data will be collected before consent is obtained to participate in this study. Therefore, if a person is ineligible to participate or doesn’t consent to participate in the study after reviewing the consent form, the demographic form will be promptly deleted and will not be kept part of the study records. If the person is eligible to participate and consents, the form will be retained as part of the study records, de-identified, and statistically summarized so that we can show the representativeness of the expert panel in the publication of the guideline. Saturation will be determined on a case-by-case basis depending on our pool of potential participants and the representativeness of those who have already consented to participate.

Delphi procedure

The Delphi process is an iterative procedure which typically proceeds in three phases [16]. We will add a fourth phase in order to have a consensus meeting moderated by an independent chair, thus combining the Delphi method with the consensus conference method. This is because we believe that an open-ended group discussion has advantages over questionnaires. In particular, group discussion can be helpful for clarifying ambiguities and

For peer review only - http://bmjopen.bmj.com/site/about/guidelines.xhtml

differences and editing phrasing for the final recommendations of the guideline. Furthermore, a meeting provides an opportunity to reach consensus on issues where consensus has not yet been achieved. Questionnaires will be delivered and collected from participants using REDCap. Data will be de-identified by giving participants an arbitrary study ID linked to their questionnaire responses, however no identifying data will be collected (e.g., names, age, date of birth) with the questionnaire responses.

Phase 1 – Questionnaire development

Phase 1 has three goals: (1) create the expert panel, (2) create a pool of opinions on each KQ from three sources of information, and (3) create an online questionnaire using this pool of opinions for rating evaluation by the expert panel. The expert panel will be created using the selection and recruitment strategies described above.

The first source of information we will use for generating the opinion pool will be systematic reviews of the literature on each KQ. Full details of our methods, including search syntax, are in Appendix B of the supplementary materials. Summary opinions will be generated by the GWG based on the results of these systematic reviews.

The second source of information is a qualitative assessment of pivotal guidelines, psychotherapy manuals, book chapters, and articles (Appendix C of the supplementary materials). This allows us to include pivotal literature which will not necessarily be identified in the systematic reviews (e.g., psychotherapy manuals). AP will search these sources to extract a narrative summary of the opinions on each KQ. Quality control will be confirmed by an independent assessor on a random 20% subset of the opinions from these pivotal sources.

The third source of information will be our expert panel. We will invite the expert panelists to provide written opinions on each KQ once the participant has consented to participate. They will be given the results of the systematic reviews and qualitative assessment in tabular form to review before providing their opinions.

Any evidence synthesis components employed in the process of questionnaire development will follow the relevant recommendations of the Institute of Medicine's Committee on Standards for Systematic Reviews of Comparative Effectiveness Research, especially regarding the qualitative assessment [21].

We anticipate there will be nearly identical opinions for some KQs across the three sources of information. This will lead to significant redundancy which will unnecessarily burden the participants with repeating ratings on nearly identical opinions. For this reason, two independent assessors will thematically group the identical opinions and meet for consensus. After consensus on the groupings is achieved, they will then draft a single summary opinion. This pool of non-redundant opinions will be grouped together under their respective KQs and prepared for circulation to all participants in the form of an online questionnaire hosted on REDCap. The sources of the opinions will be masked to all participants to eliminate bias (e.g., allegiance to a treatment approach).

Phase 2 – Rating evaluation

Participants will be emailed the questionnaire and asked to rate each opinion individually on a 5-point Likert scale of agreement: 1 = strongly disagree, 2 = disagree, 3 = neutral, 4 = agree, and 5 = strongly agree. The questionnaire responses will be de-identified and the opinions will be

For peer review only - http://bmjopen.bmj.com/site/about/guidelines.xhtml

grouped into their respective KQs. Once all the questionnaires have been received, the ratings will be statistically summarized for each opinion. This will involve calculating and graphically depicting the median, inter-quartile range (IQR), and the percentages in each response category for each opinion, separately for patient/family/support persons and clinician/researcher groups. This statistical summary will be given as feedback to all participants, along with their own rating, so they can see where their ratings are relative to others for each opinion under each KQ.

Phase 3 – Re-rating

Participants will be emailed the same questionnaire and asked to re-rate their agreement on each opinion. This gives participants the opportunity to change their ratings in light of the feedback. After all the questionnaires have been received, the ratings will be statistically analysed using pre-defined consensus thresholds (Table 2).

Opinions meeting threshold for essential, recommended, or no consensus will be brought forward to the consensus meeting for review. If at least one opinion meets one of these thresholds, then we will organize a virtual video meeting. Opinions meeting threshold for exclusion will not be brought to the consensus meeting. If no opinions can be brought to the consensus meeting, then a third round of ratings will be performed. If no opinions can be brought forward after a third round, then the consensus meeting will be held on all the opinions.

Table 2. Consensus thresholds

Essential	The first quartile (Q1) of the ratings for the opinion is > 3 (neutral) in *both* the patient/family/support persons *and* clinician/researcher groups.
Recommended	The first quartile (Q1) of the ratings for the opinion is > 3 (neutral) in *either* the patient/family/support persons *or* clinician/researcher groups.
Exclusion	The third quartile (Q3) of the ratings for the opinion is < 3 (neutral) in *both* the patient/family/support persons *and* clinician/researcher groups.
No consensus	Essential, recommended, or exclusion thresholds have not been met.

Phase 4 – Moderated consensus meeting

The purpose of this meeting is to finalise the set of opinions and phrasing for each KQ. We will have an independent chair for this meeting to minimise the disadvantages of group discussion by moderating the discussion. The chair will also help identify when significant differences of opinion between patient/family/support persons vs. clinicians/researchers exist. Multiple meetings may take place at the discretion of the chair. The meetings will be recorded for reference if necessary and securely stored. However, all opinions will be considered final at the end of this consensus meeting process. Email correspondence will be allowed during Phase 4 amongst the chair and participants in order to edit phrasing of opinions.

Guideline development

The final opinions from Phase 4 will be the recommendations in the guideline, which will be written up by the GWG. No changes to the opinions will be made by the GWG. If there are significant differences of opinion, the guideline will present the differing recommendations alongside one another.

For peer review only - http://bmjopen.bmj.com/site/about/guidelines.xhtml

Conflict of interest management

All members of the GWG and potential participants will submit a conflict of interest (COI) disclosure form, developed based on the International Committee of Medical Journal Editors (ICMJE) form. Potential participants will have to submit this form along with the demographic form in order to be considered for eligibility to participate in the expert panel. COIs will be considered by the study coordinator and, if needed, by the GWG, to determine eligibility. The principle guiding eligibility decisions is to ensure that COIs represent no more than a minority of all study participants. The chair of the consensus meeting will have no COI. Any reported COI will be discussed by the GWG and documented in the guideline in light of its potential impact on the proposed recommendations. This COI data will be collected before consent is obtained to participate in this study. Therefore, if a person is ineligible to participate or doesn't consent to participate in the study after reviewing the consent form, the COI form will be promptly deleted and will not be kept part of the study records.

Statistical analysis

We will analyse the ratings using medians, IQRs, and percentages for each response category. These values will also be graphically depicted in the feedback provided to the participants. We will statistically compare the agreement ratings between patient/family/support persons vs. clinician/researcher groups using independent samples *t*-tests or Wilcoxon rank-sum tests depending on if parametric assumptions are met or not, respectively, and document these differences in the final version of the guideline. All tests of significance will be two-tailed with α set at 0.05.

ETHICS

This study has received ethics approval by the Hamilton Integrated Research Ethics Board in Hamilton, Ontario, Canada.

DISSEMINATION PLAN

We anticipate that the guideline emerging from this Delphi study will be published in a high-impact peer-reviewed journal. The guideline will be disseminated to regional, national, and international audiences through presentations at regional, national, and international conferences. Finally, we will disseminate the guideline through the national and international associations for psychiatry, psychology, emergency medicine, nursing, and social work.

DISCUSSION

Clinicians caring for adults with BPD in acute settings such as the ED have little guidance or evidence to base their decisions on. In this vacuum, patients are at risk of a wide variety of treatment approaches and practices, ranging from excellent to iatrogenic. Therefore, specific and detailed guidance for managing adults with BPD in the ED is urgently needed for the sake of patients and their families/support persons. Consensus methods such as Delphi play

For peer review only - http://bmjopen.bmj.com/site/about/guidelines.xhtml

For peer review only

an important role in science where there is limited or contradictory evidence [16]. Through a structured process, these methods assess the degree of agreement and/or resolve disagreement amongst experts. The consensus can provide helpful scaffolding for clinical guidelines until empirical study fills the gaps. Furthermore, the clinical guidelines will provide important hypotheses for these empirical investigations. This is the rationale for our Delphi study. The opinions emerging from this study will become the first consensus guideline on the management of adults with BPD in the ED.

The nominal group technique is another consensus method used in health research.[15] It uses a facilitated meeting of experts who gather information, rate, discuss, and re-rate/discuss a series of items, questions, or opinions. Each participant contributes their ideas to the facilitator who thematically groups the ideas for discussion. The ideas are then rated, summarized, and the summary is fed back to the participants for discussion and re-rating. The final ratings are then summarized and evaluated for consensus using pre-defined thresholds. The disadvantage of this technique is that it is often not feasible for all relevant experts to participate in the meeting. This is why typically the nominal group technique usually involves small groups of 9-12 individuals [16]. Moreover, experts often do not have the time to devote to these steps commensurate with the complexity of the issues. By contrast, the Delphi method allows researchers to synthesize a large amount of information across many individuals with relatively minimal imposition on the participants' time.

The main limitation of this study is that, even if we achieve consensus on some or all opinions, this does not guarantee that the correct recommendations have been found. Consensus does not necessarily imply truth. Only empirical studies can validate the opinions generated from a consensus process, which at this time are lacking. Therefore, our study is best viewed as a structured sampling of expert opinions rather than an attempt to reach a definitive conclusion about what are truly the best practices in managing adults with BPD in the ED. Another limitation is that our expert panel will not be representative of the disciplines, backgrounds, and treatment approaches we are sampling. Relatedly, we anticipate there will be more clinician/researcher participants on the panel, which risks silencing patient/family/support persons voices. This risk will be mitigated by documenting and discussing the recommendations of patient/family/support persons if significant differences of opinion exist. Additionally, this study does not focus on guidelines for pediatric patients with BPD. While many of the guidelines here will be relevant for pediatric patients, a logical next step would be to develop guidelines for that population, given the inherent differences in the assessment and management of adults versus youth. Notwithstanding these limitations, we hope the guidance emerging from this Delphi study will be a helpful clinical resource to improve the care of adults with BPD in the ED.

For peer review only

For peer review only - http://bmjopen.bmj.com/site/about/guidelines.xhtml

Contributors

AP, VH, BH, DF, JP, PR, BF, and PL are responsible for the design of the study and early drafts of the protocol. PL is the principal investigator. AP is the study coordinator and wrote the first draft of the protocol. AP, VH, BH, and PL are responsible for final draft of the protocol manuscript. All authors have read and approved the final manuscript, provided final approval for publication, and agree to be accountable for all aspects of this work. AP attests all listed authors meet authorship criteria and that no others meeting criteria have been omitted.

Funding

This research was funded in part by National Science Centre, Poland (grant number 2021/41/B/HS6/02844). For the purpose of Open Access, the author has applied a CC-BY public copyright licence to any Author Accepted Manuscript (AAM) version arising from this submission.

Competing interests

PL is a co-developer of GPM and VH published an expert opinion on the management of BPD in the ED based on GPM principles.

Acknowledgement

We would like to express our gratitude to the people with BPD and their family members/support persons for reviewing and commenting on an earlier draft of this protocol, as well as providing advice on the key questions for the guideline. We would also like to thank the patients with BPD who we have cared for and learned from over the years. Their lives inspired this project.

For peer review only

Figure legends

Figure 1. Analytic framework and key questions (KQs).

For peer review only

For peer review only - http://bmjopen.bmj.com/site/about/guidelines.xhtml

REFERENCES

1. Gunderson, J. and P. Links, *Handbook of Good Psychiatric Management for Borderline Personality Disorder*. 2014, Washington, DC: American Psychiatric Association Publishing.
2. Livesley, J., *Integrated Treatment for Borderline Personality Disorder: A Practical Guide to Combining Effective Treatment Methods*. 2017, Cambridge, UK: Cambridge University Press.
3. APA, *Diagnostic and Statistical Manual of Mental Disorders*. 5th ed. 2013, Washington, DC: Author.
4. Cristea, I.A., et al., *Efficacy of Psychotherapies for Borderline Personality Disorder: A Systematic Review and Meta-analysis.* JAMA Psychiatry, 2017. **74**(4): p. 319-328.
5. Lieb, K., et al., *Pharmacotherapy for borderline personality disorder: Cochrane systematic review of randomised trials.* The British Journal of Psychiatry, 2010. **196**(1): p. 4-12.
6. Stoffers-Winterling, J., O.J. Storebø, and K. Lieb, *Pharmacotherapy for borderline personality disorder: an update of published, unpublished and ongoing studies.* Current psychiatry reports, 2020. **22**: p. 1-10.
7. Health, N.C.C.f.M., *Borderline Personality Disorder: The NICE Guideline on Treatment and Management. National Clinical Practice Guideline No. 78*. 2009, The British Psychological Society and The Royal College of Psychiatrists.
8. Association, A.P., *Practice guideline for the treatment of patients with borderline personality disorder*. 2001: American Psychiatric Association Practice Guidelines.
9. Smith, M., et al., *Clinical practice guideline for the management of borderline personality disorder.* 2012, National Health and Medical Research Council (NHMRC): Melbourne.
10. Pascual, J.C., et al., *Hospitalization and Pharmacotherapy for Borderline Personality Disorder in a Psychiatric Emergency Service.* Psychiatric Services, 2007. **58**(9): p. 1199-1204.
11. Hong, V., *Borderline personality disorder in the emergency department: good psychiatric management.* Harvard Review of Psychiatry, 2016. **24**(5): p. 357-366.
12. Gabbard, G.O. and S.M. Wilkinson, *Management of countertransference with borderline patients*. 2000, Northvale, New Jersey: Jason Aronson Inc.
13. Shah, R. and M.C. Zanarini, *Comorbidity of Borderline Personality Disorder: Current Status and Future Directions.* Psychiatric Clinics of North America, 2018. **41**(4): p. 583-593.
14. Nelson, H.D., et al., *Screening for Osteoporosis: An Update for the U.S. Preventive Services Task Force.* Annals of Internal Medicine, 2010. **153**(2): p. 99-111.
15. Powell, C., *The Delphi technique: myths and realities.* Journal of Advanced Nursing, 2003. **41**(4): p. 376-382.
16. Jones, J. and D. Hunter, *Qualitative Research: Consensus methods for medical and health services research.* BMJ, 1995. **311**(7001): p. 376-380.
17. Gunderson, J. and P. Links, *Borderline Personality Disorder: A Clinical Guide*. 2008, Washington, DC: American Psychiatric Association Publishing.
18. Lau, J., *Models of interaction between clinical practice guidelines (CPG) groups and systematic review (SR) teams*, in *IOM Committee on Standards for Developing*

For peer review only - http://bmjopen.bmj.com/site/about/guidelines.xhtml

Trustworthy Clinical Practice Guidelines meeting (January 12, 2010). 2010: Washington, DC.
19. Armstrong, M.J., et al., *Framework for enhancing clinical practice guidelines through continuous patient engagement.* Health Expectations, 2017. **20**(1): p. 3-10.
20. Excellence, N.I.f.H.a.C., *The Guidelines Manual*. 2012, National Institute for Health and Clinical Excellence (NICE).
21. Eden, J., et al., *Finding What Works in Health Care: Standards for Systematic Reviews*. 2011, Washington, DC: Institute of Medicine (US) Committee on Standards for Systematic Reviews of Comparative Effectiveness Research, National Academies Press.

For peer review only

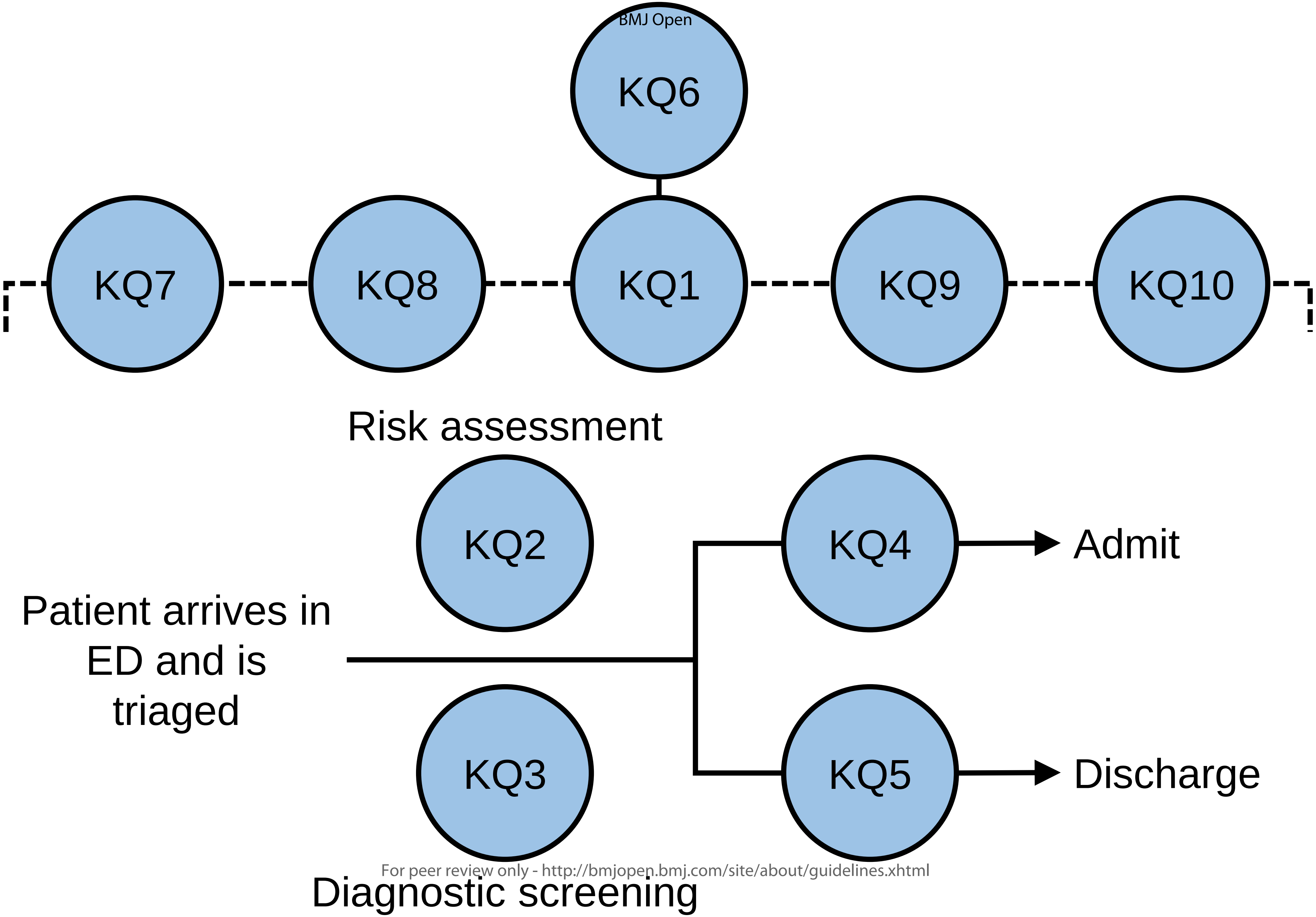
KQ6
KQ7
KQ8
KQ1
KQ9
KQ10
Risk assessment
KQ2
KQ4
Admit
Patient arrives in ED and is triaged
KQ3
KQ5
Discharge
Diagnostic screening

Supplementary Materials

Protocol for a Delphi consensus study to develop guidelines for the management of adults with borderline personality disorder in the emergency department

For peer review only

For peer review only - http://bmjopen.bmj.com/site/about/guidelines.xhtml

APPENDIX A

Risk of bias mitigation

PL and VH will be participants in the Delphi process given their recognized expertise. This means there is a potential source of bias due to their involvement in developing GPM[1 2] and GPM applications to ED settings.[3] This will be mitigated by ensuring they have no access to the data or involvement in the data analysis. The chair of the consensus meeting will have no affiliation with a treatment approach.

For peer review only

For peer review only - http://bmjopen.bmj.com/site/about/guidelines.xhtml

APPENDIX B

Protocol for systematic reviews

1. Review questions

We will conduct ten systematic reviews to search the literature for articles on the following key questions (KQs) related to the key areas of care of adults with borderline personality disorder (BPD) in the emergency department (ED):

1. What psychosocial and pharmacological interventions in the ED are associated with benefit while minimising harm for adults with BPD, with considerations regarding potential comorbidities?
    a. What interventions in the ED are effective?
    b. What interventions in the ED are harmful?
2. What are the effective approaches to assess and manage the risk of suicide and self-harm of adults with BPD, with considerations regarding potential comorbidities?
3. What should be included as part of the emergency evaluation of adults to screen for previously undiagnosed BPD?
4. What is the role of hospitalisation in managing adults with BPD?
    a. What are the indications for hospitalisation?
    b. What is the effectiveness of hospitalisation?
    c. What are the harms of hospitalisation?
5. What psychosocial aftercare services (including partial hospitalisation) can help adults with BPD who present to the ED but are not hospitalised?
6. What interventions in the ED for agitation and aggression are associated with benefit while minimising harm for adults with BPD?
    a. What interventions for agitation and aggression are effective?
    b. What interventions for agitation and aggression are harmful?
7. What are effective approaches to involving family members and support persons in the care of adults with BPD in the ED?
8. What are effective approaches to involving outpatient providers in the care of adults with BPD in the ED?
9. What are effective approaches to providing optimal medical care for adults with BPD presenting in a mental health crisis to the ED ?
10. What are the most effective environmental elements (physical layout and interior design) for adults with BPD in the ED?

2. Searches

The following databases will be searched from inception: MEDLINE, MEDLINE In-Process, Embase, Global Health, and PsychInfo with no language restrictions. The results of the individual databases will be transferred into a reference manager program and duplicates will be eliminated. To search for additional reports, we will search the reference lists of the included studies. A PRISMA flow chart will be included in the final publication.

For peer review only - http://bmjopen.bmj.com/site/about/guidelines.xhtml

3. Search strategy

KQ1

1. ((borderline$ or cluster$) adj3 personalit$).mp
2. (borderline$ or BPD).mp
3. ((difficult$ or challeng$) adj3 patient$).mp
4. (recurrent$ or repeat$) adj3 (suicid$ or self-harm$ or self-injur$ or self-cut$ or mutilat$).mp
5. or/1-4
6. (emergency adj3 (department$ or room$ or treatment$ or medicine$ or health service$)).mp
7. 5 and 6

KQ2

1. ((borderline$ or cluster$) adj3 personalit$).mp
2. (borderline$ or BPD).mp
3. ((difficult$ or challeng$) adj3 patient$).mp
4. (recurrent$ or repeat$) adj3 (suicid$ or self-harm$ or self-injur$ or self-cut$ or mutilat$).mp
5. or/1-4
6. ((suicid$ or self harm$ or self injur$ or self cut$ or mutilat$ or NSSI or DSH) and (risk$ adj5 (assess$ or evaluat$))).mp
7. 5 and 6

KQ3

1. ((borderline$ or cluster$) adj3 personalit$).mp
2. (borderline$ or BPD).mp
3. ((difficult$ or challeng$) adj3 patient$).mp
4. (recurrent$ or repeat$) adj3 (suicid$ or self-harm$ or self-injur$ or self-cut$ or mutilat$).mp
5. or/1-4
6. (assessment$ or screening$ or diagnostic$).mp
7. 5 and 6

KQ4

1. ((borderline$ or cluster$) adj3 personalit$).mp
2. (borderline$ or BPD).mp
3. ((difficult$ or challeng$) adj3 patient$).mp
4. (recurrent$ or repeat$) adj3 (suicid$ or self-harm$ or self-injur$ or self-cut$ or mutilat$).mp
5. or/1-4
6. (hospitalization$ or hospitalisation$ or admit$ or admission$).mp
7. (((single$ or double$ or trebl$ or tripl$) adj5 (blind$ or mask$)) or trial$ or (clinic$ adj2 trial$) or open-label$ or openlabel$ or (pre$ adj5 post$) or (pretest adj5 posttest) or cohort or (program$ adj5 evaluate$) or ((assurance$ or improv$ or indicator$ or measure$ or metric$) adj2 quality)).mp

For peer review only - http://bmjopen.bmj.com/site/about/guidelines.xhtml
For peer review only

8. 5 and 6 and 7

KQ5

1. ((borderline$ or cluster$) adj3 personalit$).mp
2. (borderline$ or BPD).mp
3. ((difficult$ or challeng$) adj3 patient$).mp
4. (recurrent$ or repeat$) adj3 (suicid$ or self-harm$ or self-injur$ or self-cut$ or mutilat$).mp
5. or/1-4
6. (emergency adj3 (department$ or room$ or treatment$ or medicine$ or health service$)).mp
7. (aftercare$ or (discharge adj5 (plan$ or service$ or manag$ or treatment$ or therap$))).mp
8. 5 and 6 and 7

KQ6

1. ((borderline$ or cluster$) adj3 personalit$).mp
2. (borderline$ or BPD).mp
3. ((difficult$ or challeng$) adj3 patient$).mp
4. (recurrent$ or repeat$) adj3 (suicid$ or self-harm$ or self-injur$ or self-cut$ or mutilat$).mp
5. or/1-4
6. (agitat$ or aggress$ or violen$ or anxiet$ or anxious$).mp
7. (((single$ or double$ or trebl$ or tripl$) adj5 (blind$ or mask$)) or trial$ or (clinic$ adj2 trial$) or open-label$ or openlabel$ or (pre$ adj5 post$) or (pretest adj5 posttest) or cohort or (program$ adj5 evaluate$) or ((assurance$ or improv$ or indicator$ or measure$ or metric$) adj2 quality)).mp
8. (emergency adj3 (department$ or room$ or treatment$ or medicine$ or health service$)).mp
9. 5 and 6 and 9 and 8

KQ7

1. ((borderline$ or cluster$) adj3 personalit$).mp
2. (borderline$ or BPD).mp
3. ((difficult$ or challeng$) adj3 patient$).mp
4. (recurrent$ or repeat$) adj3 (suicid$ or self-harm$ or self-injur$ or self-cut$ or mutilat$).mp
5. or/1-4
6. (emergency adj3 (department$ or room$ or treatment$ or medicine$ or health service$)).mp
7. (famil$ or partner$ or caregiver$ or parent$ or guardian$ or children$ or support$).mp
8. 5 and 6 and 7

KQ8

1. ((borderline$ or cluster$) adj3 personalit$).mp
2. (borderline$ or BPD).mp

For peer review only
For peer review only - http://bmjopen.bmj.com/site/about/guidelines.xhtml

3. ((difficult$ or challeng$) adj3 patient$).mp
4. (recurrent$ or repeat$) adj3 (suicid$ or self-harm$ or self-injur$ or self-cut$ or mutilat$).mp
5. or/1-4
6. (emergency adj3 (department$ or room$ or treatment$ or medicine$ or health service$)).mp
7. ((outpatient$ or ambulator$) adj5 (provider$ or team$ or caregiver$ or treater$ or physician$ or psychiatrist$ or psychologist$)).mp
8. 5 and 6 and 7

KQ9

1. ((borderline$ or cluster$) adj3 personalit$).mp
2. (borderline$ or BPD).mp
3. ((difficult$ or challeng$) adj3 patient$).mp
4. (recurrent$ or repeat$) adj3 (suicid$ or self-harm$ or self-injur$ or self-cut$ or mutilat$).mp
5. or/1-4
6. (emergency adj3 (department$ or room$ or treatment$ or medicine$ or health service$)).mp
7. ((medical$ or physical health$) adj3 (care$ or treatment$ or management$)).mp
8. 5 and 6 and 7

KQ10

1. ((borderline$ or cluster$) adj3 personalit$).mp
2. (borderline$ or BPD).mp
3. ((difficult$ or challeng$) adj3 patient$).mp
4. (recurrent$ or repeat$) adj3 (suicid$ or self-harm$ or self-injur$ or self-cut$ or mutilat$).mp
5. or/1-4
6. (emergency adj3 (department$ or room$ or treatment$ or medicine$ or health service$)).mp
7. (environment$ or architecture$ or design$ or layout$ or decor or ergonomic$).mp
8. 5 and 6 and 7

4. Condition or domain being studied

We will study the key areas of emergency care of adults with BPD defined by our KQs.

5. Article inclusion/exclusion criteria

Inclusion: the report is (1) a primary study (clinical trial, cohort study, cross-sectional study, pre/post evaluation, program evaluation, or quality assurance), guideline, standards, consensus statement, decision-making tool, assessment/diagnostic/screening tool, or expert opinion, (2) includes a sample of at least >75% adults with BPD in an emergency setting (if a primary study) or is about adults with BPD in emergency settings (if a guideline, standard, consensus statement, decision-making tool, assessment/diagnostic/screening tool, or expert opinion), and is about

For peer review only - http://bmjopen.bmj.com/site/about/guidelines.xhtml

either (i) the overall care of adults with BPD in the ED using psychosocial and/or pharmacological interventions (KQ1), (ii) suicide/self-harm risk assessment and/or management of adults with BPD (KQ2), (iii) diagnostic screening for BPD, (iv) hospitalisation of adults with BPD (KQ4), (v) psychosocial aftercare services (including partial hospitalisation) of adults with BPD who present to the ED but are not hospitalised (KQ5), (vi) the pharmacological and/or non-pharmacological management of agitation/aggression in adults with BPD (KQ6), (vii) approaches to involving family members/support persons in the care of adults with BPD in the ED (KQ7), (viii) approaches to involving outpatient providers in the care of adults with BPD in the ED (KQ8), (ix) general medical care of adults with BPD presenting to the ED in a mental health crisis (KQ9), and (x) environmental elements (physical layout and interior design) of the ED in relation to adults with BPD presenting to the ED in a mental health crisis (KQ10).
Exclusion: none.

6. Main outcome(s)

The primary outcome for the primary studies is efficacy which will be categorized as follows. These outcomes will be summarized narratively along with an effect size if it can be calculated from the report. If an effect size cannot be calculated from the report, the outcome will be narratively summarized only.

1. *Suicidality outcomes*
   i. Self-harm
   ii. Suicidal ideation
   iii. Suicide attempt
   iv. Death by suicide
2. *BPD-related outcomes*
   i. Emotion dysregulation
   ii. Depression
   iii. Anxiety
   iv. Anger/irritability
   v. Aggression
   vi. Paranoia
   vii. Dissociation
   viii. Interpersonal dysfunction
   ix. Self dysfunction (e.g., identity disturbance)
3. *Global outcomes*
   i. Quality of life
   ii. Global psychosocial functioning
   iii. Global symptom severity
4. *Service utilization outcomes*
   i. Patient satisfaction
   ii. Hospitalisation
   iii. ED visits
   iv. Adherence with medications
   v. Adherence with appointments.
   vi. Time to all-cause treatment discontinuation

For peer review only - http://bmjopen.bmj.com/site/about/guidelines.xhtml

We will exclude measures when they cannot be categorized. Note, suicidal ideation, planning, and behaviour (including self-harm) will be always coded under behavioural dyscontrol, even if in the report it may have been coded as a tolerability outcome. The primary outcome for non-primary studies (i.e., guideline, standards, consensus statement, decision-making tool, assessment/diagnostic/screening tool, or expert opinions) will be a narrative summary of the opinion on a given KQ.

Measures of effect: See Additional outcomes section below.

7. Additional outcome(s)

The secondary outcome for the primary studies is tolerability which will be categorized as follows. These outcomes will be summarized narratively along with an effect size if it can be calculated from the report. If an effect size cannot be calculated from the report, the outcome will be narratively summarized only. There will be no secondary outcomes for non-primary studies.

- Any adverse event
- Serious adverse event
- Death (non-suicide)
- Nausea
- Constipation
- Diarrhea
- Dry mouth
- Headache
- Dizziness
- Sedation
- Sleep disturbance
- Restlessness/agitation
- Menstrual changes
- Increased appetite
- Weight gain
- Decreased appetite
- Weight loss
- Extrapyramidal symptoms
- Akathisia
- Tardive dyskinesia
- Tremor (any type)
- Any other neurological dysfunction
- Cognitive dysfunction
- Sexual dysfunction
- Systolic blood pressure
- Diastolic blood pressure
- QTc interval
- Liver enzymes (AST, ALT, ALP, or GGT)
- Bilirubin (total, direct, or indirect)
- Albumin
- International normalized ratio (INR)
- Low-density lipoprotein (LDL)
- High-density lipoprotein (HDL)
- Triglycerides
- HbA1C
- Leukocyte count
- Hemoglobin
- Platelet count
- Creatinine
- Urea or blood urea nitrogen (BUN)
- Estimated glomerular filtration rate
- Sodium
- Potassium
- Calcium
- Phosphate
- Magnesium
- Creatine kinase
- Prolactin
- Thyroid stimulating hormone
- Thyroid hormone

For peer review only

For peer review only - http://bmjopen.bmj.com/site/about/guidelines.xhtml

- Weight loss
- Endpoint weight
- Total cholesterol
- Fasting blood glucose

Measures of effect:

For each primary study we will calculate the standardized mean difference (SMD, Hedges' *g*) for continuous measures (efficacy and tolerability data). If the study includes a comparator, we will use endpoint data between the active intervention vs. control intervention to calculate the SMD. If the study does not include a comparator, we will use pre vs. post data to calculate the SMD. If only change scores are reported, we will use change scores to calculate the SMD between the active intervention vs. control intervention. If both are reported, endpoint scores will be used. Dichotomous outcomes measured throughout the study and reported at as totals at endpoint (e.g., total number of self-harm episodes during the trial) will be considered endpoint data. Intention-to-treat (ITT) data will be used whenever available. Negative SMDs will indicate the active intervention was more beneficial than control intervention. The relative risk (RR) will be calculated for dichotomous tolerability data only. RR less than 1 will indicate the active intervention had less adverse effects than control intervention. Effect sizes of dichotomous efficacy data will be converted to the SMD using the procedure discussed shortly.

Combining multiple effect sizes within trials:

If a study reports multiple outcomes (efficacy or tolerability) per category, the effect sizes will be combined to produce a single composite effect size (SMD or RR) using the method in Borenstein et al (2009).[4] We will assume a within-cluster correlation between outcomes of $r = 0.45$. We will make this assumption because studies almost never report the inter-correlations between their outcome measures. $r = 0.45$ is the 95th percentile correlation across 708 meta-analytically-derived correlations in psychology between different constructs.[5] We will use the 95th percentile since it gives a more conservative estimate of the correlation between similar outcomes within studies. This is because smaller correlations lower the variance of combined effect sizes.[4]

If a continuous outcome is reported alongside its dichotomous outcome (e.g., response rate based on ≥ 50% reduction in the scale), only the continuous outcome will be extracted. This is due to the redundancy and the lower statistical power of dichotomized continuous scales. In order to combine continuous (e.g., self-report scale of impulsivity) and dichotomous (e.g., number of aggressive episodes) efficacy data within a symptom category (e.g., behavioural dyscontrol) we will perform the following procedure: an odds ratio (OR) will be calculated for the dichotomous outcome and converted to Cohen's *d* using established equations.[4] We will apply the *J* correction factor to convert the Cohen's *d* to Hedges' *g* (the SMD).[5] This will be combined with the SMD of the continuous outcome(s) within the symptom category. Note, the RR is preferred over the OR for RCTs and cohort studies. The OR approximates the RR when the incidence of the outcome is low (< 10%). As the incidence increases, the OR overestimates the RR when it is greater than 1 and underestimates the RR when it's less than 1.[6] To the best of our knowledge there are no established procedures to convert RRs directly to SMDs. Therefore, while imperfect, we will use this procedure with dichotomous outcomes given the advantages of combining multiple outcomes into a single effect size per symptom category.

For peer review only - http://bmjopen.bmj.com/site/about/guidelines.xhtml

8. Data extraction (selection and coding)

1. **Selection of studies:** two reviewers will independently screen the titles and abstracts of the search results to determine potential eligibility. Full-text reports of the potentially eligible reports will then be retrieved and assessed by two reviewers using our inclusion/exclusion criteria to determine eligibility. Disagreements at either the screening or full-text review stages of the selection phase will be resolved through consensus between the two reviewers and, if necessary, a third reviewer. Decisions regarding eligibility will be documented.
2. **Data extraction:** Two reviewers will independently extract the main outcomes and additional outcomes from the reports into standardized electronic databases. Discrepancies in data extraction will be resolved through consensus between the two authors and, if necessary, a third author.

9. Risk of bias assessment

Risk of bias in the randomized controlled trials (RCTs) will be assessed using the Cochrane Risk of Bias 2 (ROB 2). Risk of bias in the non-randomized primary studies will be assessed using the Cochrane Risk of Bias in Non-randomized Studies of Interventions (ROBINS-I). Risk of bias will be assessed by two independent reviewers. All disagreements will be resolved through consensus between the two reviewers and a third reviewer if necessary. ROBINS-I requires listing in the protocol the confounding domains and co-interventions. Confounding domains and co-interventions are those for which adjustment is expected to lead to a clinically important change in the estimated effect of the intervention. We defined the following:

1. **Confounding domains:** age, sex, race/ethnicity, comorbidities, socioeconomic status.
2. **Co-interventions:** concurrent psychotherapy or pharmacotherapy.

10. Strategy for data synthesis

The characteristics of the included primary studies will be summarized in a tabular form using the PICO format along with a narrative summary of the results with the effect sizes of the efficacy/tolerability data when available. When appropriate, quality of evidence will be assessed using the GRADE approach.[7] The narrative summaries of the opinions from the non-primary studies will be summarized in tabular form.

For peer review only - http://bmjopen.bmj.com/site/about/guidelines.xhtml

APPENDIX C

The following pivotal guidelines, psychotherapy manuals, book chapters, and articles will be reviewed as part of the qualitative assessment in order to extract opinions relevant to our key questions.

Guidelines

1. National Institute for Health and Clinical Excellence (NICE) guideline on treatment and management: Borderline personality disorder[8]
2. American Psychiatric Association (APA) practice guideline for the treatment of patients with borderline personality disorder[9]
3. National Health and Medical Research Council (NHMRC) clinical practice guideline for the management of borderline personality disorder[10]
4. World Federation of Societies of Biological Psychiatry (WFSBP) Guidelines for biological treatment of personality disorders[11]
5. The project BETA guidelines[12]
6. Garriga et al (2016) Assessment and management of agitation in psychiatry: Expert consensus. *The World Journal of Biological Psychiatry*.[13]

Psychotherapy manuals

1. Linehan (1993) Cognitive-behavioral treatment of borderline personality disorder.[14]
2. Bateman & Fonagy (2016) Mentalization-based treatment for personality disorders: A practical guide.[15]
3. Yeomans et al (2015) Transference focused psychotherapy for borderline personality disorder: A Clinical Guide.[16]
4. Kernberg (1984) Severe personality disorders: Psychotherapeutic Strategies.[17]
5. Gunderson & Links (2008) Borderline personality disorder: A clinical guide.[1]
6. Gunderson & Links (2014) Handbook of good psychiatric management for borderline personality disorder.[2]
7. Arntz & van Genderen (2021) Schema therapy for borderline personality disorder, 2nd Ed.[18]
8. Davidson (2008) Cognitive therapy for personality disorders: A guide for clinicians, 2nd Ed.[19]
9. Black & Blum (2016) Systems training for emotional predictability and problem solving for borderline personality disorder: Implementing STEPPS around the globe.[20]
10. Dawson & MacMillan (1993) Relationship management of the borderline patient: From understanding to treatment.[21]
11. Livesley (2003) Practical management of personality disorder.[22]
12. Livesley (2017) Integrated modular treatment for borderline personality disorder: A practical guide to combining effective treatment methods.[23]

Other books

1. Gabbard (2014) Psychodynamic psychiatry in clinical practice, 5th Ed.[24]
2. Gabbard & Wilkinson (2000) Management of countertransference with borderline patients.[25]

For peer review only - http://bmjopen.bmj.com/site/about/guidelines.xhtml

3. Choi-Kain & Gunderson (2019) Applications of good psychiatric management for borderline personality disorder.[26]
4. Sonley & Choi-Kain (2020) Good psychiatric management and dialectical behavior therapy: A clinician's guide to integration and stepped care.[27]

Book chapters

1. Hong & Casher (2020) The inpatient with borderline personality disorder. In: *Manual of Inpatient Psychiatry, 2nd Ed*.[28]
2. Hong et al (2020) Personality disorders. In: *Emergency Psychiatry: Principles and Practice, 2nd Ed*.[29]
3. Hong et al (2019) Traitements dans les services d'urgence. In: *Trouble de la Personnalite Borderline: Pratiques Therapeutiques*.[30]
4. Curry & Riddle (2021) Personality disorders in the emergency department. In: *Behavioral Emergencies for Healthcare Providers*.[31]

Published papers

1. Hong (2016) Borderline personality disorder in the emergency department: good psychiatric management. *Harvard Review of Psychiatry*.[3]
2. Hong et al (2019) Antisocial and borderline personality disorders in the emergency department: conceptualizing and managing "Malingered" or "Exaggerated" symptoms. *Current Behavioral Neuroscience Reports*.[32]
3. Shaikh et al (2017). Patients with borderline personality disorder in emergency departments. *Frontiers in Psychiatry*.[33]
4. Broadbear et al (2022). Emergency department utilisation by patients with a diagnosis of borderline personality disorder: An acute response to a chronic disorder. *Emergency Medicine Australasia*.[34]
5. Cases et al (2020). Evidence of practice gaps in emergency psychiatric care for borderline personality disorder: how can this be explained? *BMC Psychiatry*.[35]
6. Acres et al (2021). From the community to the emergency department: A study of hospital emergency department nursing practices from the perspective of carers of a loved one with Borderline Personality Disorder. *Health & social care in the community*.[36]
7. Paris (2002) Chronic suicidality among patients with borderline personality disorder. *Psychiatric Services*.[37]
8. Paris (2004) Understanding self-mutilation in borderline personality disorder. *Harvard Review of Psychiatry*.[38]
9. Paris (2004) Half in love with easeful death: The meaning of chronic suicidality in borderline personality disorder. *Harvard Review of Psychiatry*.[39]
10. Paris (2004) Is hospitalization useful for suicidal patients with borderline personality disorder? *Journal of Personality Disorders*.[40]
11. Paris (2006) Predicting and preventing suicide: Do we know enough to do either? . *Harvard Review of Psychiatry*.[41]
12. Paris (2021) Can we predict or prevent suicide?: An update. *Preventative Medicine*.[42]
13. Goodman et al (2012) Suicidal risk and management in borderline personality disorder. *Current Psychiatry Reports*.[43]

For peer review only - http://bmjopen.bmj.com/site/about/guidelines.xhtml

References

1. Gunderson J, Links P. Borderline Personality Disorder: A Clinical Guide. Washington, DC: American Psychiatric Association Publishing 2008.
2. Gunderson J, Links P. Handbook of Good Psychiatric Management for Borderline Personality Disorder. Washington, DC: American Psychiatric Association Publishing 2014.
3. Hong V. Borderline personality disorder in the emergency department: good psychiatric management. *Harv Rev Psychiatry* 2016;24(5):357-66. doi: 10.1097/HRP.0000000000000112
4. Borenstein M, Hedges LV, Higgins JP, et al. Introduction to Meta-analysis. Chichester, UK: Wiley 2009.
5. Gignac GE, Szodorai ET. Effect size guidelines for individual differences researchers. *Personality and individual differences* 2016;102:74-78.
6. Zhang J, Kai FY. What's the relative risk?: A method of correcting the odds ratio in cohort studies of common outcomes. *JAMA* 1998;280(19):1690-91.
7. Guyatt GH, Oxman AD, Vist GE, et al. GRADE: an emerging consensus on rating quality of evidence and strength of recommendations. *BMJ* 2008;336(7650):924. doi: 10.1136/bmj.39489.470347.AD
8. Health NCCfM. Borderline Personality Disorder: The NICE Guideline on Treatment and Management. National Clinical Practice Guideline No. 78: The British Psychological Society and The Royal College of Psychiatrists, 2009.
9. Association AP. Practice guideline for the treatment of patients with borderline personality disorder: American Psychiatric Association Practice Guidelines 2001.
10. Smith M, Chanen A, Ford F, et al. Clinical practice guideline for the management of borderline personality disorder. Melbourne: National Health and Medical Research Council (NHMRC), 2012.
11. Herpertz SC, Zanarini M, Schulz CS, et al. World Federation of Societies of Biological Psychiatry (WFSBP) guidelines for biological treatment of personality disorders. *The World Journal of Biological Psychiatry* 2007;8(4):212-44.
12. Holloman Jr GH, Zeller SL. Overview of Project BETA: best practices in evaluation and treatment of agitation. *Western Journal of Emergency Medicine* 2012;13(1):1.
13. Garriga M, Pacchiarotti I, Kasper S, et al. Assessment and management of agitation in psychiatry: Expert consensus. *The World Journal of Biological Psychiatry* 2016;17(2):86-128. doi: 10.3109/15622975.2015.1132007
14. Linehan M. Cognitive-behavioral Treatment of Borderline Personality Disorder. New York City, NY: Guilford Press 1993.
15. Bateman A, Fonagy P. Mentalization based treatment for personality disorders: A practical guide. Oxford, UK: Oxford University Press 2016.
16. Yeomans FE, Clarkin JF, Kernberg OF. Transference-Focused Psychotherapy for Borderline Personality Disorder: A Clinical Guide. Arlington, VA: American Psychiatric Association Publishing 2015.
17. Kernberg OF. Severe Personality Disorders: Psychotherapeutic Strategies. New Haven, CT: Yale University Press 1984.
18. Arntz A, Van Genderen H. Schema therapy for borderline personality disorder. West Sussex, UK: John Wiley & Sons, Ltd 2021.
19. Davidson K. Cognitive therapy for personality disorders: A guide for clinicians. 2nd ed. East Sussex, UK: Routledge 2008.

For peer review only - http://bmjopen.bmj.com/site/about/guidelines.xhtml

20. Black DW, Blum N. Systems training for emotional predictability and problem solving for borderline personality disorder: Implementing STEPPS around the globe. New York City, NY: Oxford University Press 2017.
21. Dawson DL, MacMillan HL. Relationship management of the borderline patient: from understanding to treatment. New York City, NY: Routledge 1993.
22. Livesley J. Practical Management of Personality Disorder. New York City, NY: Guilford Press 2003.
23. Livesley J. Integrated Treatment for Borderline Personality Disorder: A Practical Guide to Combining Effective Treatment Methods. Cambridge, UK: Cambridge University Press 2017.
24. Gabbard GO. Psychodynamic psychiatry in clinical practice. 5th ed. Arlington, VA: American Psychiatric Association Publishing 2014.
25. Gabbard GO, Wilkinson SM. Management of countertransference with borderline patients. Northvale, New Jersey: Jason Aronson Inc. 2000.
26. Choi-Kain LW, Gunderson JG. Applications of good psychiatric management for borderline personality disorder: a practical guide. Washington, DC: American Psychiatric Association Publishing 2019.
27. Sonley AK, Choi-Kain LW. Good psychiatric management and dialectical behavior therapy: A clinician's guide to integration and stepped care. Washington, DC: American Psychiatric Association Publishing 2021.
28. Hong V, Casher M. The inpatient with borderline personality disorder. In: Casher M, Bess J, eds. Manual of Inpatient Psychiatry. 2nd ed. Cambridge, UK: Cambridge University Press 2020.
29. Hong V, Reddy P, Entenman S. Personality Disorders. In: Glick R, Zeller S, Berlin J, eds. Emergency Psychiatry: Principles and Practice. 2nd ed. Philadelphia, PA: Wolters Kluwer 2020.
30. Hong V, Kozada K, Shobassy A. Traitements dans les services d'urgence. In: Kolly S, Charbon P, Kramer U, eds. Trouble de la Personnalite Borderline: Pratiques Therapeutiques France: Elsevier Masson SAS 2019.
31. Curry A, Riddle M. Personality Disorders in the Emergency Department. In: Zun L, Nordstrom K, Wilson M, eds. Behavioral Emergencies for Healthcare Providers. 2nd ed. Switzerland: Springer Nature 2021.
32. Hong V, Pirnie L, Shobassy A. Antisocial and borderline personality disorders in the emergency department: conceptualizing and managing "Malingered" or "Exaggerated" symptoms. *Current Behavioral Neuroscience Reports* 2019;6(4):127-32.
33. Shaikh U, Qamar I, Jafry F, et al. Patients with borderline personality disorder in emergency departments. *Frontiers in psychiatry* 2017;8:136.
34. Broadbear JH, Rotella J-A, Lorenze D, et al. Emergency department utilisation by patients with a diagnosis of borderline personality disorder: An acute response to a chronic disorder. *Emerg Med Australas* 2022;34(5):731-37.
35. Cases C, Lafont Rapnouil S, Gallini A, et al. Evidence of practice gaps in emergency psychiatric care for borderline personality disorder: how can this be explained? *BMC Psychiatry* 2020;20(1):476. doi: 10.1186/s12888-020-02892-7
36. Acres K, Loughhead M, Procter N. From the community to the emergency department: A study of hospital emergency department nursing practices from the perspective of carers

For peer review only - http://bmjopen.bmj.com/site/about/guidelines.xhtml

of a loved one with Borderline Personality Disorder. *Health Soc Care Community* 2022;30(5):1789-97.

37. Paris J. Chronic Suicidality Among Patients With Borderline Personality Disorder. *Psychiatr Serv* 2002;53(6):738-42. doi: 10.1176/appi.ps.53.6.738
38. Paris J. Understanding Self-mutilation in Borderline Personality Disorder. *Harv Rev Psychiatry* 2005;13(3):179-85. doi: 10.1080/10673220591003614
39. Paris J. Half in Love with Easeful Death: The Meaning of Chronic Suicidality in Borderline Personality Disorder. *Harv Rev Psychiatry* 2004;12(1):42-48. doi: 10.1080/10673220490279161
40. Paris J. Is Hospitalization Useful for Suicidal Patients with Borderline Personality Disorder? *J Personal Disord* 2004;18(3):240-47. doi: 10.1521/pedi.18.3.240.35443
41. Paris J. Predicting and Preventing Suicide: Do We Know Enough to Do Either? *Harv Rev Psychiatry* 2006;14(5):233-40. doi: 10.1080/10673220600968662
42. Paris J. Can we predict or prevent suicide?: An update. *Prev Med* 2021;152(Pt 1):106353. doi: 10.1016/j.ypmed.2020.106353 [published Online First: 2021/09/21]
43. Goodman M, Roiff T, Oakes AH, et al. Suicidal Risk and Management in Borderline Personality Disorder. *Curr Psychiatry Rep* 2012;14(1):79-85. doi: 10.1007/s11920-011-0249-4

For peer review only

For peer review only - http://bmjopen.bmj.com/site/about/guidelines.xhtml
